# The *Gynandropsis gynandra* genome provides insights into whole-genome duplications and the evolution of C_4_ photosynthesis in Cleomaceae

**DOI:** 10.1093/plcell/koad018

**Published:** 2023-01-24

**Authors:** Nam V Hoang, E O Deedi Sogbohossou, Wei Xiong, Conor J C Simpson, Pallavi Singh, Nora Walden, Erik van den Bergh, Frank F M Becker, Zheng Li, Xin-Guang Zhu, Andrea Brautigam, Andreas P M Weber, Jan C van Haarst, Elio G W M Schijlen, Prasad S Hendre, Allen Van Deynze, Enoch G Achigan-Dako, Julian M Hibberd, M Eric Schranz

**Affiliations:** Biosystematics Group, Wageningen University, Droevendaalsesteeg 1, 6708PB Wageningen, The Netherlands; Biosystematics Group, Wageningen University, Droevendaalsesteeg 1, 6708PB Wageningen, The Netherlands; Laboratory of Genetics, Biotechnology and Seed Science (GbioS), Faculty of Agronomic Sciences, University of Abomey-Calavi, BP 2549 Abomey-Calavi, Republic of Benin; Biosystematics Group, Wageningen University, Droevendaalsesteeg 1, 6708PB Wageningen, The Netherlands; Department of Plant Sciences, University of Cambridge, Cambridge CB2 3EA, UK; Department of Plant Sciences, University of Cambridge, Cambridge CB2 3EA, UK; Biosystematics Group, Wageningen University, Droevendaalsesteeg 1, 6708PB Wageningen, The Netherlands; Centre for Organismal Studies, Heidelberg University, 69120 Heidelberg, Germany; Biosystematics Group, Wageningen University, Droevendaalsesteeg 1, 6708PB Wageningen, The Netherlands; Laboratory of Genetics, Wageningen University and Research, Droevendaalsesteeg 1, 6708PB Wageningen, The Netherlands; Department of Integrative Biology, The University of Texas at Austin, Austin, TX 78712, USA; State Key Laboratory for Plant Molecular Genetics, Center of Excellence for Molecular Plant Sciences, Chinese Academy of Sciences, Shanghai 200032, China; Faculty of Biology, Bielefeld University, 33501 Bielefeld, Germany; Cluster of Excellence on Plant Science (CEPLAS), Institute of Plant Biochemistry, Heinrich Heine University Düsseldorf, 40225 Düsseldorf, Germany; Business Unit Bioscience, Wageningen University and Research, Droevendaalsesteeg 1, 6708PB Wageningen, The Netherlands; Business Unit Bioscience, Wageningen University and Research, Droevendaalsesteeg 1, 6708PB Wageningen, The Netherlands; African Orphan Crops Consortium (AOCC), World Agroforestry (ICRAF), Nairobi 00100, Kenya; African Orphan Crops Consortium (AOCC), World Agroforestry (ICRAF), Nairobi 00100, Kenya; Seed Biotechnology Center, University of California, Davis, California 95616, USA; Laboratory of Genetics, Biotechnology and Seed Science (GbioS), Faculty of Agronomic Sciences, University of Abomey-Calavi, BP 2549 Abomey-Calavi, Republic of Benin; Department of Plant Sciences, University of Cambridge, Cambridge CB2 3EA, UK; Biosystematics Group, Wageningen University, Droevendaalsesteeg 1, 6708PB Wageningen, The Netherlands

## Abstract

*Gynandropsis gynandra* (Cleomaceae) is a cosmopolitan leafy vegetable and medicinal plant, which has also been used as a model to study C_4_ photosynthesis due to its evolutionary proximity to C_3_ Arabidopsis (*Arabidopsis thaliana*). Here, we present the genome sequence of *G. gynandra*, anchored onto 17 main pseudomolecules with a total length of 740 Mb, an N50 of 42 Mb and 30,933 well-supported gene models. The *G. gynandra* genome and previously released genomes of C_3_ relatives in the Cleomaceae and Brassicaceae make an excellent model for studying the role of genome evolution in the transition from C_3_ to C_4_ photosynthesis. Our analyses revealed that *G. gynandra* and its C_3_ relative *Tarenaya hassleriana* shared a whole-genome duplication event (*Gg-α*), then an addition of a third genome (*Th-α,* +1×) took place in *T. hassleriana* but not in *G. gynandra*. Analysis of syntenic copy number of C_4_ photosynthesis-related gene families indicates that *G. gynandra* generally retained more duplicated copies of these genes than C_3_*T. hassleriana*, and also that the *G. gynandra* C_4_ genes might have been under positive selection pressure. Both whole-genome and single-gene duplication were found to contribute to the expansion of the aforementioned gene families in *G. gynandra*. Collectively, this study enhances our understanding of the polyploidy history, gene duplication and retention, as well as their impact on the evolution of C_4_ photosynthesis in Cleomaceae.

## Teaser

The *Gynandropsis gynandra* genome sequence facilitates comparative phylogenomics and sheds light on the whole-genome duplication history and evolution of C_4_ photosynthesis in Cleomaceae.

**IN A NUTSHELL**

**Background:** The Cleomaceae is the sister family to the Brassicaceae (including the model species Arabidopsis and *Brassica* crops). The Cleomaceae contains species with different types of photosynthesis, including C_3_, C_4,_ and C_3_–C_4_ intermediate plants. As the Brassicaceae family does not have a true C_4_ species, the Cleomaceae serves as a valuable model system for photosynthesis research that aims to improve crops. The Cleomaceae also includes several economically important leafy, medicinal, and ornamental plants. Despite its scientific and economical importance, few genetic and genomic resources exist for the Cleomaceae.

**Question:** How did the Cleomaceae family evolve since its divergence from the Brassicaceae? What factors contributed to the evolution of C_4_ photosynthesis in Cleomaceae?

**Findings:** We generated a reference genome for the C_4_ species *Gynandropsis gynandra* that facilitates comparative genomics with its C_3_ relative, *Tarenaya hassleriana*, to elucidate the family polyploidy history and evolution of C_4_ photosynthesis in the Cleomaceae. These species evolved through step-wise ancient polyploidy events, in which a whole-genome duplication event (*Gg-α,* 2x) occurred first, followed by an addition of a third genome (*Th-α,* +1×) to *T. hassleriana* but not to *G. gynandra*. The evolution of C_4_ photosynthesis in the Cleomaceae resulted from a series of processes, including differential duplication, retention, recruitment, and expression modification of C_4_-related genes. This led to the preferential expression of these genes in leaf mesophyll or bundle sheath cells depending on their functions.

**Next steps:** Future efforts will focus on developing genomic resources for species of different photosynthesis types in the Cleomaceae. This will allow a more systematic analysis of the family history and trait evolution. It will also facilitate the study of important gene families related to plant physiological and anatomical changes involved in the transition from C_3_ to C_4_ photosynthesis. This can help to engineer C_4_ photosynthesis into non-C_4_ crops.

## Introduction

*Gynandropsis gynandra* (2*n* = 34, shares the common name “spider plant” with a number of unrelated species) belongs to the Cleomaceae, the sister family of the Brassicaceae (Hugh et al., 2011), and is grown as a leafy vegetable but also as a medicinal plant (Sogbohossou et al., 2018). *Gynandropsis gynandra* is an essentially cosmopolitan species found across Africa, Asia, the Middle East, and Australasia and has been introduced to the Caribbean, Southern and Northern America, and Central and Northern Europe (Chweya and Mnzava, 1997). Despite the wide distribution range of the species, *G. gynandra* is considered an “orphan” or “neglected” crop because of the lack of research efforts to develop genetic and genomic resources (Achigan-Dako et al., 2021).

Developing genomic resources for *G. gynandra* would open up diverse research avenues, three of which we highlight. First, the species is an economically important leafy vegetable in several communities around the world and a source of provitamin A, vitamins C and E, calcium, and iron (Van den Heever and Venter, 2007; Sogbohossou et al., 2019). It also contains diverse health-promoting compounds including glucosinolates, flavonoids, and phenylpropanoids (Neugart et al., 2017; Omondi et al., 2017b). Thus, owing to its potential to address hunger and malnutrition and to be a source of economic revenue, the species has been included in the list of 101 crops by the African Orphan Crops Consortium (AOCC) (Hendre et al., 2019; Jamnadass et al., 2020). The genome sequence of the species would, therefore, represent an important resource for breeding programs targeting traits ranging from higher leaf yield to increased secondary metabolite production and disease resistance (Achigan-Dako et al., 2021). Second, *G. gynandra* is a C_4_ plant and the Cleomaceae family contains both C_3_ and C_4_ plants, as well as C_3_–C_4_ intermediates (Marshall et al., 2007; Feodorova et al., 2010; Koteyeva et al., 2011; Bayat et al., 2018; Parma et al., 2022). Due to its evolutionary proximity and being the closest C_4_ species to the well-studied *Arabidopsis thaliana* (Brassicaceae) (Schranz and Mitchell-Olds, 2006; Edger et al., 2018), *G. gynandra* has been used as a C_4_ model (Brown et al., 2005; Newell et al., 2010). It is often compared with its closely related species C_3_*Tarenaya hassleriana*, for which the genome sequence is available (Bräutigam et al., 2010; Cheng et al., 2013; van den Bergh et al., 2014; Huang et al., 2021). Third, the Cleomaceae and the Brassicaceae are sister clades in the Brassicales order that share several older ancient polyploidy events including the *At-γ* whole-genome triplication (WGT = hexaploidy) and the Brassicales-specific *At-β* whole-genome duplication (WGD = tetraploidy) (Jaillon et al., 2007; Ming et al., 2008). However, the *At-α* WGD event occurred at the origin of the Brassicaceae (Mabry et al., 2020; Walden et al., 2020) and is not shared with the Cleomaceae (Schranz and Mitchell-Olds, 2006; Mabry et al., 2020). Evidence for independent polyploidy events has been found for the Cleomaceae, including the characterization of the *Th-α* WGT event (Schranz and Mitchell-Olds, 2006; Cheng et al., 2013; van den Bergh et al., 2014; Mabry et al., 2020). So far, because of the limited genomic resources available, the *Th-α* event in Cleomaceae was only reported in representative species including *T. hassleriana* based on whole-genome sequence (Cheng et al., 2013); and *G. gynandra, Cleomaceae* sp., *Melidiscus giganteus*, and *Sieruela monophyla* based on transcriptome data (van den Bergh et al. 2014; Mabry et al., 2020; Huang et al., 2021). With the genomes of more species from the Cleomaceae becoming available, the impact of polyploidy on species and trait evolution can be investigated at a broader scale, for example, the impact of WGD on the transition from C_3_ to C_4_ photosynthesis among the C_3_, C_3_–C_4_ intermediate and C_4_ species.

C_4_ photosynthesis is thought to have evolved as an adaptation to environmental conditions including high light intensity, high temperature, low water availability, and CO_2_ deficiency (Gowik and Westhoff, 2010). As a result, plants with C_4_ photosynthesis can achieve up to 50% higher photosynthetic efficiency compared to those with C_3_ photosynthesis in certain environments, for example, in warm, sunny, and dry regions (Sage, 2004; Bayat et al., 2018). This is mostly due to their unique mode of CO_2_ fixation in which the biochemical reactions are spatially separated between two cell types, typically the mesophyll (M) and bundle sheath (BS) cells (Hatch, 1971). From an evolutionary perspective, C_4_ photosynthesis is an example of convergent evolution in which the trait is thought to have evolved independently at least 60 times within the angiosperms (Sage et al., 2011; Bayat et al., 2018). The evolution of C_4_ photosynthesis is thought to be facilitated by both WGD and single-gene duplication (Monson, 2003; Wang et al., 2009b; Williams et al., 2012; Ren et al., 2018). The contribution of gene duplication and neo-/subfunctionalization to the evolution of different C_4_ photosynthesis subtypes was studied in several species including sorghum (*Sorghum bicolor*), maize (*Zea mays*), and other monocots (Wang et al., 2009b; Emms et al., 2016; Bianconi et al., 2018), *Flaveria* (Schulze et al., 2013), and Cleomaceae (van den Bergh et al., 2014; Huang et al., 2021).

Because the Cleomaceae and Brassicaceae families shared several ancient polyploid events, they have a high level of genome synteny and collinearity (Schranz and Mitchell-Olds, 2006; Cheng et al., 2013). This positions the C_4_ species *G. gynandra* to be a model for the comparative functional and evolutional analyses of C_4_ photosynthesis to utilize the rich genetic resources available from the model plant *A. thaliana* and *Brassica* crops of the Brassicaceae. However, to date, genomic studies on C_4_ gene evolution in Cleomaceae have mostly been based on transcriptome-derived sequences (Külahoglu et al., 2014; van den Bergh et al., 2014; Huang et al., 2021). These studies, while providing valuable information, cannot account for the contribution of different gene duplication modes or genome syntenic relationships.

In this study, we present the genome sequence of the C_4_ species *G. gynandra* and analyses of WGD/WGT history and the contribution of different gene duplication modes to the evolution of C_4_ photosynthesis in Cleomaceae. We show that the genomes of *G. gynandra* and its C_3_ relative *T. hassleriana* underwent a common WGD event (termed as *Gg-α*), and then another genome was added to *T. hassleriana* (*Th-α,* +1×) but not to *G. gynandra*. The *Gg-α* WGD event is also likely shared with other species in the Cleomaceae family. Analysis of syntenic copy number of gene families that encode key enzymes and transporters in the C_4_ cycle reveals that *G. gynandra* generally contains more copies of these genes than *T. hassleriana*, and that *G. gynandra* genes might have been under positive selection. We also show that both whole-genome and single-gene duplication contributed to the expansion of C_4_ gene families in *G. gynandra*. Our results suggest that C_4_ photosynthesis likely evolved in *G. gynandra* but not in *T. hassleriana* as a result of differential gene duplication and gene retention. Comparative gene expression analysis highlights subgenome dominance and the upregulation of the recruited C_4_ duplicated gene copies that function in a tissue- and cell type-specific manner in *G. gynandra*. Altogether, our data provide valuable information about the history of WGD/WGT and the impact of genome and gene duplication as well as gene retention on the evolution of C_4_ photosynthesis in the Cleomaceae family.

## Results

### Assembly and annotation of the genome of *G. gynandra*: a model for C_4_ photosynthesis

**Figure 1:**
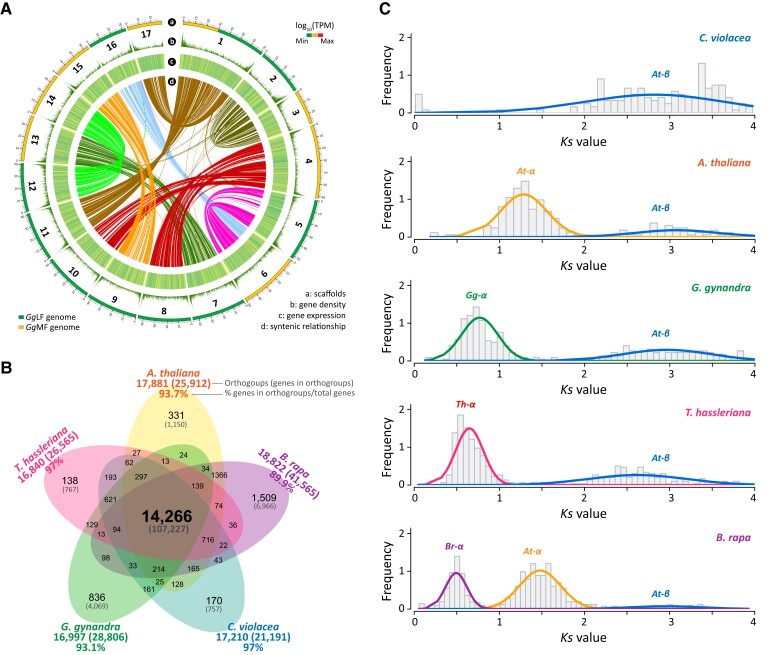
The genome sequence of *G. gynandra*, intraspecies synteny, gene orthogroup clustering, and whole-genome duplication events. A, Circos plot showing largest 17 pseudomolecules of the *G. gynandra* genome assembly (track a) including two subgenomes *Gg*LF (green) and *Gg*MF (yellow), gene density (track b), the expression level of the predicted gene models (track c), and intraspecies syntenic blocks (*minspan = 4 genes*) among the scaffolds analyzed by MCscan (track d). Gene densities were estimated by a window of 100 kb. Gene expression was calculated for each window of 100 kb, using leaf developmental (stages Leaf_0 to Leaf_5) transcriptome data from Külahoglu et al. (2014), quoted as log_10_(average TPM). Ribbon links in the inner track convey syntenic regions between two pseudomolecules and generally show a clear 2:2 syntenic pattern. Scaffold length is in Mb. B, Venn diagram illustrating the commonly shared and unique orthogroups from *G. gynandra, C. violacea, T. hassleriana, A. thaliana*, and *B. rapa*. Numbers in brackets denote the genes included in the orthogroups. Percentages were calculated based on the total genes annotated in each genome. C, Whole-genome duplication (WGD) events identified in different species by fitting the *Ks* distributions for WGD-derived gene pairs using Gaussian Mixture Models (GMMs). *Ks* peaks corresponding to *At-β* (commonly shared), *At-α* in *A. thaliana* and *B. rapa, Gg-α* in *G. gynandra, Th-α* in *T. hassleriana* and *Br-α* in *B. rapa.* Only *Ks* ≤ 4 were included in this analysis.

The estimated haploid genome size of the *G. gynandra* line “GYN” used in our study is 930.3 Mb (Supplemental Figure 1), which is close to the range of 1.1–1.2 Gb (2*n* = 34) previously reported for different accessions using flow cytometry (Omondi et al., 2017a; Parma et al., 2022). This genome is relatively large compared with that of its closely related species from the Cleomaceae family including *T. hassleriana* (*Th*): ∼290 Mb (Cheng et al., 2013), *Cleome violacea* (*Cv*): ∼280 Mb (Wing et al., 2013), and other species in the *Tarenaya* cluster recently reported in Parma et al. (2022). To construct the genome assembly of *G. gynandra*, we used materials from the line “GYN” (inbred for four generations) for whole-genome sequencing at a total of 68–125× genome coverage through a combined approach of Illumina sequencing, 10× Genomics sequencing and chromatin conformation capture Hi-C technologies (Supplemental Table 1 and see **Methods** for more information). Here, we obtained three assembly versions (v1.0 to v3.0, corresponding to the technologies used) with a size ranging from 740 Mb to 1.04 Gb (Supplemental Table 2). The use of 10× Genomics and Hi-C technologies significantly improved scaffold N50 (293 kb to 41.9 Mb) and BUSCO (Benchmarking Universal Single-Copy Orthologs) completeness score slightly (98.1%–98.2%) while reduced assembly size (1.04 Gb to 740 Mb). This size reduction did not affect the gene content of our assemblies (through BUSCO scores) and three assembly versions maintained a high mapping-back rate of Illumina raw reads (98% for v1.0 and 97.5% for v2.0 and v3.0) (Supplemental Table 3). We conclude that the differences between predicted and assembled genome size are largely due to the high repetitive content in the *G. gynandra* genome (Beric et al., 2021). The final version of the genome (v3.0) has 616 scaffolds with an N50 of 41.9 Mb and a total length of 740 Mb (Table 1, Supplemental Figure 2 and Supplemental Table 4). The majority of the assembly is anchored onto 17 pseudomolecules (superscaffolds) that account for ∼99% of the assembly (Figure 1A) which is consistent with the previously reported chromosome number for *G. gynandra* (Omondi et al., 2017a). About 69% of the assembly are repetitive elements, of which long terminal repeat retrotransposons (LTR-RT) accounted for ∼42%, followed by DNA transposons (∼13%) (Supplemental Table 5).

**Table 1:** Summary statistics of the genome assembly and annotation of *G. gynandra*

Assembly v3.0 (chromosome level)	
Number of reported chromosomes (Omondi et al., 2017a)	2*n* = 34
Genome size predicted (Mb)	930.3
Number of scaffolds	616
Total assembled genome (Mb)	740
Longest scaffold (Mb)	71
Scaffolds N50 (Mb)	41.9
Scaffolds L50	8
GC content (%)	37.7
Number of pseudomolecules	17
Total length of pseudomolecules (Mb)	732.6
Genome in pseudomolecules (%)	99
Embryophyta 1,614 BUSCOs completeness (%)	98.2
Annotation and validation	
Number of gene models	30,933
Number of transcripts	33,748
Transcript N50 (bp)	1,524
Number of exons per gene	6.7
Embryophyta 1,614 BUSCOs completeness (%)	97.1
Genes in orthogroups (%)	93.1
Genes annotated with public databases (%)	91.2
Repetitive elements (Mb)	509.1
Repetitive elements (% genome)	68.8

Integration of the various gene prediction approaches resulted in 30,933 well-supported gene models and 33,748 transcripts (Supplemental Table 6) with completeness estimated to be 97.1% by BUSCO (Simão et al., 2015) (Supplemental Table 7). By mapping 18 *G. gynandra* transcriptome datasets derived from the major tissues/organs at different developmental stages (Külahoglu et al., 2014), we found 30,013 genes (97% total predicted genes) supported by the transcriptome data (TPM, transcripts per million transcripts, > 0, Figure 1A). A total of 28,209 of gene models (91.2% of total genes) matched with sequences or conserved motifs in at least one of the public protein databases (Supplemental Table 8), including 77.8% matching Swiss-Prot (O'Donovan et al., 2002), 88.8% with TrEMBL (O'Donovan et al., 2002), 80.2% with InterPro (Zdobnov and Apweiler, 2001), 60% with gene ontology (GO) (Ashburner et al., 2000), and 40.6% with Kyoto Encyclopedia of Genes and Genomes (KEGG) (Kanehisa and Goto, 2000).

Orthologous clustering of protein sequences of *G. gynandra* and four other Brassicaceae and Cleomaceae species (*A. thaliana*, *Brassica rapa*, *C. violacea*, and *T. hassleriana*) resulted in 28,806 *G. gynandra* genes (93.1% of total genes) being classified into 16,997 orthogroups (Figure 1B and Supplemental Table 9). Of these, 16,161 orthogroups (24,737 genes, 80% genes) were clustered with at least one of the four aforementioned genomes from the Brassicaceae and Cleomaceae. A total of 14,266 orthogroups was commonly shared among the five species, while 836 orthogroups were specific to the *G. gynandra* genome, more than to either the *C. violacea* (170) or *T. hassleriana* genomes (138). Since the *G. gynandra*-specific orthogroups might be important to the evolution and adaptation of this C_4_ species, we therefore analyzed the functions associated with these 836 orthogroups. A total of 4,069 genes were in these *G. gynandra*-specific orthogroups, of which, 2,010 and 1,395 genes were annotated with at least one InterPro domain and one GO term, respectively. GO enrichment analysis revealed several terms related to metabolic, cellular, and developmental processes, response to stimuli/stress and transcription regulation among the most significant terms (Supplemental Figure 3). Collectively, these results indicate that our genome assembly is of good quality. The availability of the genome of this C_4_ species and that of its C_3_ relatives (*C. violacea* and *T. hassleriana*) make them an interesting and useful model for studying comparative genome evolution that facilitates the transition from C_3_ to C_4_ photosynthesis.

### The *G. gynandra* genome underwent a WGD event after its divergence from Brassicaceae

The hexaploidy *Th-α* WGT event was previously reported in the genome of *T. hassleriana*, a closely related species to *G. gynandra* (Cheng et al., 2013). It has been hypothesized that the *G. gynandra* genome also experienced this WGT event (van den Bergh et al., 2014; Mabry et al., 2020). To determine whether the *Th-α* WGT event is also shared with *G. gynandra*, we analyzed the syntenic and colinear patterns in five representative Cleomaceae and Brassicaceae genomes. In this analysis, besides *G. gynandra* and *T. hassleriana*, we included *C. violacea*, another species from the Cleomaceae family that does not share either *At-α* with Brassicaceae or *Th-α* (Emery et al., 2018), for which whole-genome sequence is available (Wing et al., 2013). The inclusion of two Brassicaceae species, *A. thaliana* and *B. rapa*, allows comparison to the two recent well-studied genome polyploidy events in Brassicaceae, the tetraploidy *At-α* WGD (Bowers et al., 2003) and hexaploidy *Br-α* WGT (Wang et al., 2011).

**Figure 2:**
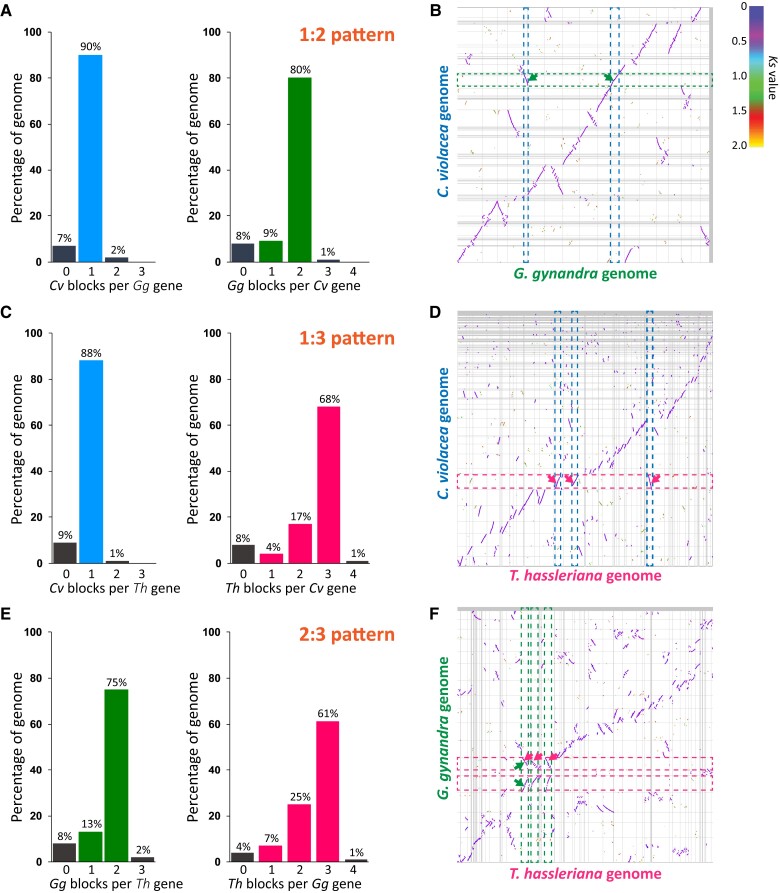
Comparative genomics of three Cleomaceae genomes. A, Ratio of syntenic depth between *C. violacea* and *G. gynandra*. Syntenic blocks of *C. violacea* per *G. gynandra* gene (left) and syntenic blocks of *G. gynandra* per *C. violacea* gene are shown suggesting a clear 1:2 pattern. B, Macrosynteny of the *C. violacea* and *G. gynandra* genomes. Blue and green dashed bands and arrows point to examples showing one syntenic block found in the *C. violacea* genome and two respective syntenic blocks in the *G. gynandra* genome. C, Ratio of syntenic depth between *C. violacea* and *T. hassleriana* showing a clear 1:3 pattern. D, Macrosynteny of the *C. violacea* and *T. hassleriana* genomes. Blue and red dashed bands and arrows point to examples showing one syntenic block found in the *C. violacea* genome and three respective syntenic blocks in the *T. hassleriana* genome per *C. violacea* block, respectively. E, Ratio of syntenic depth between *G. gynandra* and *T. hassleriana* showing a clear 2:3 pattern. F, Macrosynteny of the *G. gynandra* and *T. hassleriana* genomes. Green and red dashed bands and arrows point to examples showing two syntenic blocks found in the *G. gynandra* genome and three respective syntenic blocks in the *T. hassleriana* genome per *G. gynandra* block, respectively. Horizontal and vertical gray lines separate scaffolds. (B, D, F) Syntenic blocks were colored based on the *Ks* values of syntenic gene pairs between genomes. Color scale is provided at the top right corner. The names of the scaffolds in each genome are not shown. For the comparative genomics between *C. violacea* and Brassicaceae (*A. thaliana* and *B. rapa*, syntenic ratios of 1:2 and 1:6, respectively), see Supplemental Figure 7.

Overall, the *G. gynandra* genome showed extensive synteny and collinearity with other genomes from Cleomaceae and Brassicaceae (Figure 2 and Supplemental Figure 4). Our results also revealed that the *G. gynandra* genome shows evidence of an ancient WGD and not an ancient WGT as was previously reported for *T. hassleriana* (Supplemental Figure 5). Whole-genome intraspecies (self–self) syntenic comparison clearly displayed that most of the 17 pseudomolecules had a duplicated block on other scaffolds and generally a 2:2 syntenic pattern (Figure 1A and Supplemental Figure 5). We hereafter refer to this WGD event in *G. gynandra* as *Gg-α*. By fitting the distributions of *Ks* values (the ratio of the number of substitutions per synonymous site, representing sequence divergence time) for WGD-derived gene pairs from the five genomes by Gaussian Mixture Models (GMMs), we identified *Ks* peaks corresponding to *At-β* (commonly shared), and *At-α, Gg-α, Th-α* and *Br-α* in the respective genomes (Figure 1C and Supplemental Figure 6). Although the *Gg-α* event occurred at a similar time to *Th-α* in *T. hassleriana* and *Br-α* in *B. rapa*, the *Ks* peak in *G. gynandra* was slightly older than that of *T. hassleriana* and *B. rapa*. Additionally, there was only a single *Ks* peak corresponding to *At-β* found in *C. violacea*, confirming that it did not undergo the *Gg-α* or *Th-α* events. This is consistent with the self–self syntenic dotplot of *C. violacea* in which most of the detected syntenic gene pairs displayed greater *Ks* values (i.e. from the more ancient WGD events) than those detected in *G. gynandra* and *T. hassleriana* (Supplemental Figure 5).

We next studied the interspecies syntenic pattern and collinearity among the three Cleomaceae genomes of *C. violacea, G. gynandra*, and *T. hassleriana*. Since *C. violacea* did not experience the *Gg-α* or *Th-α* events, we hypothesized that it represents a “1×” genomic equivalent (1 GE) prior to the more recent and nested polyploidy events in the Cleomaceae. Indeed, pairwise comparisons of *C. violacea* vs. *G. gynandra*, *C. violacea* vs. *T. hassleriana*, and *G. gynandra* vs. *T. hassleriana* showed clear 1:2, 1:3, and 2:3 syntenic and collinear patterns, respectively (Figure 2, A–F). Around 80% and 68% of *C. violacea* genes had synteny to two and three blocks in *G. gynandra* and *T. hassleriana*, respectively (Figure 2, A and C). A greater number of genes in the two polyploid genomes (90% *G. gynandra* genes and 88% *T. hassleriana* genes) was found to be syntenic to one block in the *C. violacea* genome. Between the two of them, 61% of *G. gynandra* genes had synteny to three blocks in *T. hassleriana*, while 75% of *T. hassleriana* genes had synteny to two blocks in *G. gynandra* (Figure 2E). The results clearly suggest that, among the interspecies syntenic regions, the three Cleomaceae genomes display a pattern of 1:2:3 syntenic relationship for *C. violacea, G. gynandra*, and *T. hassleriana*, respectively. The *T. hassleriana* genome likely possesses an extra subgenome (3 GEs) compared to the *G. gynandra* genome (2 GEs).

### Both *G. gynandra* and *T. hassleriana* display biased gene fractionation in their subgenomes

**Figure 3:**
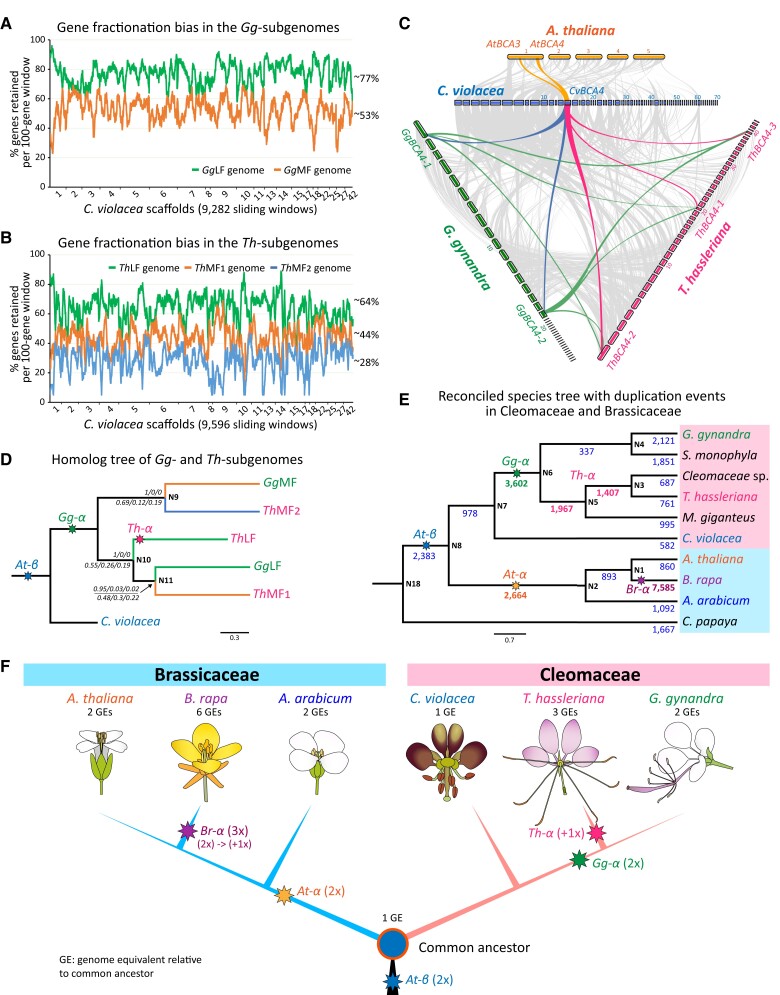
Subgenome fractionation bias and phylogenetic relationship of *G. gynandra*, Cleomaceae and Brassicaceae species. Gene fractionation bias in the two *Gg*-subgenomes, *Gg*LF and *Gg*MF of *G. gynandra* (A) and three Th-subgenomes, *Th*LF, *Th*MF_1_ and *Th*MF_2_ of *T. hassleriana* (B). For both genomes, the *C. violacea* genome was used as reference. Most of the synteny was detected within the largest 20 *Cv* scaffolds. Gene retention (%) was calculated for each sliding window of 100 genes across the *Cv* scaffolds. C, Macrosynteny and microsynteny patterns show that a genome region bearing gene *BCA4* (*BETA CARBONIC ANHYDRASE4*) in the *C. violacea* genome can be tracked to two regions in *A. thaliana* (yellow lines), two regions in the *G. gynandra* genome (blue lines), and three regions in the *T. hassleriana* genome (red lines). The background grey wedges highlight major syntenic blocks (*minspan = 30 genes*) between genomes. D, Phylogenetic relationships of subgenomes of *G. gynandra* and *T. hassleriana*. The tree was rooted using the *C. violacea* genome as outgroup. Supporting values at each node are posterior probability (upper) and quartet scores (lower). Tree was constructed using the species-tree approach based on 52 genes located on four syntenic blocks that were found across three species and their subgenomes. Branch length is in coalescence units. E, A reconciled species-tree of Cleomaceae and Brassicaceae species with their duplication events. The numbers provided at each node correspond to the gene duplications detected for each clade. Genome data from *A. arabicum*, *A. thaliana*, *B. rapa*, *C. violacea*, *G. gynandra*, *T. hassleriana*, and *C. papaya*; and transcriptome data of other species were used. Tree topology was adapted from the ASTRAL-III coalescent-based species phylogeny (Mabry et al., 2020). All branch supporting values are >0.7 posterior probability, and not shown. Branch length is in coalescence units. Tree was rooted using *C. papaya* as outgroup (see Methods for more information). “N” in (D and E) denotes “tree node”. F, Phylogenetic relationships between Brassicaceae and Cleomaceae species/genera and ancient polyploidy events detected in both lineages: the *At-β* (blue star) shared by Brassicaceae and Cleomaceae; the *At-α* (yellow star) shared by all Brassicaceae, the *Br-α* event (purple star) in *Brassica* spp.; in Cleomaceae, the *Gg-α* (green star) shared by *G. gynandra* and *T. hassleriana* and a potential genome addition (red star) in *T. hassleriana* explaining the *Th-α* triplication observed in the species.

Because our results suggested a 1:2:3 GE pattern among the three Cleomaceae species, we reconstructed two *G. gynandra* and three *T. hassleriana* subgenomes based on the syntenic blocks and orthologs/ohnologs between each of them and the *C. violacea* genome (as reference). The identified syntenic blocks and orthologs/ohnologs also allowed us to study the relationship among the subgenomes, which we present in the next section. Most of the syntenic blocks were detected within the 20 largest scaffolds in the *C. violacea* genome (Supplemental Data Set 1). For each syntenic block on these *C. violacea* scaffolds, two and three syntenic blocks were generally detected in the *G. gynandra* and *T. hassleriana* genomes, respectively. We then reordered these syntenic blocks into subgenomes based on the percentage of genes retained in each 100-gene window compared to that of the reference *C. violacea* genome. Two *G. gynandra* subgenomes (least fractionated and most fractionated, *Gg*LF and *Gg*MF, respectively, Figures 1A and 3A) and three *T. hassleriana* subgenomes (least, medium, and most fractionated, *Th*LF, *Th*MF_1_, and *Th*MF_2_, respectively, Figure 3B) were obtained. In general, *Gg*LF retained more genes compared to *Th*LF (77% vs. 64%), and *Gg*MF retained more genes compared to *Th*MF_1_ and *Th*MF_2_ (53% vs. 44% and 28%). The biased fractionation patterns as can be seen in the two Cleomaceae species are typically reported after an allopolyploidization (WGD by interspecific hybridization) (Thomas et al., 2006; Sankoff et al., 2010; Cheng et al., 2012); thus, we assume the *Gg-α*/*Th-α* to have been likely an allopolyploid event.

### Elucidation of polyploidy events and phylogenetic relationships of Cleomaceae and Brassicaceae

Elucidating ancient polyploidy events in related species of *G. gynandra* allows a better understanding of evolutionary relationships between them. Such information could facilitate translational genomics between *G. gynandra* and well-studied plants such as *Brassica* crops and *A. thaliana*. To this end, we first analyzed the relationships among duplicated gene copies of *BCA4* (*BETA CARBONIC ANHYDRASE4,* AT1G70410), which encodes an important enzyme that catalyzes the interconversion of CO_2_ and HCO_3_ in the first step of C_4_ photosynthesis (Hatch and Burnell, 1990; DiMario et al., 2016). Synteny analysis between *A. thaliana* and the three Cleomaceae genomes for the *BCA4* gene revealed one syntenic region in *C. violacea,* two in *A. thaliana*, two in *G. gynandra*, and three in *T. hassleriana* (Figure 3C). The phylogenetic relationship of these gene copies together with those from *Aethionema arabicum* and *B. rapa* is shown in Supplemental Figure 8A, which generally agrees with a species-tree constructed based on 2,223 single-copy orthogroups among the six species in Supplemental Figure 8B. We included *A. arabicum* and *B. rapa* in this analysis because the former represents the first divergent branch in Brassicaceae after the *At-α* WGD event following its separation from Cleomaceae (Schranz and Mitchell-Olds, 2006; Edger et al., 2018; Walden and Schranz, 2022), while the latter represents a polyploid genome resulted from a subsequent *Br-α* WGT event (Wang et al., 2011). It is noticeable that while the tree branch support values for Brassicaceae *BCA4* genes were generally high (posterior probability, *pp* > 0.9), those between *G. gynandra* and *T. hassleriana* were generally much lower (*pp* = 0.33–0.53). We also observed the low supporting values in an analysis of seven other selected genes that display 1 *Cv* : 2 *Gg* : 3 *Th* syntenic relationship among the three Cleomaceae species (Supplemental Figure 9). A possible reason for this could be that their speciation occurred very close to the *Gg-α* and *Th-α* events, as suggested by the overlapping distributions of *Ks* peaks corresponding to these events and species divergence (Supplemental Figure 6 and in Mabry et al. (2020) using transcriptome data). Another possibility could be that the single-gene phylogenetic approach generally results in a low resolution and topological incongruence among the trees (Walden and Schranz, 2022).

As one way to get additional support for the placement of the WGD/WGT events in Cleomaceae, we employed a modified species-tree reconstruction approach based on a total of 52 gene families with 1 *Cv* : 2 *Gg* : 3 *Th* syntenic relationship and located on four ancestral syntenic blocks (Supplemental Data Set 1). By selecting for syntenic blocks, we could make use of the shared evolutionary history of collinear genes and increase phylogenetic resolution compared to the single-gene approach. Also, to analyze the relationship among the subgenomes, we split the 104 *G. gynandra* and 156 *T. hassleriana* gene family members that are syntenic to the 52 *C. violacea* genes according to their subgenome localization. The resulting ASTRAL tree (Figure 3D**)** showed a split between the most fractionated and less fractionated subgenomes of *T. hassleriana* and *G. gynandra* (i.e. *Gg*MF and *Th*MF_2_ vs. *Gg*LF, *Th*MF_1_, and *Th*LF). This is in line with a WGD event before the split of the two lineages and followed by the biased subgenome gene fractionation. The *Th*LF subgenome likely resulted from a “+1×” addition to the *T. hassleriana* lineage. This is similar to the case of *Brassica* plants in which the more recently added subgenome is the least fractionated genome equivalent (Cheng et al., 2014). Our species-tree approach resulted in high posterior probability (*pp ≥ 0.95*) and relatively high quartet scores at all nodes, though gene and site concordance were not as high (Figure 3D and Supplemental Table 10). Collectively, we hypothesized that the two species, *G. gynandra* and *T. hassleriana*, first shared a WGD event (*Gg-α,* 2x) and *T. hassleriana* further experienced a *Th-α* (+1×) event through hybridization. More recently, Mabry et al. (2020) showed that a similar *Ks* peak to that of *Gg-α/Th-α* was detected in several Cleomaceae species including *G. gynandra*, *T. hassleriana*, *Cleomaceae* sp., *M. giganteus*, and *S. monophyla.* Thus, the *Gg-α* event is likely shared by several nested clades within the Cleomaceae family including *Gynandropsis*, *Tarenaya*, *Melidiscus*, African clades, and probably also Andean, *Cleoserrata* and *Dactylaena*, clades (Patchell et al., 2014; Bayat et al., 2018; Mabry et al., 2020).

To further test this possibility, we performed gene-tree reconciliation of 9,465 orthogroups identified from 10 Brassicales species including six Cleomaceae and Brassicaceae mentioned above (genomes available) and three other Cleomaceae species *Cleomaceae* sp., *M. giganteus*, and *S. monophyla* (transcriptomes available from Mabry et al. (2020)), plus *Carica papaya* as an outgroup (see “Methods” section and Supplemental Data Set 2 for details). Figure 3E shows a reconciled species-tree with nodes (N1–N8) and their corresponding gene duplications. As expected, elevated numbers of gene duplications were detected at nodes corresponding to the well-studied WGD/WGT events, including N1 (*Br-α*, 7,585), N2 (*At-α*, 2,664), and N8 (*At-β*, 2,383). Among the nodes shared by five Cleomaceae species that had a *Gg-α/Th-α*-like *Ks* peak reported in Mabry et al. (2020), N6 showed the highest number of gene duplications (3,602), likely corresponding to the *Gg-α* event. However, the correct placement of the *Th-α* event is still uncertain since we detected two nodes with high numbers of gene duplications (N3 and N5, 1,407 and 1,967, respectively). This could be due to differential fractionation rates among these species. Because the numbers of gene duplications for N3 and N5 were as high as 39%–55% of that of N6 (*Gg-α*), these could be attributed to the added third genome being the least fractionated. Nevertheless, the results strongly support the hypothesis that a series of sequential events including a WGD (2×) and hybridization (+1×) gave rise to the genome of *T. hassleriana*, similar to the cases of the hexaploid wheat (*Triticum aestivum*) (Mayer et al., 2014) or the Asteraceae family (Barker et al., 2016).

In light of the results presented in Figures 1C, 2, and 3, A–E and additional syntenic depth comparisons between *C. violacea* and two Brassicaceae species (showing 1:2 and 1:6 patterns to *A. thaliana* and *B. rapa,* respectively, Supplemental Figure 7), we propose a phylogenetic relationship between Brassicaceae and Cleomaceae families, and the polyploidy events that occurred in both lineages (Figure 3F). We included the six representative species for which genomes and syntenic information are available. These six species share the more ancient *At-β* WGD event. Then, after the separation of the two lineages, the progenitor of all three Brassicaceae species underwent the *At-α* WGD event and later the *Brassica* lineage underwent the *Br-α* WGT event. Among the Cleomaceae species, *G. gynandra* and *T. hassleriana* share the *Gg-α* WGD event, and the addition of a third genome (*Th-α*, +1×) took place in the *T. hassleriana* ancestor but not in the ancestor of *G. gynandra*. The younger *Ks* peak in *T. hassleriana* compared to that in *G. gynandra* likely reflects the additional genome that was added to it after the divergence of the two species following the *Gg-α* event. Collectively, this means that from one GE in the most recent common ancestor of these species, it is expected that there is one GE in *C. violacea*; two GEs in *A. arabicum*, *A. thaliana*, and *G. gynandra*; three GEs in *T. hassleriana*; and six GEs in *B. rapa* (Figure 3F).

### Different modes of gene duplication contributed to gene family expansions in *G. gynandra*

Both whole-genome and single-gene duplication provide opportunities for evolutionary change that could affect entire pathways and processes, and thereby give rise to novel traits through neo-/sub-functionalization (Monson, 2003; Hofberger et al., 2013; van den Bergh et al., 2014; Ren et al., 2018). WGD duplicated genes are those found within the syntenic regions of the same genome or between different genomes (i.e. originating from WGD/WGT events). Single-gene duplicates are a result of continuous processes within a genome (Lynch and Conery, 2000) and could be further classified into different modes including tandem, proximal, transposed, and dispersed duplicates (see “Methods” section for more information).

**Figure 4:**
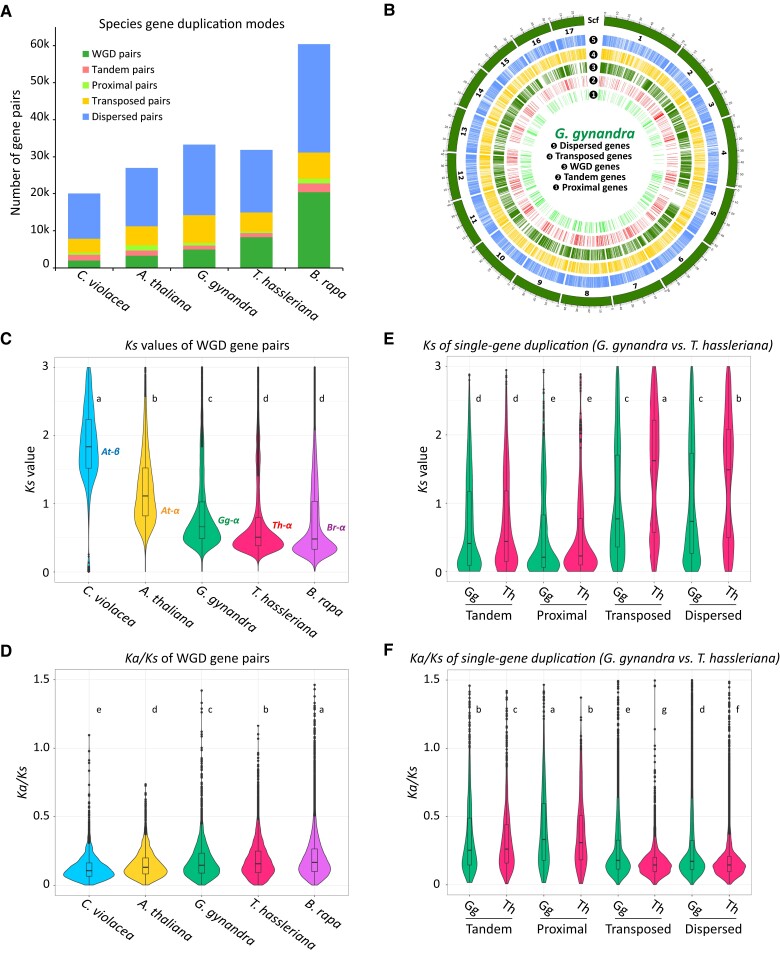
Different modes of gene duplication and evolutionary patterns of duplicated gene pairs in the *G. gynandra* and *T. hassleriana* genomes. A, Number of gene pairs originating from different modes of gene duplication in selected Brassicaceae and Cleomaceae genomes. Duplicated genes were identified within each genome using *Nelumbo nucifera* (the sacred lotus) as outgroup. For the number of genes identified for each mode of gene duplication, see Supplemental Figure 10. B, Distribution of duplicated genes of different modes in the *G. gynandra* genome. Only the 17 largest pseudomolecules are shown. Scaffold length is in Mb. C and D, Evolutionary patterns of WGD-derived gene pairs from *C. violacea*, *A. thaliana*, *G. gynandra*, *T. hassleriana* and *B. rapa* including *Ks* and *Ka/Ks* ratio distribution. E and F, Evolutionary patterns of single-gene-derived gene pairs from *G. gynandra* and *T. hassleriana* including *Ks* and *Ka/Ks* ratio distribution. Significance in (C–F) was based on one-way ANOVA (*Fisher’s LSD post hoc test*). Different letters indicate significant difference at *P* ≤ 0.05. Mean values are sorted alphabetically with “a” being the largest. For *Ka* plots, see Supplemental Figure S11.

We identified a total of 23,202 duplicated genes (∼75% of total genes) in the *G. gynandra* genome, representing these five modes of gene duplication that resulted in 33,297 gene pairs (Figure 4A, Supplemental Table 11 and Supplemental Figure 10). These duplicated genes were distributed across the 17 pseudomolecules and exhibited a higher density in the pseudomolecule arms than centromeres (Figure 4B). When compared with the results from other genomes in Cleomaceae and Brassicaceae, the numbers of duplicated gene pairs were as follows, *C. violacea:* 20,011 pairs; *A. thaliana:* 27,010 pairs; *T. hassleriana:* 31,882 pairs; and *B. rapa:* 60,419 pairs. When only WGD-derived gene pairs were considered, *A. thaliana* and *G. gynandra* had 1.6- and 2.5-fold, while *T. hassleriana* and *B. rapa* had 4.1- and 10.2-fold, respectively, of that in *C. violacea* (Figure 4A and Supplemental Table 11). The results are consistent with the previous reports for *A. thaliana*, *T. hassleriana*, and *B. rapa* (Qiao et al., 2019) and with the syntenic patterns between the three Cleomaceae species described earlier (Figure 2). The *Ks* distribution and *Ks* peaks of these WGD gene pairs identified in these species fell within the ranges that would be expected for each species, *At-β* in *C. violacea*, *At-α* in *A. thaliana*, *Gg-α* in *G. gynandra*, *Th-α* in *T. hassleriana*, and *Br-α* in *B. rapa* (Figure 4C, Supplemental Table 12 and Supplemental Data Set 3). The distribution of *Ka/Ks* (nonsynonymous-to-synonymous substitution ratio, representing selection pressure) of WGD gene pairs among the five genomes exhibited very similar profiles with relatively small values (i.e. the majority < 0.5 and the median < 0.25) (Figure 4D and Supplemental Figure 11). While *Ka/Ks* distributions are similar, the average and median *Ka/Ks* of these species could be sorted as follows: *C. violacea* < *A. thaliana* < *G. gynandra* < *T. hassleriana* < *B. rapa* (*P < 0.05*, one-way ANOVA *Fisher's LSD post hoc test*). In general, these data together with that in Qiao et al. (2019) suggest that WGD-derived genes show smaller *Ka/Ks* values compared to other duplication modes and are more conserved across these species.

We further compared the *Ks* and *Ka/Ks* distribution of other modes of gene duplication in the two Cleomaceae species, *G. gynandra* and *T. hassleriana* (*P < 0.05,* one-way ANOVA *Fisher's LSD post hoc test*). For each species, a distinct profile was found for each duplication mode, in which proximal gene pairs showed the youngest *Ks* peak, followed by tandem, WGD, then transposed and dispersed gene pairs. The transposed and dispersed gene pairs had clearly one older peak at *Ks* > 1 and one younger peak at *Ks* < 1 (Figure 4E). A similar observation was also reported in the analysis of the pear (*Pyrus bretschneideri*) genome (Qiao et al., 2018). Interestingly, *T. hassleriana* had a larger ancient *Ks* peak for both transposed and dispersed genes compared to that of *G. gynandra*. Among these, WGD gene pairs likely correspond to those derived from the more recent *Gg-α* WGD*/Th-α* WGT events, while tandem and proximal (displaying lower *Ks* values) are those originated from single-gene duplication following these polyploidy events. The double peaks in the *Ks* distributions of transposed and dispersed gene pairs likely reflect their ancestral and more recent origins. *Ka/Ks* distribution of different gene duplication modes revealed that proximal and tandem duplicates had the highest, while WGD duplicates generally were among the duplication modes that had the lowest *Ka/Ks* in both *G. gynandra* and *T. hassleriana* genomes (Figure 4F, Supplemental Table 13 and Supplemental Data Set 3). Particularly, the proximal-derived gene pairs had the lowest *Ks*; however, they had the highest *Ka/Ks* compared to duplicated gene pairs from other modes in both species. The result is in line with a previous observation of 141 plant genomes (Qiao et al., 2019), which suggests that proximal and tandem duplicates might have a higher rate of evolution, and hence could be important in the acquisition of new traits (Maere et al., 2005; Qiao et al., 2019). Between the two species, *T. hassleriana* had a significantly higher *Ka/Ks* for WGD, but generally a lower *Ka/Ks* for other duplication modes compared to *G. gynandra.* It is noteworthy that, around 92%–100% of the duplicated gene pairs in each mode identified in *G. gynandra* and *T. hassleriana* showed a *Ka/Ks* ≤ 1. It would be tempting to conclude that most of these genes evolved under purifying selection pressure; however, Roth and Liberles (2006) and Wang et al. (2009b) argued that the cutoff of *Ka/Ks* = 1 is too stringent to infer selection pressure. A more reasonable approach would be to compare the *Ka/Ks* among the genomes or sets of genes to infer low and high selection pressures as in previous reports (Wang et al., 2009b; Huang et al., 2021). When a cutoff of *Ka/Ks* > 0.5 was considered, *T. hassleriana* had a slightly higher WGD gene pairs but fewer other duplication modes compared to that of *G. gynandra* (Supplemental Table 13). When a cutoff of *Ka/Ks* > 0.25 was considered, *T. hassleriana* had more WGD and tandem gene pairs but fewer of the rest compared with *G. gynandra*. Collectively, this highlights the different selection pressures the two genomes might have experienced.

### WGD and transposed gene duplication are associated with photosynthesis pathways in *G. gynandra*

**Figure 5:**
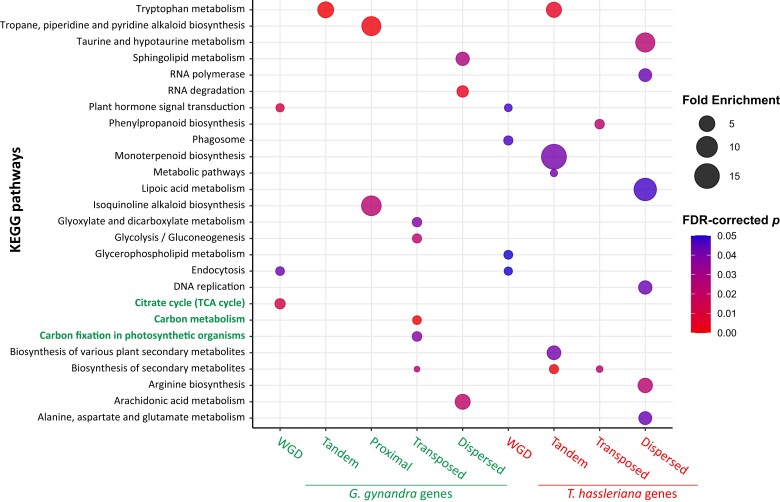
KEGG metabolic pathway enrichment analysis of duplicated gene sets from different duplication modes in *G. gynandra* and *T. hassleriana*. The analysis was performed using DAVID bioinformatics resources (Huang et al., 2009). Enriched pathways related to photosynthesis in the *G. gynandra* WGD and transposed duplicated genes including “*citrate cycle (TCA cycle)*,” “*carbon metabolism*,” and “*carbon fixation in photosynthetic organisms*” are in bold green font. For visualization, enriched pathways (*FDR*-corrected *P ≤ 0.05*) of each duplication mode are shown. For all pathways of *P ≤ 0.05* and *FDR ≤ 0.3*, see Supplemental Data Set 3.

Because different modes of gene duplication in the *G. gynandra* and *T. hassleriana* genomes were likely subjected to differential selection pressures, we asked if there are differentially enriched functions associated with them. Therefore, KEGG pathway enrichment analysis was performed for each gene set using DAVID tools (Huang et al., 2009). It is notable that the *G. gynandra* genome possesses less WGD but more tandem/proximal, transposed, dispersed genes and total gene counts compared to the *T. hassleriana* genome (Supplemental Figure 10). We detected a total 26 enriched KEGG pathways using a false discovery rate (*FDR)*-corrected *P*-value ≤ 0.05 for all duplication modes in both *G. gynandra* and *T. hassleriana* (Figure 5 and Supplemental Data Set 3). Due to a low number of genes, there was no enriched pathway detected in the *Th*-proximal gene set. Interestingly, three pathways that associated with photosynthesis including “*citrate cycle (TCA cycle)*,” “*carbon metabolism*,” and “*carbon fixation in photosynthetic organisms*” were found to be enriched only in WGD and transposed duplicated genes of the *G. gynandra* genome. As mentioned earlier, WGD genes are those within the syntenic regions including ancestral copies or those derived from WGD events, whereas transposed genes are nonancestral/non-WGD copies that resulted from single-gene duplication that copied a gene from an ancestral/WGD locus to a novel locus through a DNA- or RNA-based mechanism (Cusack and Wolfe, 2007). In our previous results (Figure 4, E and F), while transposed gene pairs from both species exhibited a double-peak *Ks* distribution, *G. gynandra* had more gene pairs in the lower *Ks* peak (*Ks* < 1), and a higher *Ka/Ks* than *T. hassleriana* (*P < 0.05*, one-way ANOVA *Fisher's LSD test*). This indicates that *G. gynandra* possesses more transposed genes that were derived from single-gene duplication following the more recent WGD events than *T. hassleriana*. Overall, the results suggest that the recent WGD and transposed gene duplication are likely the main modes that contributed to the expansion of genes related to photosynthesis in *G. gynandra*. It could be that these duplication modes provided additional gene copies besides the ancestral copies when the plants were still in the C_3_ state, which enabled selection and recruitment into the C_4_ pathway as suggested in previous studies (Monson, 2003; Williams et al., 2012; Ren et al., 2018).

### The impact of gene retention and gene duplication on the evolution of C_4_ photosynthesis in Cleomaceae

The evolution of C_4_ photosynthesis in *G. gynandra* is thought to have involved gene duplication and differential retention rates compared to its closest C_3_ relative *T. hassleriana*, which underwent a similar evolutionary trajectory but did not evolve to become a C_4_ plant (Bayat et al., 2018). It is important to note here that, in light of our findings, *T. hassleriana* likely possesses an extra genome compared to *G. gynandra*, the comparison between the two genomes is still relevant, since they shared previous duplication rounds including the *At-β* and *Gg-α* events. Additionally, our previous fractionation bias analysis also highlighted that the three *Th*-subgenomes exhibited a higher gene loss rate in comparison to the *Gg*-subgenomes.

**Figure 6:**
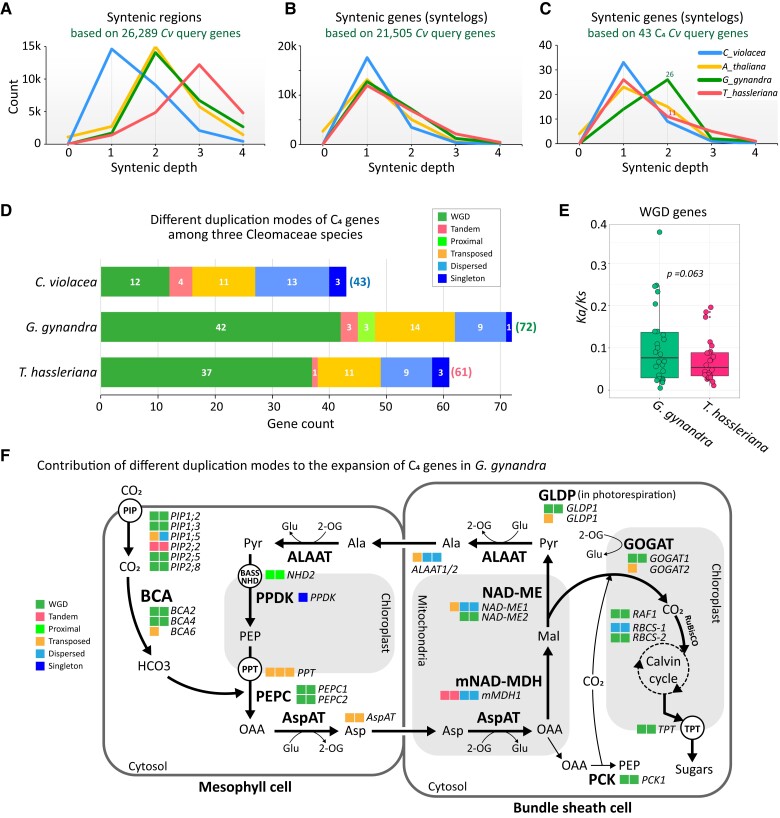
Contribution of gene duplication on the evolution of C_4_ photosynthesis in Cleomaceae. A–C, Plots of syntenic regions and genes analyzed by SynFind using all *C. violacea* genes and a set of 43 genes known to be involved in C_4_ photosynthesis, searched against the genomes of *A. thaliana*, *G. gynandra*, and *T. hassleriana*. For each panel, genes that showed no syntenic regions or syntelogs in both *G. gynandra* and *T. hassleriana* were excluded. D, Number of syntelogs identified in each species corresponding to the 43 C_4_ genes in the *C. violacea* genome. Modes of gene duplication were obtained from the Dupgene_finder results presented in Figure 4. E, *Ka/Ks* ratios of WGD-derived gene pairs among the syntelogs identified in *G. gynandra* and *T. hassleriana* as shown in (D). The *P*-value was calculated by Student's two-sided *t*-test. F, Expansion pattern and duplication modes of gene families involved in C_4_ photosynthesis. Each box represents one gene copy. Box colors indicate duplication mode that are shown at the bottom left corner. The expanded gene copies were derived from both SynFind analysis and Dupgene_finder results (in Figure 4). *BCA* (*BETA CARBONIC ANHYDRASE*), *PEPC* (*PHOSPHOENOLPYRUVATE CARBOXYLASE), AspAT (ASPARTATE AMINOTRANSFERASE), mMDH (MITOCHONDRIAL MDH)*, *NAD-ME (NAD-DEPENDENT MALIC ENZYME), ALAAT (ALANINE AMINOTRANSFERASE), PPDK (PYRUVATE ORTHOPHOSPHATE DIKINASE), PCK (PHOSPHOENOLPYRUVATE CARBOXYKINASE), GOGAT (GLUTAMINE OXOGLUTARATE AMINOTRANSFERASE), GLDP (GLYCINE DECARBOXYLASE P-PROTEIN), TPT (TRIOSE-PHOSPHATE⁄PHOSPHATE TRANSLOCATOR), PIPs (PLASMA MEMBRANE INTRINSIC PROTEIN), RBCS (RUBISCO SMALL SUBUNIT), RAF1 (RUBISCO ACCUMULATION FACTOR1),* OAA (OXALOACETATE), Asp (ASPARTIC ACID), Mal (MALATE), Pyr (PYRUVATE), Ala (ALANINE), PEP (PHOSPHOENOLPYRUVATE), Glu (GLUTAMATE), and 2-OG (2-OXOGLUTARATE).

Because the genome sequences are now available for both species, we further asked if there is a differential retention rate of genes involved in C_4_ photosynthesis between the two species, and if different gene duplication modes contribute to the expansion of C_4_-related gene families. To this end, we employed the SynFind algorithm (Tang et al., 2015) to analyze syntenic gene copy number across *C. violacea*, *A. thaliana*, *G. gynandra*, and *T. hassleriana* (as target genomes) using *C. violacea* genes as query reference. This allowed us to account for all target syntenic regions (with or without target genes present but upstream/downstream gene order conserved in relation to the reference) that were detected across the four genomes. Our results found that, when all syntenic regions corresponding to 26,289 *C. violacea* query genes were considered, the syntenic depth peaked at 1, 2, 2, and 3 for the *C. violacea, A. thaliana, G. gynandra*, and *T. hassleriana* genomes, respectively (Figure 6A and Supplemental Table 14). This observation is consistent with the syntenic patterns for *C. violacea, G. gynandra*, and *T. hassleriana* that are shown in Figure 2. When we considered only syntenic genes (present in the syntenic regions, termed “syntelogs”) that corresponded to 21,505 *C. violacea* query genes, *G. gynandra* and *T. hassleriana* exhibited very similar syntenic gene copy numbers (Figure 6B and Supplemental Table 14). Given that the *T. hassleriana* genome is composed of three genomic equivalents while that of *G. gynandra* consists of only two genomic equivalents compared with *C. violacea*, the results again reflect a higher fractionation rate in the *T. hassleriana* genome compared to the *G. gynandra* genome. Surprisingly, when we looked further into a group of 43 *C. violacea* genes from gene families that are known to encode key enzymes and transporters involved in the C_4_ biochemical reactions between M and BS cells in *G. gynandra* (van den Bergh et al., 2014; Rao and Dixon, 2016; Huang et al., 2021), an altered distribution was observed in the *G. gynandra* genome (Figure 6C and Supplemental Data Set 4). Out of 43 C_4_ reference genes, 29 *G. gynandra* genes (∼67%) retained at least two syntenic copies, while for *T. hassleriana* only 17 (∼40%) and 6 (∼14%) retained at least two and three syntenic copies, respectively. This resulted in a total of 72 nonredundant expanded gene copies in *G. gynandra* and 61 in *T. hassleriana* (Figure 6D). To rule out the possibility that this observation was due to chance, we performed 1,000 random samplings of 43 *C. violacea* genes each and compared the gene copy ratio found in the *G. gynandra* and *T. hassleriana* genomes to that of the 43 C_4_ photosynthesis-related genes (Supplemental Data Set 4). The results indicate that there is only a 0.3% probability that the observation could happen by chance, and, therefore, it is likely that *G. gynandra* preferentially retained more copies of C_4_ genes than *T. hassleriana*.

Among the total of 72 *G. gynandra* gene copies that are syntenic to 43 *C. violacea* C_4_ genes, 58.3% were derived from WGD, while 19.4%, 12.5%, 4.2%, and 4.2% were involved in transposed, dispersed, proximal, and tandem duplications, respectively (Figure 6D and Supplemental Data Set 4). Thus, the expansion and evolution of C_4_ genes in *G. gynandra* involved both WGD and single-gene duplication with WGD and transposed duplication being the major contributing modes. The heterogeneous origins of these C_4_ genes resulting from different modes of gene duplication might also mean that there was a long evolutionary transition from C_3_ to C_4_ photosynthesis in Cleomaceae, similar to the case in grasses (Wang et al., 2009b). Even though the *Ka/Ks* of most genes was below 1, in general, *G. gynandra* genes showed more genes of higher *Ka/Ks* compared to that of *T. hassleriana* (Figure 6E). Among these, *BCA2, BCA4, pMDH2 (PEROXISOMAL NAD-MALATE DEHYDROGENASE2), MDH,* and *NAD-ME2 (NAD-DEPENDENT MALIC ENZYME2)* showed higher *Ka/Ks* ratios in *G. gynandra.* Closer investigation of the key enzymes and transporters proposed to be important for the NAD-ME subtype of C_4_ photosynthesis used by *G. gynandra* revealed that most of the gene families had expanded compared to those in *C. violacea* (Figure 6F). Among these, the expansion of several gene families was attributed to the WGD, including *BCA2, BCA4, PEPC* (*PHOSPHOENOLPYRUVATE CARBOXYLASE), NAD-ME2, GOGAT1 (GLUTAMINE OXOGLUTARATE AMINOTRANSFERASE), GLDP1 (GLYCINE DECARBOXYLASE P-PROTEIN1), RBCS-2 (RUBISCO SMALL SUBUNIT2), RAF1* (*RUBISCO ACCUMULATION FACTOR1*), *PCK* (*PHOSPHOENOLPYRUVATE CARBOXYKINASE*), *TPT (TRIOSE-PHOSPHATE⁄PHOSPHATE TRANSLOCATOR),* and *PIPs (PLASMA MEMBRANE INTRINSIC PROTEIN)* (Supplemental Data Set 4). By contrast, transposed and dispersed duplication contributed to the expansion of *AspAT (ASPARTATE AMINOTRANSFERASE), mMDH1 (MITOCHONDRIAL MDH1)*, *ALAAT1/2 (ALANINE AMINOTRANSFERASE* 1/2), *PIPs, RBCS-1,* and *PPT (PHOSPHATE/PHOSPHOENOLPYRUVATE TRANSLOCATOR)*. Tandem and proximal duplication also contributed to the expansion of *mMDH1, NHD2 (SODIUM:HYDROGEN ANTIPORTER2),* and *PIPs* genes, respectively. Taken together, the results suggest that both WGD and single-gene duplication likely contributed to the expansion of C_4_ genes in the C_4_ plant *G. gynandra*. In so doing, this could have provided duplicated gene copies allowing the evolution of C_4_ pathways through preferential retention and recruitment of these genes.

### Comparative tissue/organ- and cell type-specific gene expression analysis uncovered subgenome dominance and the upregulation of functional C_4_ duplicated gene copies

Our results revealed that subgenome fractionation bias occurred in both *G. gynandra* and *T. hassleriana* following their shared allotetraploid *Gg-α* WGD event, and also after the interspecific hybridization (*Th-α*) in *T. hassleriana*. We then asked if these subgenomes also exhibit a gene expression dominance pattern as observed in other species, for example, *Arabidopsis* allotetraploids (Wang et al., 2006), *Brassica* plants (Liu et al., 2014), and cotton (*Gossypium hirsutum*) (Senchina et al., 2003). We utilized a total of 16 paired tissue-specific transcriptome datasets of the key tissue/organs including leaf, stem, root, seed, flowers, and seedlings from the two species previously reported in Külahoglu et al. (2014). To also analyze spatial gene expression, we included the cell type-specific transcriptome data from Aubry et al. (2014) that were derived from *G. gynandra* mesophyll (M) and bundle sheath (BS) cells isolated by laser capture microdissection (LCM) (Supplemental Data Set 5).

**Figure 7.**
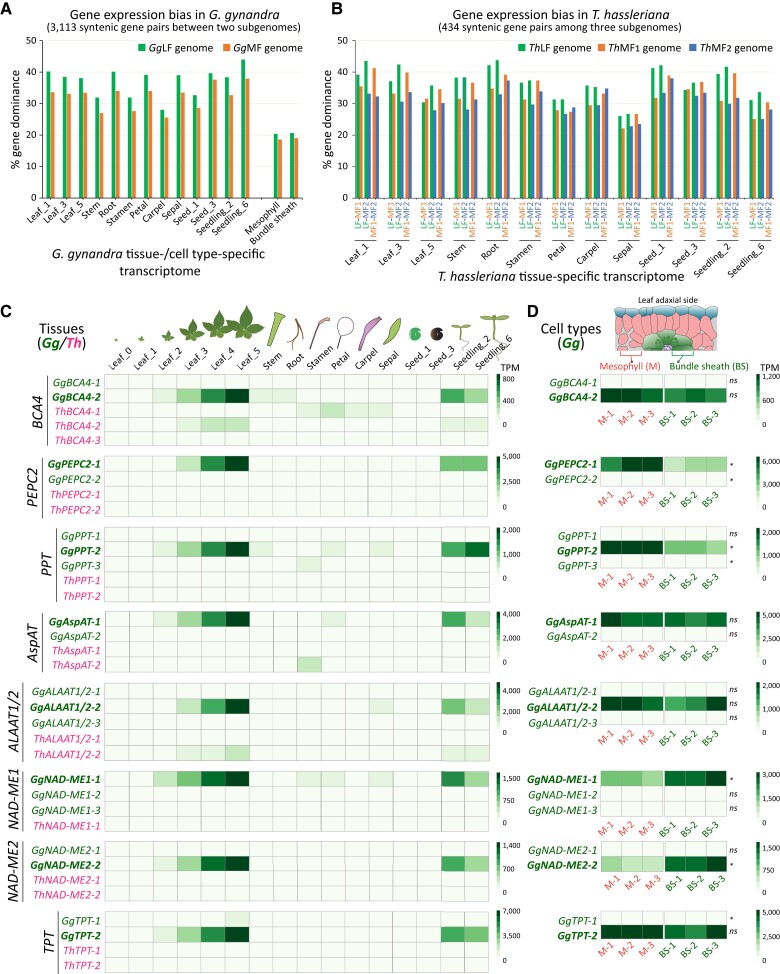
Tissue- and cell type-specific gene expression analysis of subgenomes and genes from the C_4_ photosynthesis pathway. A, Subgenome gene expression bias in the *G. gynandra* genome based on a total 3,113 syntenic ohnologous gene pairs between the two subgenomes. Tissue- and cell type-specific transcriptome data were used to calculate gene expression bias. For each sample, gene expression was compared between each syntenic gene pairs. B, Subgenome gene expression bias in the *T. hassleriana* genome based on 434 syntenic ohnologous gene triads across the three *Th*-subgenomes. Only tissue-specific transcriptome data were used. For (A and B), paired tissue-specific data were derived from the same study (Külahoglu et al., 2014). Gene dominance between each syntenic ohnologous gene pair was determined by Student's two-sided *t*-test, *P ≤ 0.05, n* = 3. C, Tissue-specific gene expression analysis of key gene families in the C_4_ pathway, showing a high expression level of one *Gg* duplicated gene copy compared to other copies found in two species for each gene family. These gene copies with elevated gene expression were also expressed highly in the cell type-specific transcriptome data (D) and displayed a preferential expression pattern in mesophyll or bundle sheath cells, or equally high expression in both samples for those localized in both cell types. A subset of 13 samples was used in (A) and (B), while a subset of 16 samples was included in (C), from the total of 18 original samples (Külahoglu et al., 2014). For (A–C), mean values of three replicates were used per sample. For (D**)**, data from three replicates are presented. Significance was calculated for means between two cell types for each gene using Student's two-sided *t*-test. Ns: nonsignificant. *Significant at *P* ≤ 0.05. For other C_4_ genes, see Supplemental Data Set 5.

For *G. gynandra*, gene expression (TPM) between 3,113 syntenic ohnologous gene pairs from its two subgenomes was compared using the “horserace experiment” approach (Cheng et al., 2012), and then the percentage of genes showing a higher gene expression (Student's two-sided t-test, *P ≤ 0.05, n* = 3) for each subgenome was calculated. Our analysis revealed that ∼40%–82% of the 3,113 *G. gynandra* syntenic gene pairs were differentially expressed in the transcriptome data (Figure 7A and Supplemental Data Set 5). A lower percentage of gene dominance was observed in the cell type-specific compared to the tissue-specific data; however, in all tissues and cell type samples, the *Gg*LF subgenome showed more gene expression dominance compared to the *Gg*MF. For *T. hassleriana*, due to a higher fractionation rate in the *Th*MF_2_ subgenome, we focused on 434 syntenic ohnologous gene triads found across the three *Th*-subgenomes. Applying the same approach as for *G. gynandra*, we found that ∼48%–77% of genes were differentially expressed between subgenomes (Student's two-sided t-test, *P ≤ 0.05, n* = 3, Figure 7B and Supplemental Data Set 5). In most of the tissue-specific samples, more dominant genes were found in the *Th*LF subgenome, followed by the *Th*MF_1_ and *Th*MF_2_. This result suggests that both the *Gg*LF and *Th*LF subgenomes, beside showing a higher gene density, also exhibit a higher level of gene expression compared to the MF subgenomes in the respective species. The results further support the hypothesis that the *Th*LF subgenome is the one added to the *T. hassleriana* genome at *Th-α* event, since it retained more genes and is dominant among the three *Th*-subgenomes. Furthermore, the biased fractionation and gene expression observed in *Gg*LF vs. *Gg*MF and *Th*MF_1_ vs. *Th*MF_2_ pairwise subgenome comparisons further support the notion that an allotetraploid event was shared between the two species prior to the addition of the third subgenome in *T. hassleriana*.

Finally, to illustrate the expression pattern of C_4_ duplicated gene copies in both *G. gynandra* and *T. hassleriana*, we analyzed the previously identified copies in Figure 6F using both tissue- and cell type-specific transcriptome data (Supplemental Data Set 5). The results highlight that among the duplicated gene copies retained in each gene family after the recent WGD/WDT event, one of the *G. gynandra* copies (in bold green) was highly expressed compared to other copies in both *G. gynandra* and *T. hassleriana* (Figure 7C). In general, these genes were expressed at a higher level in photosynthetic samples (i.e. leaf and seedling) than in nonphotosynthetic samples (i.e. stem, root, flowers and seed). Their expression was increased during leaf development (Leaf_0 to Leaf_5) and peaked at the “Leaf_5” stage. The same gene copies were also found to be highly expressed in the *G. gynandra* cell type-specific data (Figure 7D), and generally showed a preferential expression pattern according to their function and expected protein localization, as depicted in Figure 6F. For example, the expression of *BCA4*, *PEPC2*, and *PPT* was high in M cells and that of *NAD-ME1* and *2* was high in BS cells, while that of *AspAT* and *ALAAT1/2* was high in both cell types. Collectively, our results showed that while most of the C_4_ gene families were expanded in *G. gynandra* through either WGD or single-gene duplication, only one of the duplicated copies in each family was recruited into the C_4_ pathway. These likely functional C_4_ gene copies displayed an elevated expression level in photosynthetic tissues and were expressed in a cell type-specific manner.

## Discussion

Whole-genome assembly, especially of orphan crops, can provide new perspectives on genome evolution, trait genetics, and genic information. This, in turn, could be applied to develop modern and efficient breeding programs by enhancing the use of technologies such as genomic selection (Budhlakoti et al., 2022) or targeted mutagenesis (Belhaj et al., 2013). In this study, we present the genome sequence of the C_4_ plant *G. gynandra*, an economically important leafy vegetable and medicinal plant. We then employed the newly generated genome sequence in a series of comprehensive analyses to determine the history of WGD/WGT in the Cleomaceae family; subgenome dominance and biased fractionation; and the impact of different gene duplication modes on the expansion, evolution, and gene expression patterns of *G. gynandra* gene families, focusing on those involved in the C_4_ photosynthesis pathway.

Our final *G. gynandra* genome assembly (v3.0) is 740 Mb, with ∼99% of the assembly anchored onto 17 pseudomolecules. It has an N50 of 41.9 Mb, a BUSCO completeness score of 98.2%, and 30,933 well-supported gene models (33,748 transcripts). The genome also contains a substantial number of repetitive elements, which accounts for ∼69% of its size. The availability of the genome sequences of *G. gynandra* and its relatives (*C. violacea* and *T. hassleriana*) provides an excellent opportunity to study genome evolution and gene families involved in the evolution of C_4_ photosynthesis in Cleomaceae. Moreover, our results confirmed that the genomes of *G. gynandra* and its close relatives display a high level of synteny and collinearity with other genomes from Brassicaceae including *A. thaliana* and *B. rapa*, as suggested in previous studies (Schranz and Mitchell-Olds, 2006; Cheng et al., 2013). The Brassicaceae contains only species with C_3_ and C_3_–C_4_ intermediate photosynthesis, but not C_4_. The close evolutionary proximity of *G. gynandra* and the model plant Arabidopsis for which there are significant genetic resources facilitates comparative functional and evolutional analyses, and positions *G. gynandra* as a model for the genomic analysis of C_4_ photosynthesis in the Brassicales.

Within the Cleomaceae family, evidence of an ancient WGT event (*Th-α*) was previously found in *T. hassleriana* (Cheng et al., 2013), a closely related species of *G. gynandra*. This triplication event was independent of the Brassicaceae-specific duplication (*At-α*) and the nested *Brassica* triplication (*Br-α*) (Schranz and Mitchell-Olds, 2006; Cheng et al., 2013; Mabry et al., 2020). In the absence of multiple key genome sequences for Cleomaceae species, it was impossible to adequately place the Cleomaceae-specific polyploidy event. However, using transcriptome data, Mabry et al. (2020) suggested that the *Th-α*-like polyploidy event is shared by species of several nested clades within the Cleomaceae family including *Gynandropsis*, *Tarenaya*, *Melidiscus*, and African clades; and likely also Andean, *Cleoserrata* and *Dactylaena* (Patchell et al., 2014; Bayat et al., 2018). Our interspecies genome synteny analysis of three Cleomaceae species, *C. violacea*, *G. gynandra*, and *T. hassleriana*, revealed that the *Th-α* triplication is not present in *C. violacea* and appears as a duplication event in *G. gynandra* (which we refer to as *Gg-α*). Among the detected syntenic regions, the three Cleomaceae genomes exhibit a clear pattern of 1:2:3 syntenic relationship for *C. violacea, G. gynandra*, and *T. hassleriana*, respectively. Using a combined approach of synteny, phylogenetics and gene duplication dating, we showed that both *G. gynandra* and *T. hassleriana* first underwent the common *Gg-α* WGD event but then *T. hassleriana* subsequently acquired an additional genome equivalent, likely through hybridization. Our analyses of subgenome fractionation and expression bias support the notion that this *Gg-α* WGD was an allotetraploid event, and that the added subgenome to *T. hassleriana* is likely the least fractionated one, namely *Th*LF. By integrating genome data with the available transcriptome data from Mabry et al. (2020), we provided further evidence that the *Gg-α* event is likely shared with several species within the Cleomaceae family. As new genome sequences become available for the Cleomaceae, it will be possible to further clarify the evolutionary history of the family.

One intriguing question relates to the quantitative importance of WGD and single-gene duplication to the evolution of C_4_ photosynthesis from C_3_ photosynthesis in Cleomaceae. This could provide an improved understanding of processes associated with the evolution of C_4_ photosynthesis in *G. gynandra* compared with *T. hassleriana*, even though the two species underwent the same WGD event (*Gg-α*). The contribution of different gene duplication modes including WGD and single-gene duplication to the evolution C_4_ photosynthesis was proposed first by Monson (2003). In this process, gene duplication provides duplicated gene copies as prerequisite materials when the plants were still in the C_3_ state for selection and recruitment into C_4_ photosynthesis. As a result, one of those duplicated gene copies could become highly expressed in a more organ-, cell type-, or organelle-specific manner (Monson, 2003). It appears that modifications in sequence to generate these alterations in expression are diverse and can include modifications to gene promoters (Brown et al., 2011; Williams et al., 2016) or coding regions (Reyna-Llorens et al., 2018). Upregulation of one gene copy has been shown to take place in *G. gynandra* compared with *T. hassleriana* based on transcriptome data (Külahoglu et al., 2014; van den Bergh et al., 2014; Huang et al., 2021). The transition from C_3_ to C_4_ photosynthesis could in fact have involved genes that are related to a series of events and changes including those related to plant physiology, biochemistry, and anatomy (Sage, 2004; Gowik and Westhoff, 2010). In this study, as an exemplary case, we systematically investigated gene families encoding key enzymes and transporters that facilitate the C_4_ biochemical reactions between M and BS cells in the NAD-ME subtype of *G. gynandra*. Our results suggest that the *G. gynandra* genome likely preferentially retained more copies of these specific C_4_ gene families following the WGD event compared with *T. hassleriana*. We also confirmed that both WGD and single-gene duplication (especially transposed duplication) were involved in the expansion of these C_4_ gene families. The involvement of different modes of gene duplication in this process might mean that, similar to the case of C_4_ grasses (Wang et al., 2009b), there was also a long transition from C_3_ to C_4_ photosynthesis after the WGD event in Cleomaceae. Finally, by integrating the tissue- and cell type-specific transcriptome data previously published for *G. gynandra* and *T. hassleriana* (Aubry et al., 2014; Külahoglu et al., 2014), we illustrated the expression patterns of the C_4_ duplicated genes and identified the likely functional gene copies among the expanded copies from different gene duplication modes.

### Conclusions

In conclusion, the genome sequence of *G. gynandra* presented in this study provides a deeper understanding of the polyploidy history of the Cleomaceae and sheds light on the possible scenarios of step-wise ancient polyploidy events of *T. hassleriana* and *G. gynandra*. The genome of *G. gynandra* underwent a WGD event (*Gg-α*) after the divergence of Cleomaceae from Brassicaceae, which is also likely shared with several nested clades within the Cleomaceae family. Comprehensive analysis of gene families involved in the C_4_ photosynthesis suggested that compared to its C_3_ close relative *T. hassleriana*, *G. gynandra* preferentially retained more copies of these genes. Both whole-genome and single-gene duplication were found to be responsible for the expansion of C_4_ gene families in *G. gynandra*. We anticipate that our data will enhance the understanding of the impact of gene duplication and gene retention on the evolution of C_4_ photosynthesis in Cleomaceae.

## Materials and methods

### Library construction, sequencing, and genome assembly of *G. gynandra*

Plant materials of the reference line “GYN” originating from Malaysia were provided by the World Vegetable Center. Seeds were sown and inbred by hand-pollination for four generations in a greenhouse at Wageningen University. Leaf tissues were collected and used for high-molecular-weight (HMW) genomic DNA extraction according to the CTAB method (Clarke, 2009). Genome sequencing was done by employing a combined approach of Illumina sequencing, 10X Genomics sequencing and chromatin conformation capture Hi-C technologies. For Illumina sequencing, we constructed 8 different insert-size paired-end (PE) libraries of 250 bp, 350 bp, 500 bp, 800 bp, 2 kb, 5 kb, 10 kb, and 20 kb. The libraries were prepared and sequenced by BGI company (Shenzhen, China) with a HiSeq 2000 instrument to obtain a total of ∼209.6 Gb raw PE read data. To prepare the raw read data for genome de novo assembly, low-quality reads, adapter sequences, and duplicated reads were removed, and high quality reads were used for genome assembly by SOAPdenovo software v2.21 (Li et al., 2010), with a k-mer of 63. The output contigs were subsequently assembled into scaffolds by SSPACE software v2.0 (Boetzer et al., 2010) to generate the first draft version (v1.0) of the *G. gynandra* genome.

For 10× Genomics sequencing, HMW genomic DNA extraction, sample indexing, and barcoded libraries preparation were performed by 10× Genomics (Pleasanton, CA, USA) according to the Chromium Genome User Guide and as published elsewhere (Weisenfeld et al., 2017). The libraries were sequenced with Illumina HiSeq 2,500 with 125 bp PE reads and the raw reads were assembled using the 10X Genomics Supernova software v1.0 (Weisenfeld et al., 2017). For scaffolding of the draft genome, ARCS v1.1 (Yeo et al., 2017) was used to add barcodes to read identifiers, map linked reads against the reference genome, use the barcode information to find the reads linking contigs and assemble them in scaffolds. The resulting genome assembly is referred to as the second version (v2.0).

Finally, an additional Hi-C library was prepared and sequenced by Dovetail Genomics (Scotts Valley, CA, USA), and employed for another round of scaffolding using the 3D-DNA pipeline (v180922, https://github.com/theaidenlab/3d-dna) to obtain the final genome assembly (v3.0).

### Estimation of genome size based on read data k-mer distribution

Due to a high repetitive content (Beric et al., 2021), the genome size of *G. gynandra* was estimated with values of k ranging from 21 to 121. KmerGenie v1.7051 (Chikhi and Medvedev, 2013) and GenomeScope v2.0 (Vurture et al., 2017) both suggested the best k-mer being 99; therefore, a k-mer of 99 was used to estimate the genome size. For GenomeScope, the k-mer distribution was generated by KMC v3 (Kokot et al., 2017).

### Identification of repetitive elements and genes prediction

Repeats and transposable elements in the genome were masked with RepeatModeler v2.0/RepeatMasker v4.1.2 and RepeatProteinMask (Tarailo-Graovac and Chen, 2009). Firstly, the ab initio prediction program RepeatModeler (v2.0.3) was employed to build the de novo repeat library based on the genome, and then contamination and multicopy genes in the library were removed. Using a custom library that consisted of de novo identified repeats, and the Dfam v3.3 and RepBaseRepeatMaskerEdition-20181026 databases, RepeatMasker was run to identfy homolog repeats in the genome and classify them. Three approaches were used for gene prediction: (1) homology search with closely related species including *A. thaliana, A. lyrata, B. rapa, Thellungiella parvula*, and *T. hassleriana*; (2) de novo prediction using AUGUSTUS v3.1.0 (Stanke and Morgenstern, 2005), SNAP v20131129 (Korf, 2004), and GlimmerHMM v3.0.4 (Majoros et al., 2004); and (3) evidence-based annotation using transcriptomes from 18 tissue-specific transcriptome data previously generated for *G. gynandra* (Külahoglu et al., 2014). These transcriptome data were derived from the major tissues/organs at different developmental stages including leaf, stem, root, seed, seedling, sepal, stamen, petal, and carpel (see Figure 7 and Supplemental Data Set 5, for subsets of 16 and 13 samples analyzed). We used the program GLEAN v1.0.1 (Elsik et al., 2007) to combine the predicted gene models to produce consensus gene sets. Initially, the annotation was done for the first draft genome (v1.0), then was carried over to the final assembly (v3.0) using flo v1.0.0 (same species annotation lift over pipeline—https://github.com/wurmlab/flo). This final annotated version of the genome was used in all subsequent analyses. The BUSCO v5.3.2 and plant-specific Embryophyta odb10 dataset (including 1,614 single-copy orthologs (Simão et al., 2015)) were used to assess the genome completeness.

### Gene functional annotation

The *G. gynandra* predicted protein sequences were compared with those in the Swiss-Prot release 2022_04 (O'Donovan et al., 2002) and TrEMBL release 2022_01 (O'Donovan et al., 2002) databases using Diamond BLASTP v2.0.14 (Buchfink et al., 2021) with the following settings “*-e 1e-5 -k 1.*” To predict protein function, InterProScan v5.55-88.0 (Zdobnov and Apweiler, 2001) was employed to compare *G. gynandra* protein sequences with those in several databases with the options*“-goterms*” to retrieve both protein domains and associated GO terms. To maximize the searching, we utilized all 17 databases supplied with InterProScan. KEGG mapping was done using BlastKOALA v2.2 (Kanehisa et al., 2016) with “*plants*” as taxonomy group and searched against the “*family_eukaryotes*” KEGG gene databases. Additionally, GO term enrichment of gene sets was carried out using WEGO v2.0 (Ye et al., 2018), while KEGG pathway enrichment was performed using DAVID bioinformatics resources v2021 (Huang et al., 2009) with all genes as the background.

### Orthogroup classification

Protein sequences from *A. thaliana* (27,654)*, B. rapa* (46,250)*, C. violacea* (21,850)*, G. gynandra* (30,933), and *T. hassleriana* (27,396) were used for orthogroup clustering by Orthofinder v2.5.4 (Emms and Kelly, 2019) with default settings. Only the longest protein variant sequences (as primary) representing genes retained by Orthofinder script *primary_transcript.py* were used for this analysis. The presence or absence of identified orthogroups was used to identify those that are commonly shared among species or specific to each species, and to the Brassicaceae or Cleomaceae families, respectively.

### Genome synteny and duplication analyses

Genome synteny and collinearity, dotplots and *Ks* values of the detected syntenic gene pairs were generated by SynMap tool (Lyons et al., 2008) on the CoGe v7 (Castillo et al., 2018). Syntenic gene pairs across species were analyzed by both MCscan v0.8 (Tang et al., 2008) implemented in python (https://github.com/tanghaibao/jcvi/wiki/MCscan-(Python-version)) and SynFind (Tang et al., 2015) on the CoGe. For MCscan analyses, the function “*jcvi.compara.catalog ortholog*” was used to search for syntenic regions within and between genomes. Then, “*jcvi.compara.synteny depth*” was run to calculate syntenic depth. Syntenic blocks of a minimum of four (for microsynteny) or 30 (for macrosynteny) colinear genes were identified using the function “*jcvi.compara.synteny screen.*” Macrosynteny and microsynteny, karyotype comparisons were visualized using the function “*jcvi.graphics.karyotype*”. For SynFind analyses, *C. violacea* genes were used as a query reference searched against the target genomes of *A. thaliana*, *B. rapa*, *C. violeaceae*, *G. gynandra* and *T. hassleriana*, with default parameters (i.e. comparison algorithm: Last, gene window size: 40, minimum number of genes: 4, scoring function: collinear, syntenic depth: unlimited). SynFind outputs syntenic gene pairs (syntelogs) if a match is found in the syntenic regions of the target genome and a “proxy for region” if the syntelog is missing in the target genome due to fractionation or translocation (Tang et al., 2015). In this case, since the syntelog of the query gene is missing, a proxy is determined by the neighboring gene pairs within the syntenic region, and the number of neighboring genes found is reflected by a synteny score. For each *C. violacea* query gene, we counted the total syntelogs + proxies (referred to as syntenic regions) and syntelogs only in each of the target genomes to infer their gene copy number status before and after fractionation following genome duplication, respectively. For each analysis, we excluded any genes that showed no syntenic regions or syntelogs in both *G. gynandra* and *T. hassleriana* (i.e. only found in *C. violacea* and/or other species).

### Subgenome fractionation bias analysis

To reconstruct and define the subgenomes of *G. gynandra* and *T. hassleriana*, we aligned each of these genomes to that of *C. violacea* (target/reference) using SynMap. This ran together with the FractBias program (Joyce et al., 2016) on CoGe v7 with default settings and the following modifications. The fractionation bias was calculated for syntenic genes in the target genome with a window size of 100 genes and a maximum of 70 scaffolds per genome. Syntenic depth was set based on the ploidy level for each genome pair, 1:2 for *C. violacea*: *G. gynandra* and 1:3 for *C. violacea*: *T. hassleriana.* Syntenic gene pairs between and across the subgenomes were also used for the subsequent subgenome phylogenetic and gene expression bias analyses.

### Phylogenetic analysis

To construct species tree, single-copy genes were identified by Orthofinder v2.5.4 across six species including *A. arabicum*, *A. thaliana, B. rapa, C. violacea, G. gynandra* and *T. hassleriana.* In this analysis, Orthofinder was run using the primary protein variant sequences from each species as described earlier, and with the option “*-M msa”* to infer maximum likelihood (ML) from multiple sequence alignment (Emms and Kelly, 2019). This used MAFFT v7.480 (Katoh et al., 2002) for sequence alignment and FastTree v2 (Price et al., 2009) for the phylogenetic tree inference. For gene trees, coding or protein sequences were aligned by MAFFT with the option the option “*G-INS-i*,” then poorly aligned regions were trimmed by trimAL v1.4.rev22 (Capella-Gutiérrez et al., 2009) with the option “*-automated1*.” The alignment files then were subjected to IQ-TREE v2.2.0 (Trifinopoulos et al., 2016) with default settings (1,000 bootstrap iterations) and MrBayes v3.2.7a (Ronquist et al., 2012) on CIPRES Science Gateway v3.3 (Miller et al., 2010) using the substitution model (GTR + gamma + I), MCMC chains running 10,000,000 generations and sampling tree every 1,000 generations for tree inferences using ML and Bayesian methods, respectively.

For ASTRAL tree, coding sequences of syntenic 1:2:3 genes in Cleomaceae (52 genes from four syntenic blocks in total) were aligned for each gene separately using MACSE v2.06 (Ranwez et al., 2018). These syntenic genes and blocks were derived from SynMap analysis (see “Subgenome fractionation bias analysis” section). The resulting alignments were subsequently curated using Gblocks v0.91b (Castresana, 2000) in codon mode with parameters “*-b5 = h and -b4 = 6”* (allowed gap positions half and minimum block length 6). ML gene trees were reconstructed using RAxML v8.2.12 (Stamatakis, 2014) with rapid bootstrapping followed by thorough ML search (*-f a*) using 1,000 bootstrap replicates and site heterogeneity model GTRGAMMA. We then reconstructed a species-tree with ASTRAL v5.7.8 (Zhang et al., 2018) and analyzed gene and site concordance factors with IQ-TREE v2.2.0 using 1,000 quartets for site concordance factors. Consensus trees were visualized in FigTree v1.4.3 (http://evomics.org/resources/software/molecular-evolution-software/figtree/). Sequence alignments and machine-readable phylogenetic trees are available in FigShare at https://doi.org/10.6084/m9.figshare.21505380.v1.

### Reconciliation of gene-tree and species-tree and mapping gene duplications

We used annotated proteins from 10 Brassicales species to cluster orthogroups by OrthoFinder v2.5.2 with default settings. These included six species with genome sequences available (described in “Orthogroup classification” section) and three other Cleomaceae species with transcriptome data available in Mabry et al. (2020): *Cleomaceae* sp., *M. giganteus*, and *S. monophyla*. The data from the *Carica papaya* genome (https://phytozome-next.jgi.doe.gov/info/Cpapaya_ASGPBv0_4) were included as outgroup species. Overall, 9,465 orthogroups with at least one protein sequence from each species were used to build gene trees. We made protein alignments using PASTA software v1.8.5 (Mirarab et al., 2015) for each orthogroup. IQ-TREE v1.6.1 was used to generate orthogroup phylogenies with 1,000 bootstraps. We performed gene-tree reconciliation using Notung v2.9.1.5 (Stolzer et al., 2012) with a model of gene duplication and loss without horizontal gene transfers. The gene trees were rerooted with an outgroup under the “*-reroot*” function in Notung. The 80% bootstrap value was used as a threshold to rearrange low support branches on gene trees based on the species-tree topology under the “*-rearrange*” function. The gene-tree reconciliation was performed using all orthogroup phylogenies. The cost of loss was set to 0.1 to account for missing data of transcriptomes (Koenen et al., 2020). Species tree topology was adapted from the ASTRAL-III coalescent-based species phylogeny (Mabry et al., 2020). Sequence alignments and machine-readable phylogenetic trees are available in FigShare at https://doi.org/10.6084/m9.figshare.21505380.v1.

### Identification of different modes of gene duplication in the *G. gynandra* genome

To study evolutionary consequences of gene duplication in the selected Brassicaceae and Cleomaceae species, we analyzed genome-wide gene duplication modes using DupGen_finder (accessed Jan 2022) (Qiao et al., 2019) with default parameters. The primary protein sequences and gff files of *A. thaliana, B. rapa, C. violacea, G. gynandra, T. hassleriana* (see BUSCO assessment in Supplemental Figure 12) were utilized, together with that of *Nelumbo nucifera* (the sacred lotus) as an outgroup. For each genome, we classified gene duplication into different modes including whole-genome duplication (WGD), tandem duplicates, proximal duplicates, transposed duplicates, and dispersed duplicates. This analysis was based on an all-versus-all local BLASTP v2.12.0 (*e-value = 1e−10*, top five matches) to find all potential homologous gene pairs within a given genome. Then, among the homologous gene pairs, WGD gene pairs were identified by the MCScanX algorithm (accessed Jan 2022) (Wang et al., 2012) within the syntenic regions of the same genome or between different genomes. Tandem gene pairs were those homologous genes that are adjacent to each other and located on the same chromosome, while proximal gene pairs were those homologous genes on the same chromosomes and separated by up to 10 genes. Transposed gene pairs were defined as non-WGD, nontandem, and nonproximal and consisted of one ancestral and one nonancestral copy. The ancestral gene copy could be those in WGD gene pairs (intraspecies) or within the syntenic regions between the target genome and outgroup genome (*N. nucifera,* interspecies). Dispersed gene pairs were those remaining gene pairs, while singletons were genes without any BLASTP hits.

### Estimation of *Ka*, *Ks*, and *Ka/Ks* ratios of duplicated gene pairs

To study evolutionary patterns, the *Ka* (the ratio of the number of substitutions per nonsynonymous site), *Ks* (the ratio of the number of substitutions per synonymous site), and *Ka/Ks* values were computed for all gene pairs of each mode of gene duplication by *KaKs*_Calculator v2.0 (Wang et al., 2010) following the pipeline in Qiao et al. (2019). Briefly, MAFFT was used to align each pair of gene sequences, then PAL2NAL v14 (Suyama et al., 2006) was used to obtain a codon alignment. The final alignment in AXT format was subjected to *KaKs*_Calculator to estimate *Ka*, *Ks*, and *Ka/Ks* based on the γ-MYN method (Wang et al., 2009a). To identify the *Ks* peaks corresponding to the WGD events, the *Ks* distribution of WGD gene pairs from each species was fitted by GMMs and the collinearity file generated by MCscanX, as described in Qiao et al. (2019). For identifying WGD events, only *Ks* ≤ 4.0 were included for these analyses, to avoid the saturated *Ks* values. For selection pressure analysis of duplicated genes derived from duplication modes, a *Ks* ≤ 3.0 cutoff was used.

### Expansion and contraction of gene families related to C_4_ photosynthesis in Cleomaceae

We investigated the evolution of a set of genes known to be involved in the C_4_ photosynthesis of the NAD-ME subtype that is found in *G. gynandra*. These genes encode key enzymes and transporters in the C_4_ cycle in the M and BS cells that were also included in the previous studies (van den Bergh et al., 2014; Rao and Dixon, 2016; Huang et al., 2021). To provide more insight into the evolutionary patterns of these genes, we analyzed the expansion and contraction of the selected C_4_ gene families among the three Cleomaceae species, *C. violacea, G. gynandra*, and *T. hassleriana*, using the *C. violacea* genes as reference query. The *A. thaliana* genome was also included in this analysis, to utilize the rich genetic information available for this species. The syntenic gene copy number and modes of gene duplication were obtained from SynFind and DupGen_finder analyses as described earlier.

### RNA-seq analysis of tissue- and cell type-specific gene expression

For gene expression analysis, we utilized two public datasets including tissue/organ-specific transcriptome atlases reported in Külahoglu et al. (2014) and cell type-specific transcriptome data from Aubry et al. (2014). The former contains paired data of key tissues/organs from the two species *G. gynandra* and *T. hassleriana*, while the latter were derived from *G. gynandra* mesophyll (M) and bundle sheath (BS) cells isolated by LCM. For each dataset, RNA-seq read quality before and after trimming was assessed by FastQC v0.11.9 (http://www.bioinformatics.babraham.ac.uk/projects/fastqc). Adapter sequences and low-quality reads were removed using Trimmomatic v0.39 (Bolger et al., 2014) with the following parameters: “ILLUMINACLIP: 2:20:10 SLIDINGWINDOW:4:15 LEADING:5 TRAILING:5 MINLEN:50.” To estimate transcript abundance, trimmed reads were aligned to the 30,933 *G. gynandra* or 27,396 *T. hassleriana* gene models using Bowtie2 (v2.4.5) (Langmead and Salzberg, 2012) with default settings. The mapping files were sorted by SAMTOOLS-1.16.1 (Li et al., 2009) and subjected to RSEM v1.3.3 (Li and Dewey, 2011) for transcript abundance quantification. The expression level was normalized as TPM. Three replicates were used for each sample.

### Other quantification analyses

Venn diagrams were generated using the online tools (http://bioinformatics.psb.ugent.be/webtools/Venn). Genome circular plots were drawn using Circos v0.69-9 (Krzywinski et al., 2009). Genome and gene set statistics were generated by QUAST v5.2.0 (Gurevich et al., 2013). All analyses in the Linux environment were conducted on local servers running Ubuntu 16.04.6 LTS hosted by the Biosystematics Group at Wageningen University, the Netherlands.

### Statistical analyses

All statistical analyses, unless otherwise stated, were carried out using Microsoft Excel and R program v4.0.2 with RStudio software v2022.07.2-576 (https://www.rstudio.com). The results of statistical analysis are provided in the respective Supplemental Tables and Data Sets.

## Accession numbers

Data supporting the findings in this work are available within the paper and in Supplemental Data. The final genome assembly (v3.0) and annotation of *G. gynandra* can be downloaded from CoGe via https://genomevolution.org/coge/GenomeInfo.pl?gid=58728 or FigShare at https://doi.org/10.6084/m9.figshare.21383760.v1. The *G. gynandra* genome assemblies (v2.0 and v1.0) and their annotations can be downloaded from https://doi.org/10.6084/m9.figshare.21383757.v1 and https://doi.org/10.6084/m9.figshare.21383754.v2, respectively. The *A. thaliana* araport11 genome data were downloaded from Phytozome 13 (https://phytozome-next.jgi.doe.gov/info/Athaliana_Araport11). The *A. arabicum* genome v3.1 data were downloaded from the *Ae. arabicum* DB (https://plantcode.online.uni-marburg.de/aetar_db/). The *B. rapa* genome v3.0 data were downloaded from the Brassicaceae Database (http://brassicadb.cn/#/). The *C. violacea* genome v2.1 data were obtained from Phytozome 13 (https://phytozome-next.jgi.doe.gov/info/Cviolacea_v2_1). The *T. hassleriana* genome v101 data were downloaded from NCBI accession number GCF_000463585.1 (https://www.ncbi.nlm.nih.gov/genome/annotation_euk/Tarenaya_hassleriana/101/). The *Nelumbo nucifera* genome data were downloaded from the Nelumbo genome database (http://nelumbo.biocloud.net/nelumbo/home). *Carica papaya* genome data were downloaded from https://phytozome-next.jgi.doe.gov/info/Cpapaya_ASGPBv0_4. The raw DNA sequencing read data used to generate the genome assemblies in this paper are available from the NCBI BioProject number PRJNA843598. The tissue-specific transcriptome data used for genome annotation and gene expression analysis in this paper were reported previously in Külahoglu et al. (2014) and were downloaded from NCBI BioProject numbers PRJNA237449 (for *G. gynandra*) and PRJNA237450 (for *T. hassleriana*). The *G. gynandra* cell type-specific transcriptome data were reported previously in Aubry et al. (2014) and were downloaded from NCBI BioSample numbers SAMN02719543 (BS cells) and SAMN02719544 (for M cells). Sequence alignments and machine-readable phylogenetic trees related to the phylogenetic analyses reported in the paper were deposited on FigShare at https://doi.org/10.6084/m9.figshare.21505380.v1. Seeds from the *G. gynandra* “GYN” accession will be available upon request from the corresponding author.

## Supplemental data

The following materials are available in the online version of this article.

**Supplemental Figure S1.** Genome size estimation of *G. gynandra*.

**Supplemental Figure S2.** Summary of the final *G. gynandra* genome assembly (v3.0).

**Supplemental Figure S3.** GO enrichment of 836 *G. gynandra*-specific orthogroups.

**Supplemental Figure S4.** Syntenic and colinear relationship among Cleomaceae and Brassicaceae genomes with the *G. gynandra* genome.

**Supplemental Figure S5.** Self–self syntenic dotplots of *C. violacea*, *G. gynandra* and *T. hassleriana* genomes.

**Supplemental Figure S6.** *Ks* distribution of syntenic gene pairs in the Cleomaceae and Brassicaceae genomes.

**Supplemental Figure S7.** Ratio of syntenic depth between genomes of *C. violacea* and *A. thaliana*, and between that of *C. violacea* and *B. rapa*.

**Supplemental Figure S8.** Phylogenetic relationships of *BCA4* gene copies identified from six Brassicaceae and Cleomaceae species used in this study, and their species tree.

**Supplemental Figure S9.** Phylogenetic trees of seven selected genes that show 1:2:3 syntenic relationship among *C. violacea*, *G. gynandra*, and *T. hassleriana* genomes.

**Supplemental Figure S10.** Duplicated genes of different modes of gene duplication identified by DupGen_finder across the five selected Cleomaceae and Brassicaceae genomes.

**Supplemental Figure S11.** *Ka* distribution of WGD-derived gene pairs from the five selected Brassicaceae and Cleomaceae genomes, and of different modes of gene duplication in the *G. gynandra* and *T. hassleriana* genomes.

**Supplemental Figure S12.** BUSCO completeness assessment of whole-genome assemblies and all transcripts from selected genomes used for analyses in this paper.

**Supplemental Table S1.** Summary statistics of libraries used for sequencing of the *G. gynandra* genome.

**Supplemental Table S2.** Summary statistics and BUSCO assessment of three versions of the *G. gynandra* genome.

**Supplemental Table S3.** Mapping-back rates of Illumina reads onto the *G. gynandra* genome assemblies from this study.

**Supplemental Table S4.** Summary statistics of the final *G. gynandra* genome assembly v3.0 by QUAST.

**Supplemental Table S5.** Summary statistics of repetitive elements in the final *G. gynandra* genome assembly v3.0.

**Supplemental Table S6.** Summary statistics of the predicted transcripts of the final *G. gynandra* genome assembly v3.0 by QUAST.

**Supplemental Table S7.** BUSCO completeness of the final *G. gynandra* genome assembly v3.0.

**Supplemental Table S8.** Summary of functional annotation of the *G. gynandra* genome.

**Supplemental Table S9.** Orthogroups of genes from five selected genomes by Orthofinder.

**Supplemental Table S10.** Summary of gene and site concordance factors of subgenome phylogenetic tree.

**Supplemental Table S11.** Summary statistics of different modes of gene duplication in the five selected genomes by DupGen_finder pipeline.

**Supplemental Table S12.** Summary statistics of *Ka/Ks* ratio of WDG gene pairs from five species.

**Supplemental Table S13.** Summary statistics of *Ka/Ks* ratio of gene pairs of different modes of gene duplication in *G. gynandra* and *T. hassleriana*.

**Supplemental Table S14.** Summary statistics of synteny analysis by SynFind.

**Supplemental Data Set 1.** Genome fractionation analyses of *G. gynandra* and *T. hassleriana*.

**Supplemental Data Set 2.** Reconciliation of gene-tree analysis by Notung.

**Supplemental Data Set 3.** ANOVA statistical results of *Ks* and *Ka/Ks*, and KEGG metabolic pathway enrichment analysis of duplicated genes.

**Supplemental Data Set 4.** Gene syntenic analyses by SynFind.

**Supplemental Data Set 5.** Tissue-specific and cell type-specific gene expression analyses.

## Supplementary Material

koad018_Supplementary_Data
