# Supplementary material for: The Gynandropsis gynandra genome provides insights into whole-genome duplications and the evolution of C4 photosynthesis in Cleomaceae
Source: Plant Cell. 2023 Jan 24;35(5):1334–59. doi: 10.1093/plcell/koad018 (PMC10118270; doi:10.1093/plcell/koad018)
Supplement: koad018_Supplementary_Data [file koad018_supplementary_data.zip › 04_Supplemental_Data_Figures_and_Tables_TPC_PROOF_correct.pdf]

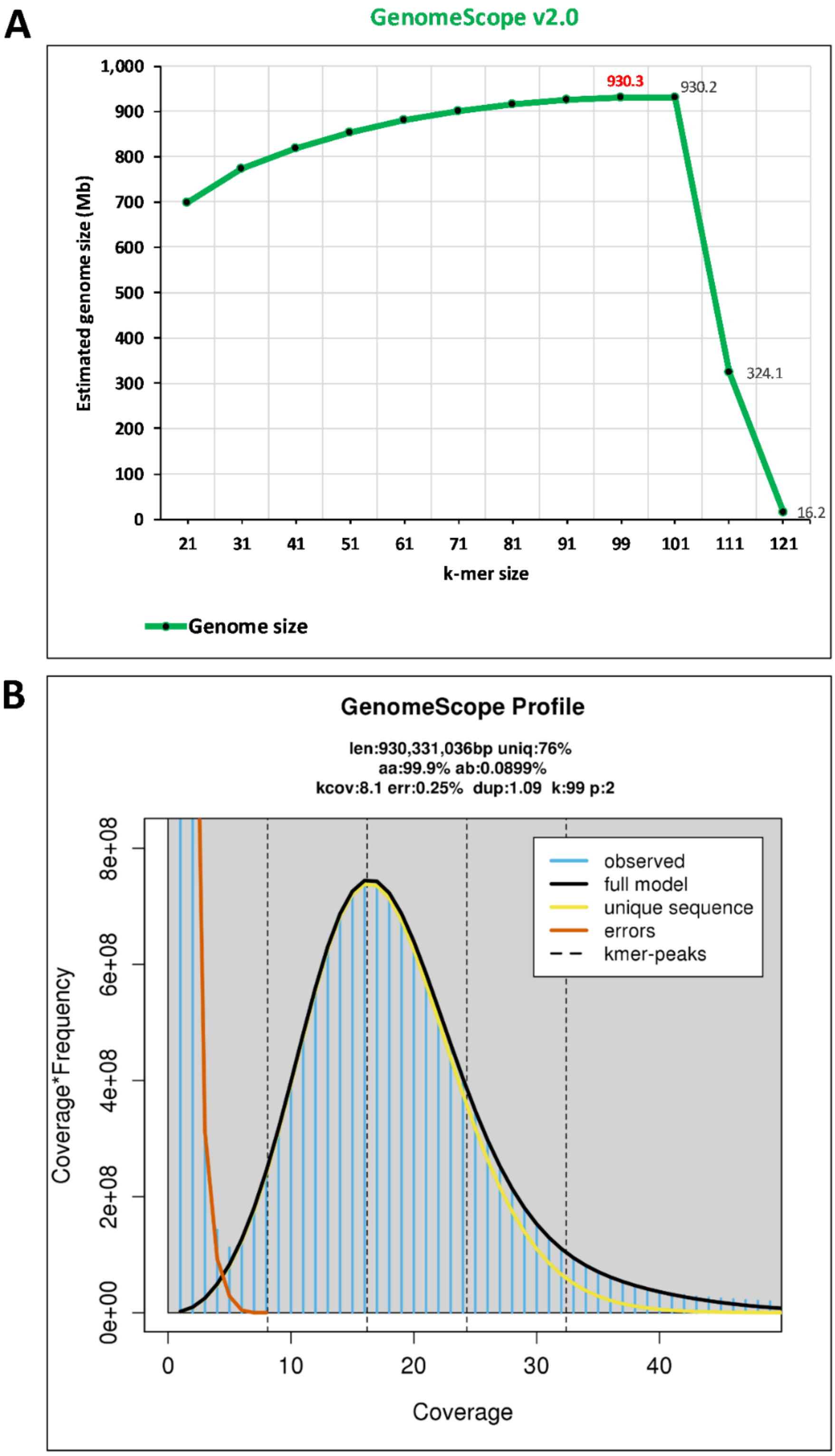

Supplemental Figure S1. Genome size estimation of *G. gynandra*. (A) Estimated genome size at different *k*-mer sizes ranging from 21 to 121. The estimation was based on a total 542,775,706 Illumina reads. Kmergenie (Chikhi and Medvedev, 2013) was also used to predict the best *k*-mer size, which is *k*-mer of 99. This is consistent with the result from GenomeScope that *k*-mer of 99 produced the largest estimated genome size (930.3 Mb) compared to that of other *k*-mer sizes within the range from 21 to 121. A large *k*-mer size likely better resolved the repetitive content in the genome. **(B)** Estimated genome size, heterozygosity and repeat content at *k*-mer of 99 by GenomeScope. **Supports Figure 1.**

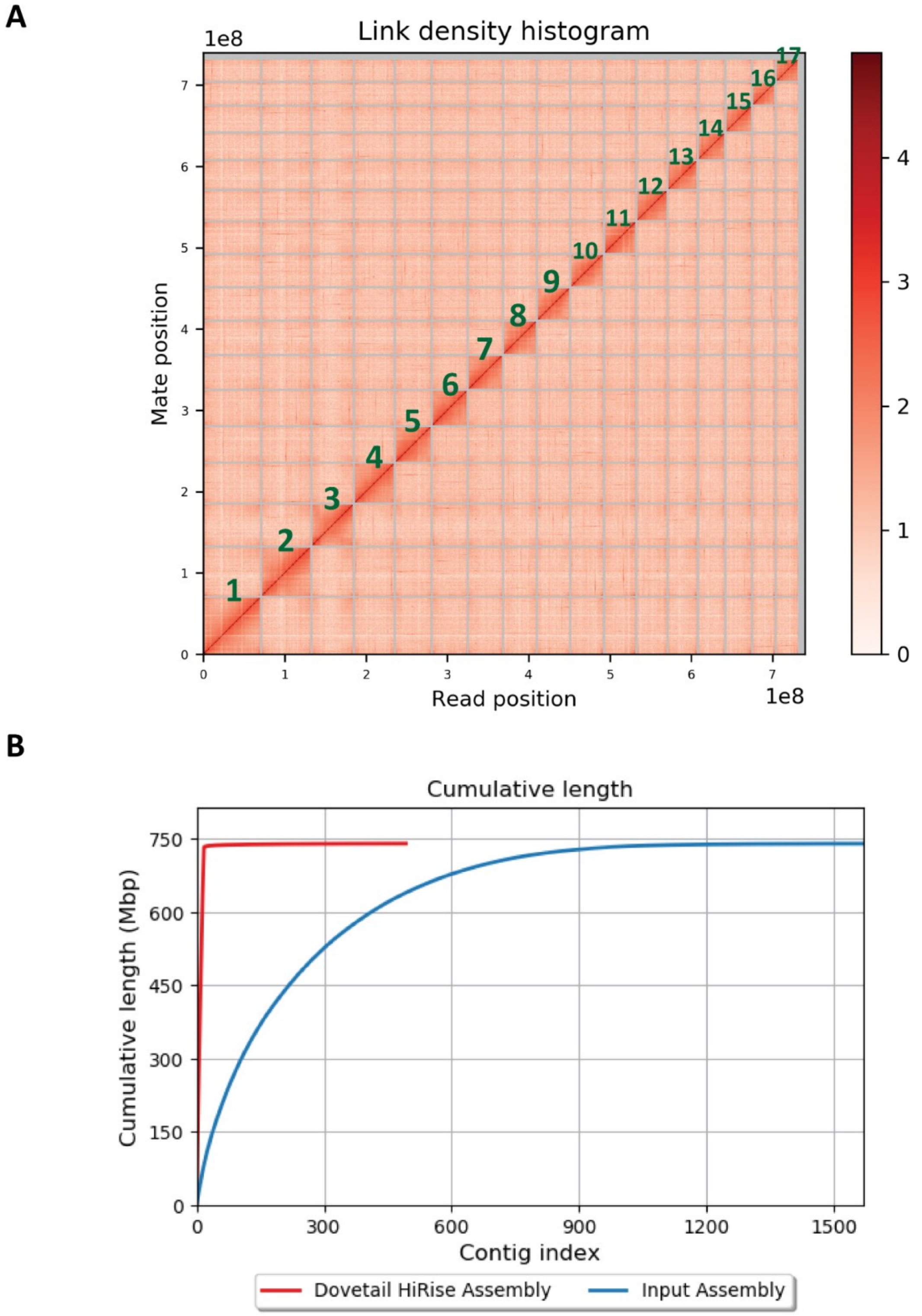

Supplemental Figure S2. Summary of the final *G. gynandra* genome assembly (v3.0). (A) Link density histogram of Hi-C scaffolding showing 17 major super-scaffolds. **(B)** Comparison of cumulative length of input assembly (v2.0) and Hi-C assembly (v3.0) showing a significant reduction of scaffold number. **Supports Figure 1.**

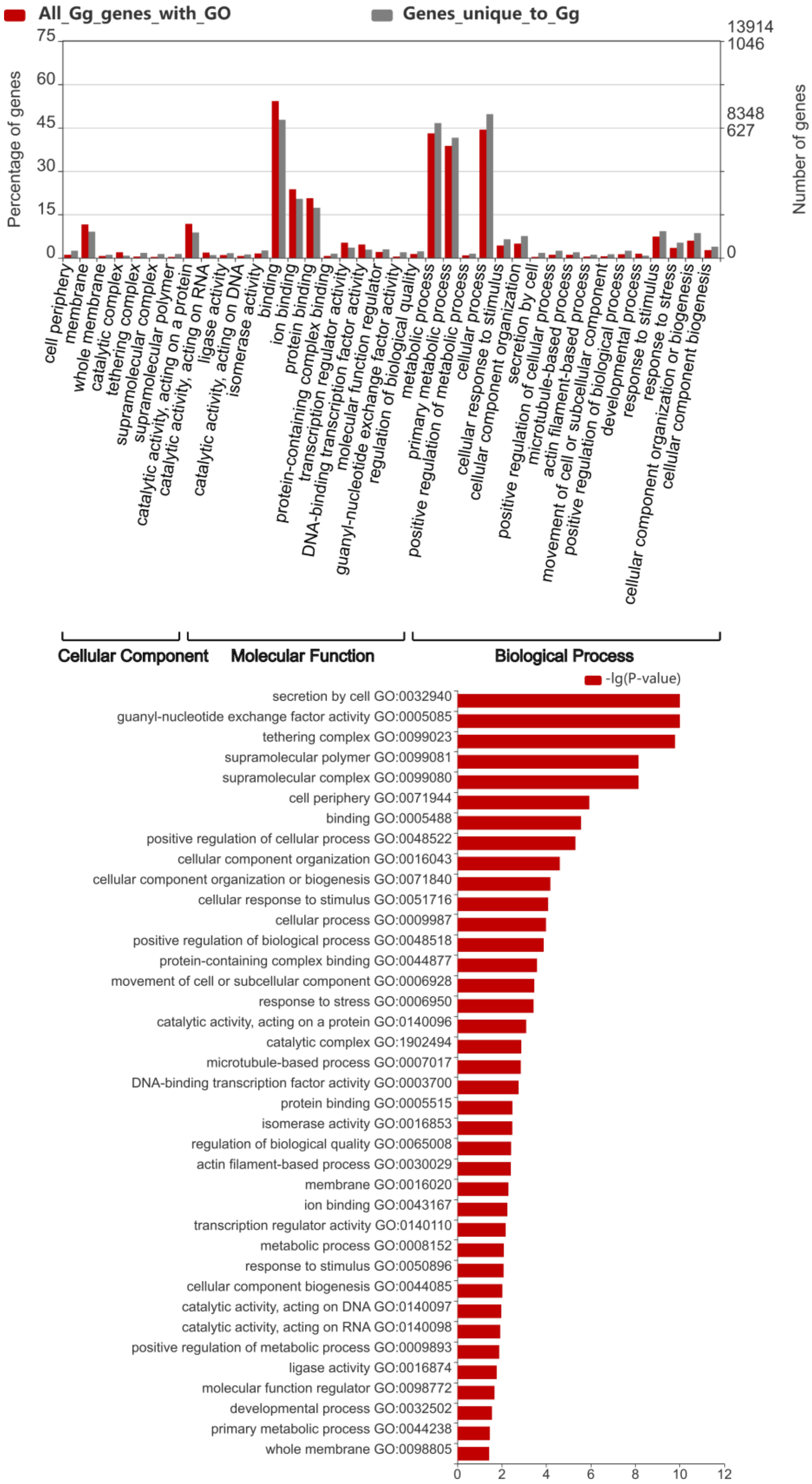

Supplemental Figure S3. GO enrichment of 836 *G. gynandra*-specific orthogroups. This contained 4,069 genes, of which, 2,010 genes were annotated at least one INTERPRO domain and 1,395 genes with at least one assigned GO term. All *G. gynandra* genes with GO terms were used as background. Significantly enriched GO terms ($p < 0.05$, Pearson Chi-square test) identified by WEGO program (Ye et al., 2018) between two datasets are shown in top panel, and the most significantly enriched terms are shown in bottom panel. **Supports Figure 1.**

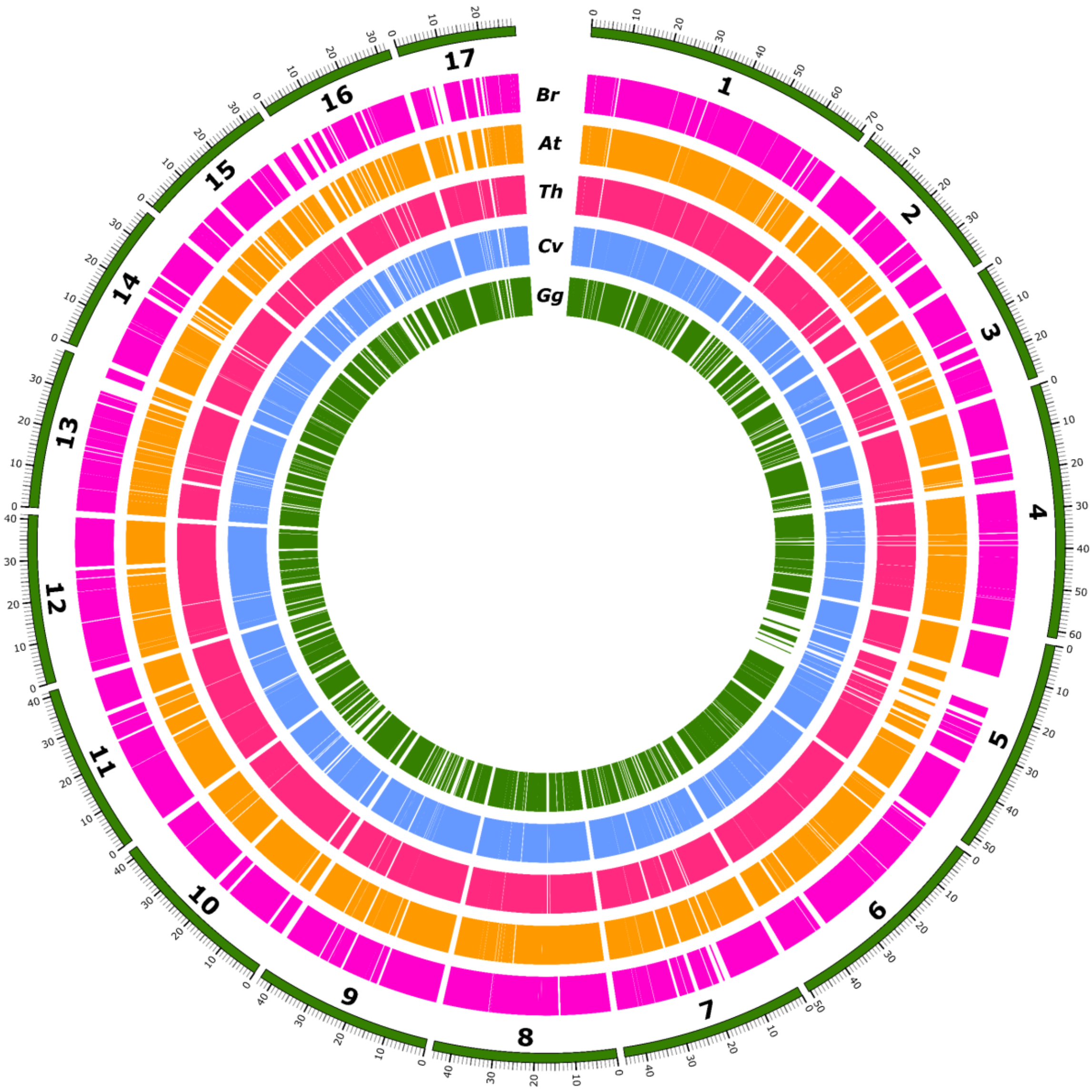

Supplemental Figure S4. Syntenic and colinear relationship among Cleomaceae and Brassicaceae genomes with the *G. gynandra* genome. The syntenic blocks from the five target genomes (including *G. gynandra* to itself) that were syntenic and colinear with the 17 super-scaffolds in the *G. gynandra* genome as a reference (outer tracks). The analysis was done using the JCVI package (a python version of MCscan) (Tang et al., 2008) with syntenic blocks (*minspan = 4 genes*). *Gg: G. gynandra, Cv: C. violacea, Th: T. hassleriana, At: A. thaliana, Br: B. rapa.* Scaffold length is in Mb. The figure only shows syntenic blocks of the target genomes that matched that of the *G. gynandra* genome; however, it does not show the overlapping syntenic blocks. If more than one syntenic block was found in the target genomes for the same reference block, only one is shown. **Supports Figure 1.**

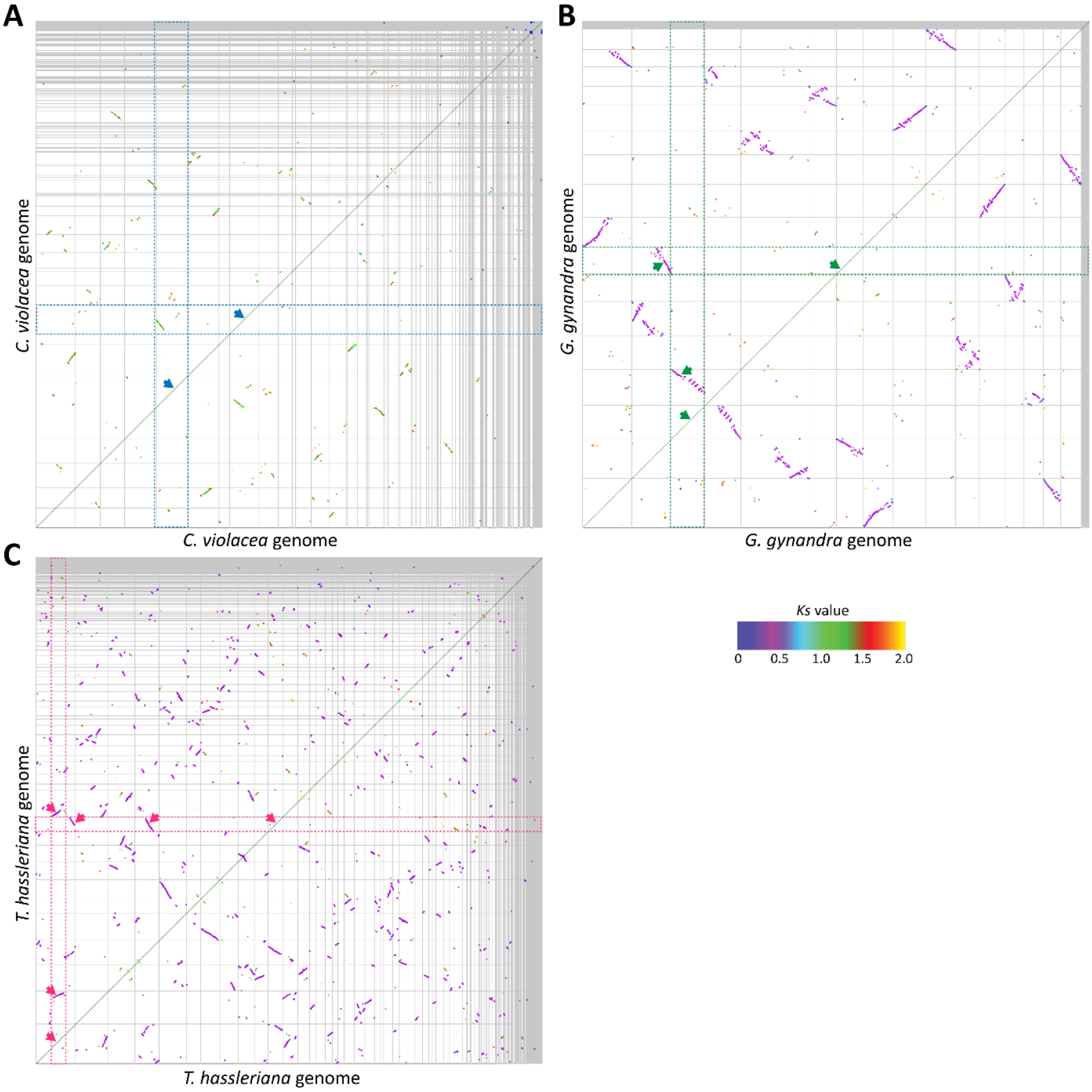

Supplemental Figure S5. Self-self syntenic dotplots of three Cleomaceae genomes used in this study. The comparisons are in the following order, *C. violacea* **(A)**, *G. gynandra* **(B)** and *T. hassleriana* **(C)**. Syntenic blocks were colored based on the *Ks* values (the ratio of the number of substitutions per synonymous site, representing sequence divergence time) of syntenic gene pairs between the syntenic blocks within each genome. Color scale is provided at the bottom right corner. The purple colors indicate the syntenic blocks originating from the recent WGD/WGT events (i.e., *Gg-α/Th-α*, *Ks* = ~0.5). Note that in *C. violacea,* most of the detected syntenic blocks are in green, red and yellow, which originated from the more ancient WGD event (i.e., *At-β*, *Ks* > 1) than those detected in *G. gynandra* and *T. hassleriana.* Horizontal and vertical gray lines separate scaffolds. The color arrows point to large intra-species syntenic signals for each species, with 1:1, 2:2 and 3:3 syntenic relationships, respectively. The dotplots were generated by SynMap program (Lyons et al., 2008) in the CoGe website (https://genomevolution.org/coge/). **Supports Figure 2.**

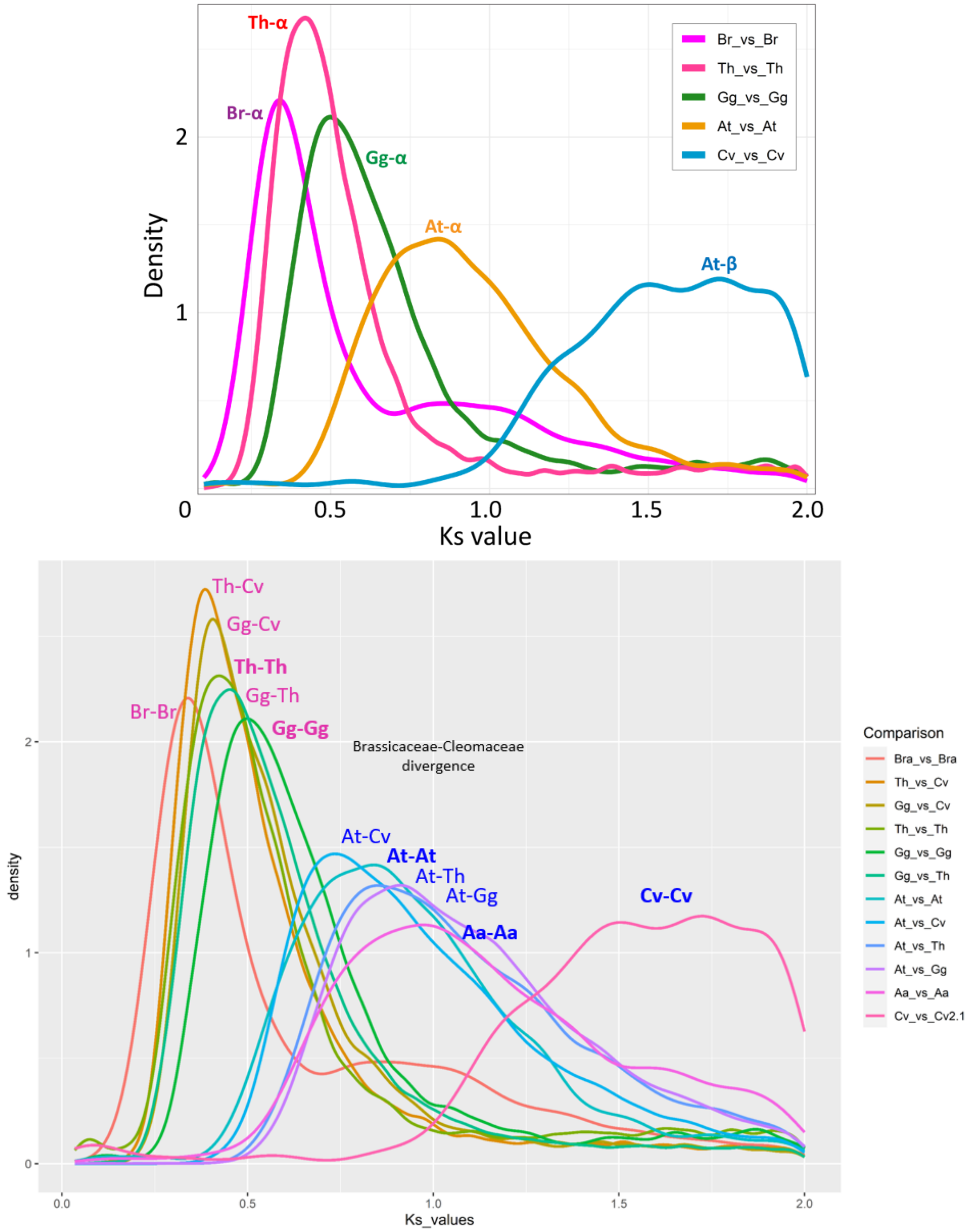

Supplemental Figure S6. *Ks* distribution of syntenic gene pairs in the Cleomaceae and Brassicaceae genomes. Top panel: *Ks* distribution of syntenic gene pairs of *C. violacea*, *G. gynandra***,** *T. hassleriana, A. thaliana* and *B. rapa* by intra-species self-comparison. **Bottom panel:** *Ks* distribution of syntenic gene pairs of intra- and inter-species comparisons showing peaks corresponding to speciation and WGD/WGT events. *Ks* was calculated for each of the intra-/inter-species syntenic gene pairs by SynMap (Lyons et al., 2008) in the CoGe website (https://genomevolution.org/coge/). Bold font indicates an intra-species comparison. Only *Ks* ≤ 2 were included in this analysis. Cv: *C. violacea*, At: *A. thaliana*, Aa: *A. arabicum*, Gg: *G. gynandra*, Th: *T. hassleriana*, and Br: *B. rapa.* **Supports Figure 2.**

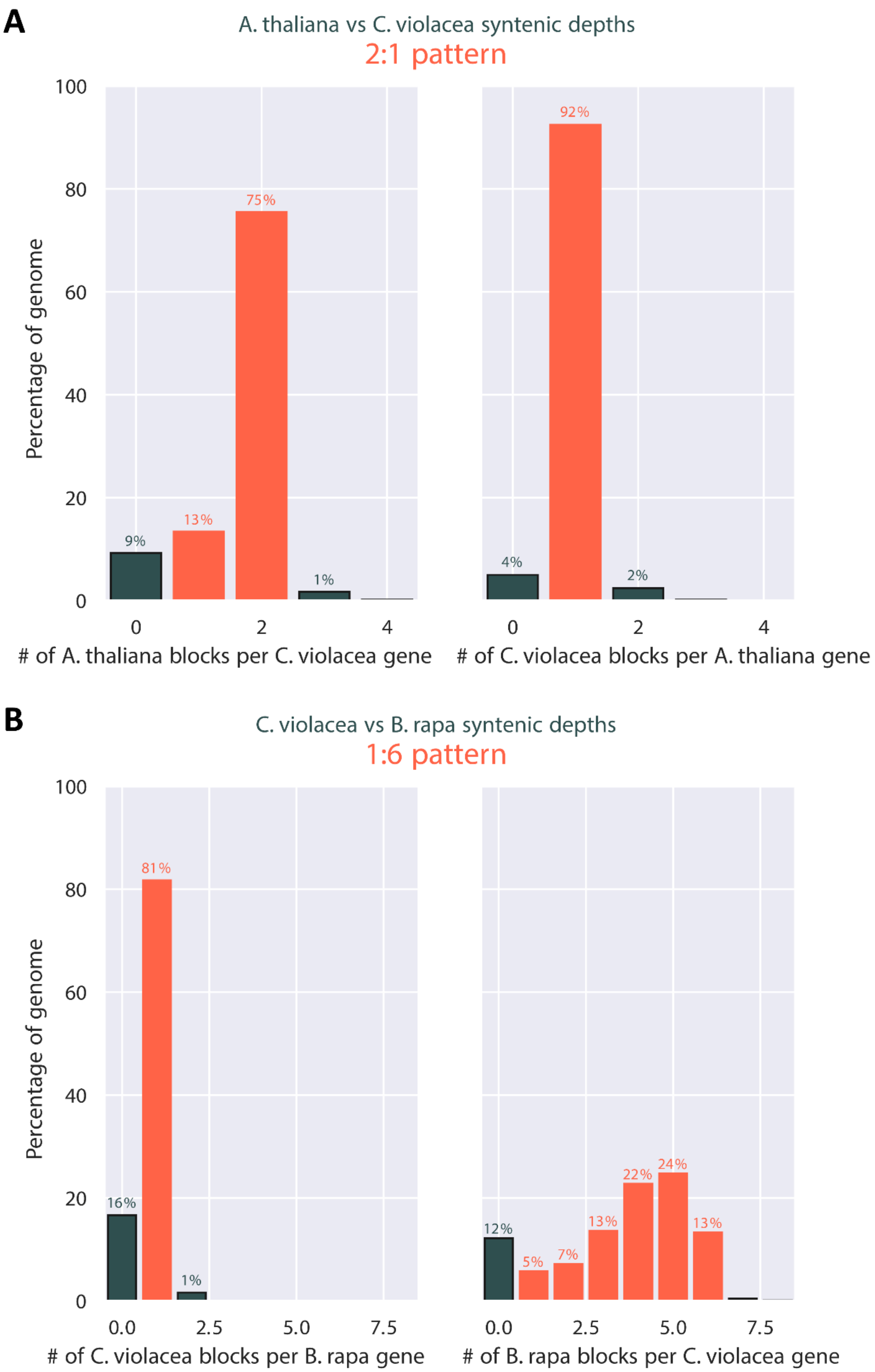

Supplemental Figure S7. Ratio of syntenic depth between genomes of *C. violacea* and *A. thaliana*, and between that of *C. violacea* and *B. rapa*. (**A**) Syntenic blocks of *A. thaliana* per *C. violacea* gene (left) and syntenic blocks of *C. violacea* per *A. thaliana* gene (right) which indicate a clear 2:1 pattern of *A. thaliana* to *C. violacea*. (**B**) Syntenic blocks of *C. violacea* per *B. rapa* gene (left) and syntenic blocks of *B. rapa* per *C. violacea* gene (right) which indicate a clear 1:6 pattern of *C. violacea* to *B. rapa*. **Supports Figure 2.**

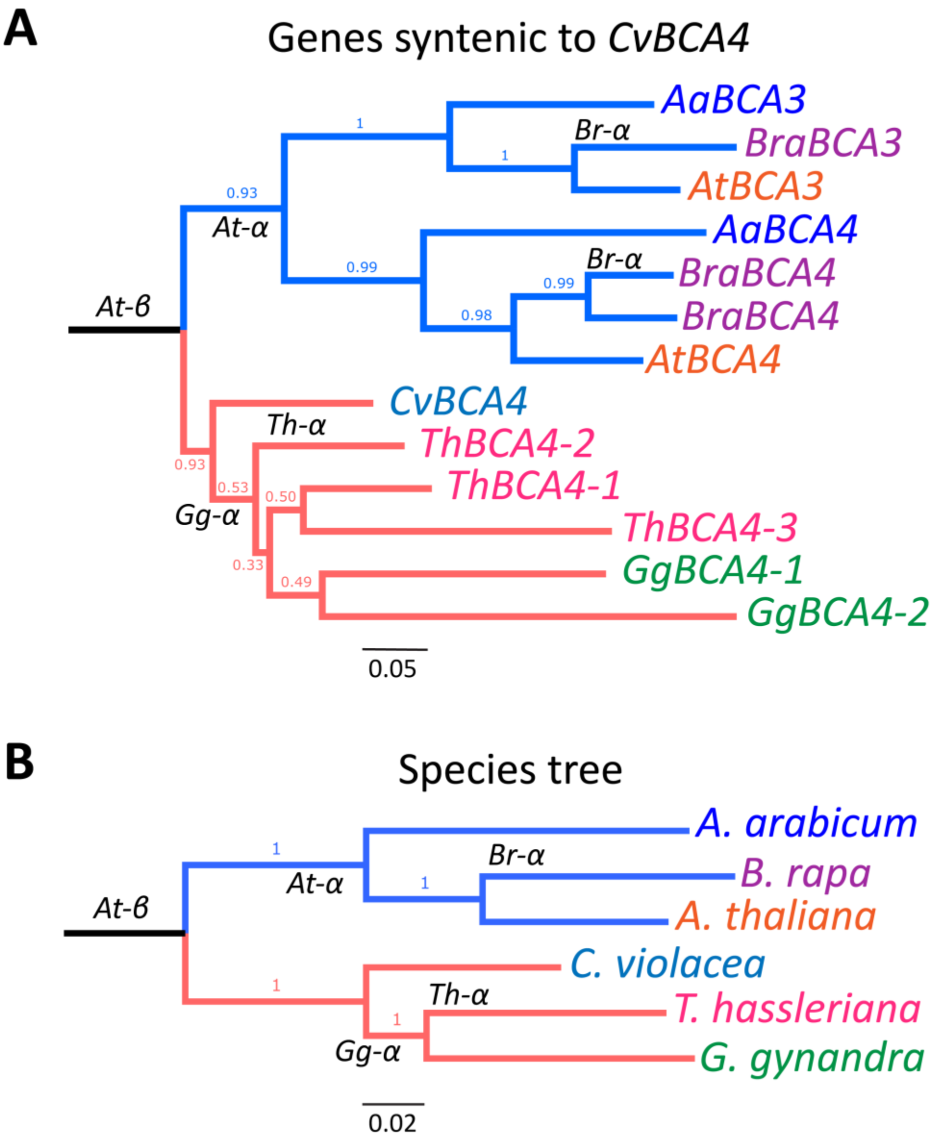

Supplemental Figure S8. Phylogenetic relationships of *BCA4* gene copies identified from six Brassicaceae and Cleomaceae species used in this study, and their species tree. (**A**) Phylogenetic relationships of *BCA4* genes from *A. arabicum*, *A. thaliana*, *B. rapa*, *C. violacea*, *G. gynandra* and *T. hassleriana.* Bayesian phylogeny was done using MrBayes (see **Methods**). Supporting values are posterior probabilities are given next to the branch. Branch length denotes substitutions per site. (**B**) Species tree of six Brassicaceae and Cleomaceae species used in this study. Tree was constructed based on 2,223 single-copy orthogroups among the six species. Midpoint rooting was used. Supporting values are bootstrap values (from FastTree Maximum Likelihood) and are given next to the branch. Branch length denotes substitutions per site. *BCA4*: *BETA CARBONIC ANHYDRASE4*. *Cv*: *C. violacea*, *At*: *A. thaliana*, *Aa*: *A. arabicum*, *Gg*: *G. gynandra*, *Th*: *T. hassleriana*, and *Br*: *B. rapa*. **Supports Figure 3.**

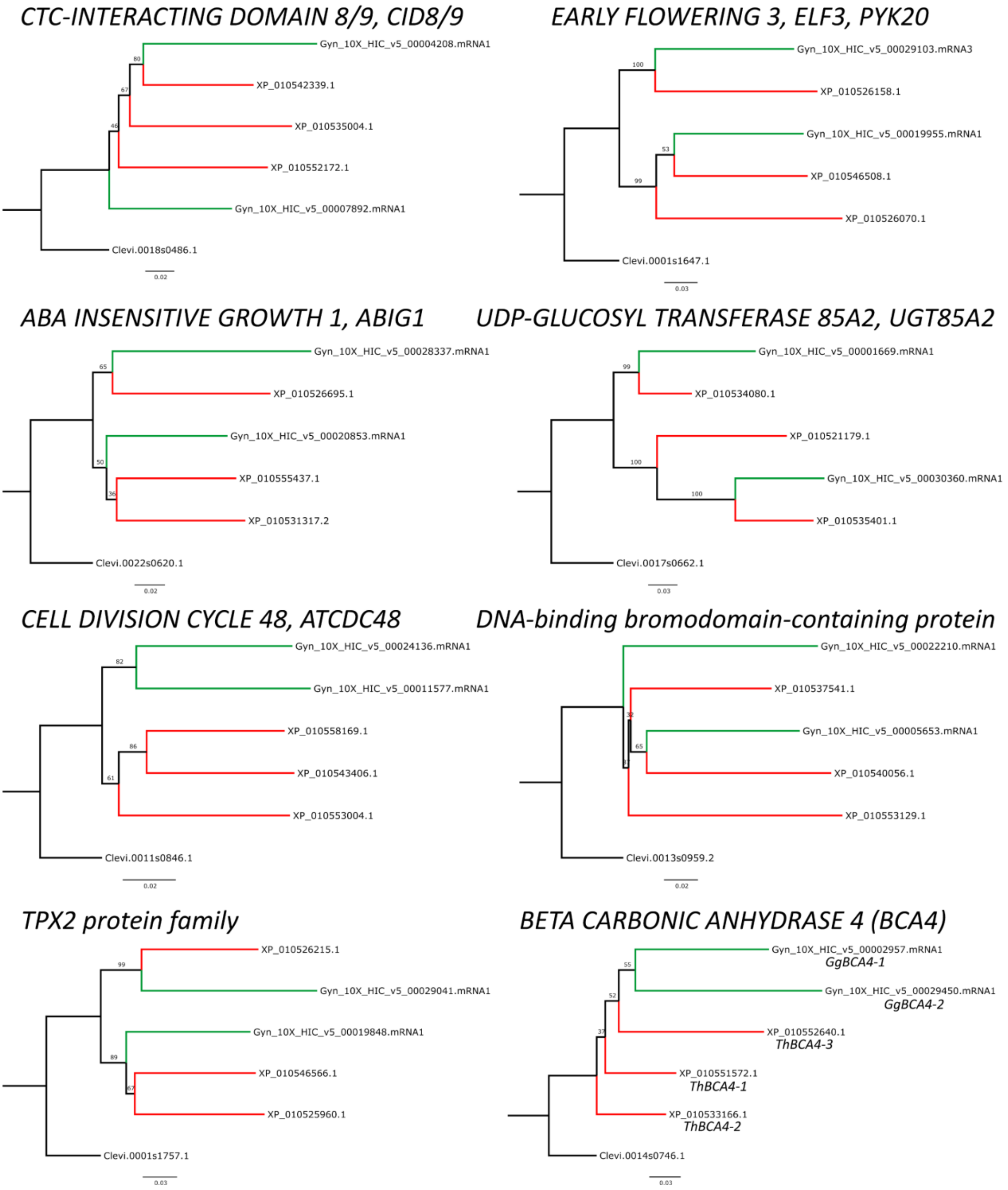

Supplemental Figure S9. Phylogenetic trees of eight selected genes that show 1:2:3 syntenic relationship among *C. violacea, G. gynandra* and *T. hassleriana* genomes. Gene names with prefixes “Clevi”, “Gyn” and “XP” are those from *C. violacea, G. gynandra* and *T. hassleriana*, respectively. For each gene tree, respective genes from *C. violacea* (*Clevi*) were used as outgroup. Branches corresponding to *G. gynandra* genes are in green, while those corresponding to *T. hassleriana* genes are in red color. Maximum likelihood phylogeny reconstruction was done using IQ-TREE. Supporting values are bootstrap values, and are given next to the branch. Branch length denotes substitutions per site. See **Methods** for more information related to phylogenetic tree construction. Note that a maximum likelihood tree was also constructed for *BCA4* genes. Different *BCA4* tree topologies between Figures S8 and S9 are likely due to the low supporting values from the single-gene phylogenetic approach. **Supports Figure 3.**

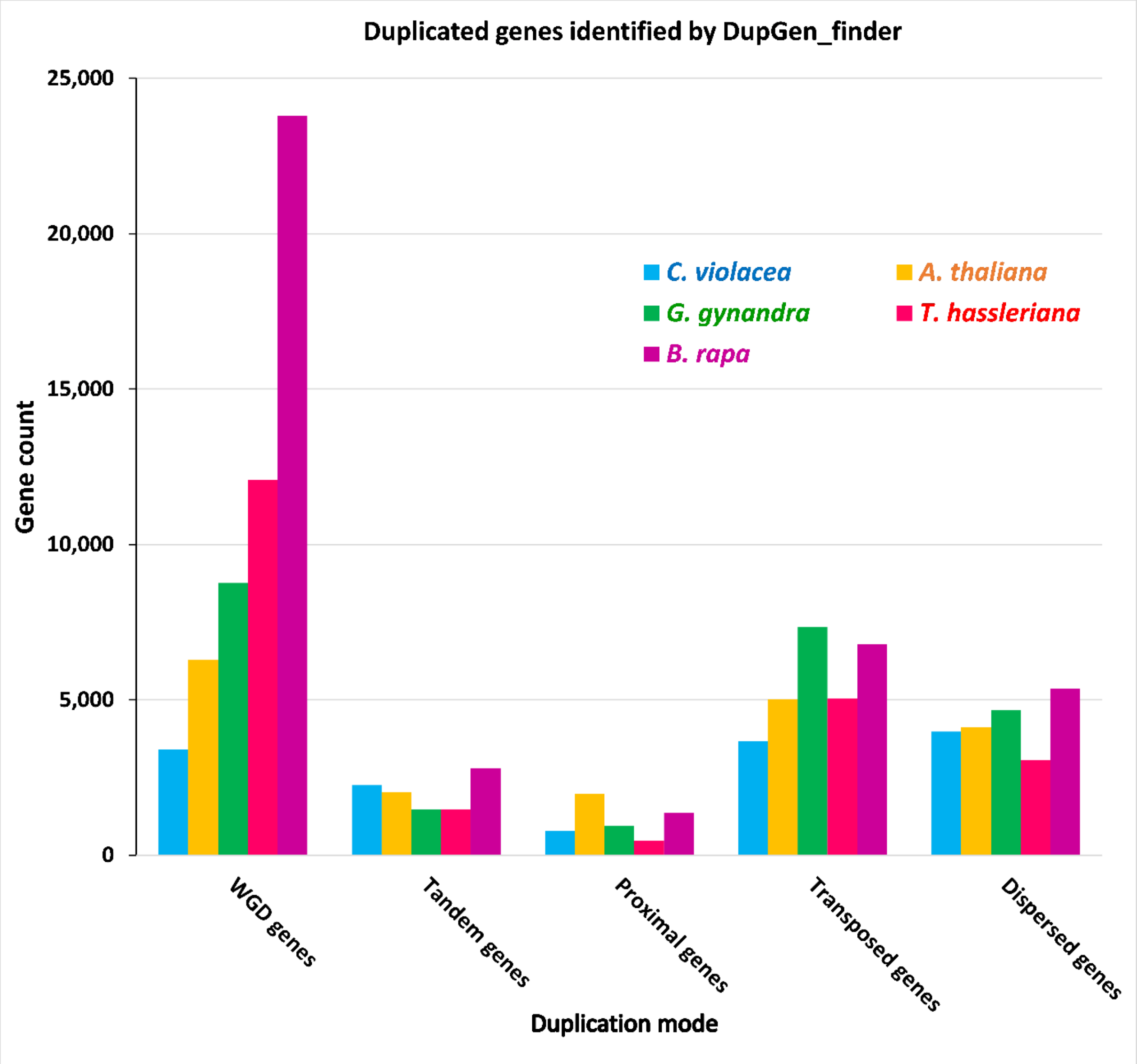

Supplemental Figure S10. Duplicated genes of different modes of gene duplication identified by *DupGen_finder* across the five selected Cleomaceae and Brassicaceae genomes. Unique gene counts were used after removing redundant matches within the dispersed gene pairs by *DupGen_finder*. Duplicated genes were identified within each genome using *Nelumbo nucifera* (the sacred lotus) as outgroup. **Supports Figure 4.**

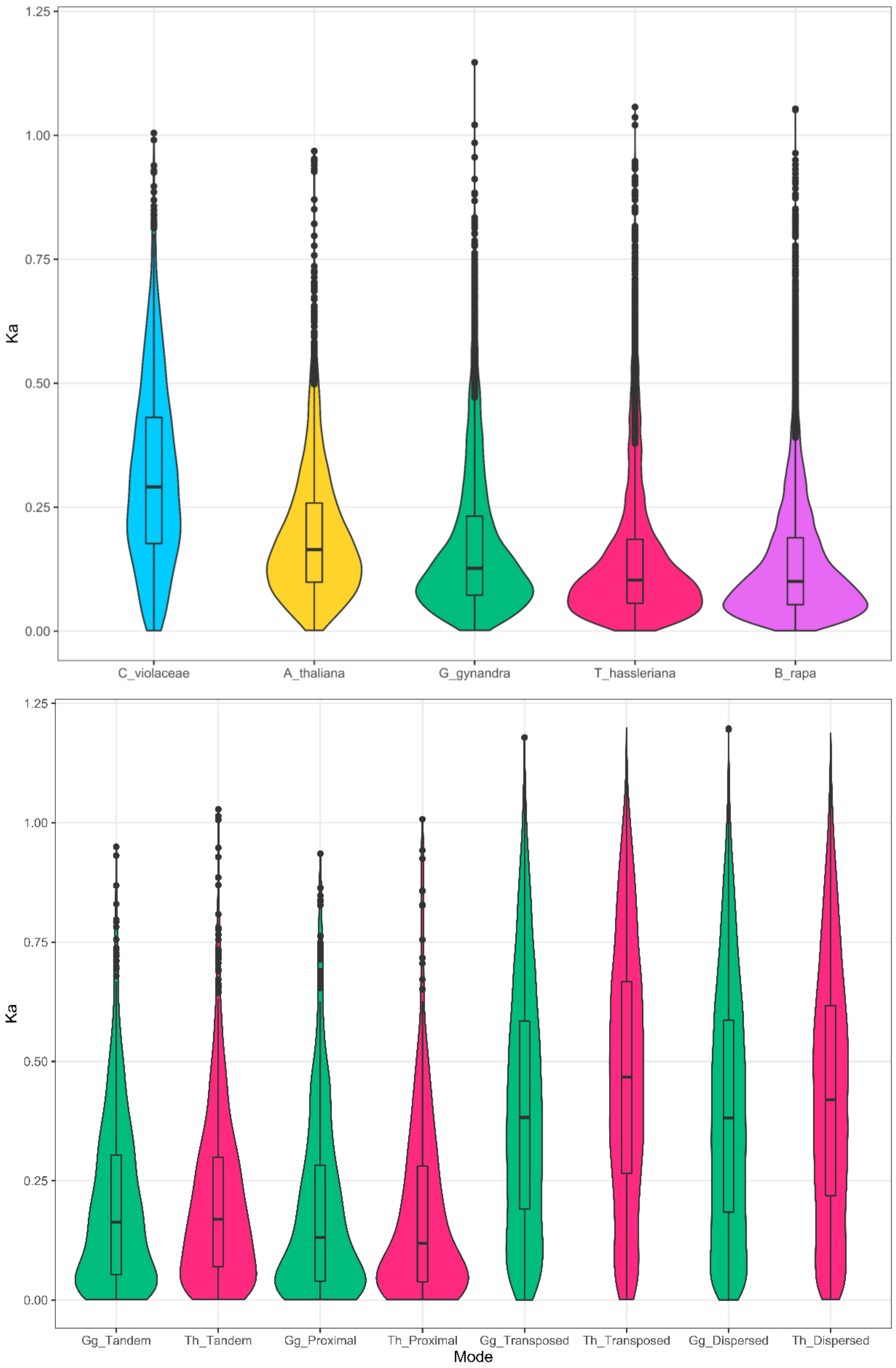

Supplemental Figure S11. *Ka* distribution of different modes of gene duplication. (**A**) *Ka* distribution of WGD-derived gene pairs from the five selected Brassicaceae and Cleomaceae genomes. (**B**) *Ka* distribution of different modes of gene duplication in the *G. gynandra* and *T. hassleriana* genomes. **Supports Figure 4.**

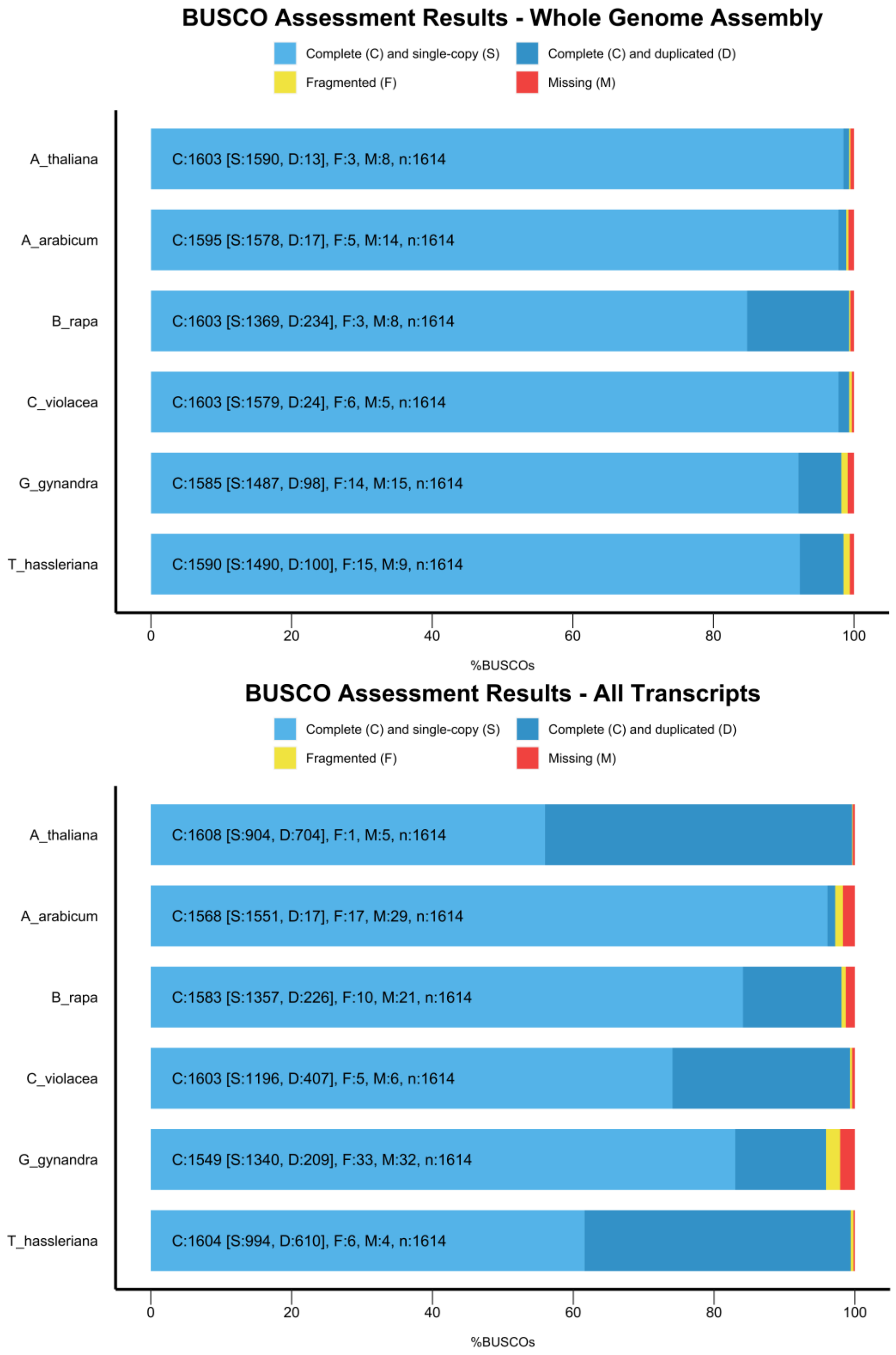

Supplemental Figure S12. BUSCO completeness assessment of whole genome assemblies and all transcripts from selected genomes used for analyses in this paper. The BUSCO v5.3.2 and the plant-specific Embryophyta odb10 dataset which included 1,614 BUSCO proteins (Simão et al., 2015) were used. Information related to data availability can be found within the section "**Data availability statement**" within the main paper. For *A. thaliana*, TAIR10 genome sequences and araport11 gene annotation were used. **Supports Methods** and **Figure 4.**

Supplemental Table S1. Summary statistics of libraries used for sequencing of the *G. gynandra* genome.

**Illumina data for genome v1.0**			
**Insert-size PE library (bp)**	**Read length (bp)**	**Raw reads (Gp)**	**Trimmed reads (Gb)**
250	150	25.5	18.7
350	100	31.8	25.4
500	100	37.8	28.0
800	100	13.4	11.6
2,000	90	32.4	17.6
5,000	90	31.3	15.7
10,000	90	14.9	4.0
20,000	90	22.6	3.6
**Total bases (Gb)**		**209.6**	**124.7**
**Coverage (fold)***		**209.6**	**124.7**

**10X genomics data for genome v2.0**					
**Sample**	**Read count**	**Read length (bp)**	**Total bases**	**Trimmed reads (Q20)**	**% Trimmed reads**
cleome_S5_L005_R1_001.fastq.gz	271,387,853	150	40,708,177,950	36,035,408,035	88.52
cleome_S5_L005_R2_001.fastq.gz	271,387,853	150	40,708,177,950	32,359,625,832	79.49
**Totals**	542,775,706	150	81,416,355,900	68,395,033,867	84.01
**Coverage (fold)***			**81.42**	**68.40**	

**HiRise data for genome v3.0**	
**Read-pairs**	71,334,013
**Number of joins made by HiRise**	1,484
**Number of breaks made to input assembly by HiRise**	390

**Calculated using the estimated genome size of ~1 Gb for G. gynandra*

Supplemental Table S2. Summary statistics and BUSCO assessment of three versions of the *G. gynandra* genome.

**Assembly**	***G_gynandra_v1.0***	***G_gynandra_v2.0***	***G_gynandra_v3.0***
# scaffolds (>= 0 bp)	1,241,060	1,693	616
Total length (>= 0 bp)	1,042,892,128	739,819,666	739,969,522
Largest scaffold (bp)	3,513,483	5,871,673	70,998,242
GC (%)	38.69	37.69	37.69
N50 (bp)	293,254	1,376,588	41,873,000
N90 (bp)	127	340,244	32,916,692
L50	898	151	8
L90	368,504	564	15
# N's per 100 kb	10,283	9,937	9,955

**BUSCO completeness**	***G_gynandra_v1.0***	***G_gynandra_v2.0***	***G_gynandra_v3.0***
Total completeness (%)	98.1	98.2	98.2
Complete BUSCOs (C)	1,583	1,584	1,585
Complete and single-copy BUSCOs (S)	1,485	1,486	1,487
Complete and duplicated BUSCOs (D)	98	98	98
Fragmented BUSCOs (F)	16	15	14
Missing BUSCOs (M)	15	15	15
Total BUSCO groups searched	1,614	1,614	1,614

**Annotations**	***G_gynandra_v1.0_CDS***	***G_gynandra_v2.0_CDS***	***G_gynandra_v3.0_CDS***
# transcripts (>= 0 bp)	35,490	34,639	33,748
# transcripts (>= 1,000 bp)	15,833	15,217	15,210
Total length (>= 0 bp)	39,579,711	39,062,782	38,499,471
Total length (>= 1,000 bp)	28,762,005	28,508,072	28,270,425
Largest transcript (bp)	21,180	14,334	16,725
GC (%)	46.24	45.90	46.05

N50 (bp)	1,485	1,524	1,524
N90 (bp)	561	561	567
L50	8,529	7,984	7,961
L90	24,728	23,865	23,488
# N's per 100 kb	854	12	4

Supplemental Table S3. Mapping back rates of Illumina reads onto the *G. gynandra* genome assemblies from this study.

**Sequencing read data information**	
***G. gynandra* accession**	**GYN - Malaysia**
**Total reads in pairs - Q30**	410,877,922
**Total bases**	58,340,126,267
**Genome coverage (fold)***	58.34

**Mapping results**	
**Genome version – Aligner**	**Mapping rate (%)**
*G_gynandra*_v3.0_Bowtie2	97.47
*G_gynandra*_v3.0_BWA-MEM	95.65
*G_gynandra*_v2.0_Bowtie2	97.47
*G_gynandra*_v1.0_Bowtie2	97.96

**Calculated using the estimated genome size of ~1 Gb for G. gynandra*
Bowtie2 (https://bowtie-bio.sourceforge.net/bowtie2/index.shtml) running with default settings and -local alignment option
BWA-MEM (https://github.com/bwa-mem2/bwa-mem2) default settings. Mapping rate from BWA-MEM was calculated by subtracting the percentage of unmapped reads from the total reads (100%).

Supplemental Table S4. Summary statistics of the final *G. gynandra* genome assembly v3.0 by QUAST.

**Assembly**	***Gynandropsis_gynandra_v3.0***
# scaffolds (>= 0 bp)	616
# scaffolds (>= 1,000 bp)	493
# scaffolds (>= 5,000 bp)	267
# scaffolds (>= 10,000 bp)	157
# scaffolds (>= 25,000 bp)	70
# scaffolds (>= 50,000 bp)	42
Total length (>= 0 bp)	739,969,522
Total length (>= 1,000 bp)	739,892,407
Total length (>= 5,000 bp)	739,292,116
Total length (>= 10,000 bp)	738,559,045
Total length (>= 25,000 bp)	737,265,796
Total length (>= 50,000 bp)	736,345,651
Largest scaffold (bp)	70,998,242
GC (%)	38
N50 (bp)	41,873,000
N90 (bp)	32,916,692
L50	8
L90	15
# N's per 100 kb	9,955

**Scaffold name**	**Length (bp)**	**% length of top 17 scaffolds**
Scaffold_1_2_contigs_length_70998242	70,998,242	99.0
Scaffold_2_1_contigs_length_39919855	39,919,855	
Scaffold_3_1_contigs_length_28822934	28,822,934	
Scaffold_4_2_contigs_length_61691101	61,691,101	
Scaffold_5_3_contigs_length_52406510	52,406,510	
Scaffold_6_2_contigs_length_50417563	50,417,563	
Scaffold_7_1_contigs_length_45170229	45,170,230	
Scaffold_8_4_contigs_length_44541640	44,541,640	
Scaffold_9_2_contigs_length_43053523	43,053,523	
Scaffold_10_1_contigs_length_41872999	41,873,000	
Scaffold_11_2_contigs_length_41251205	41,251,205	
Scaffold_12_2_contigs_length_41042610	41,042,610	
Scaffold_13_1_contigs_length_38709322	38,709,323	
Scaffold_14_2_contigs_length_36694614	36,694,614	
Scaffold_15_3_contigs_length_34118300	34,118,300	
Scaffold_16_1_contigs_length_32916691	32,916,692	
Scaffold_17_2_contigs_length_28949651	28,949,651	
The rest (599 scaffolds)	7,392,529	

Supplemental Table S5. Summary statistics of repetitive elements in the final *G. gynandra* genome assembly v3.0.

Total length: 739,969,522 bp (666,353,826 bp excl N/X-runs)
Bases masked: 509,079,492 bp (68.80 %)

**Class/Family**			**Number of elements***	**Length occupied (bp)**	**Percentage of sequence (%)**
**Retroelements**			359,174	314,097,346	42.45
	SINEs:		726	104,858	0.01
	LINEs:		11,633	5,058,931	0.68
	LTR elements:		346,815	308,933,557	41.75
		Ty1/Copia	143,253	128,224,223	17.33
		Gypsy/DIRS1	156,677	159,589,213	21.57
**DNA transposons**			164,595	94,093,391	12.72
**Rolling-circles**			14,273	8,451,532	1.14
**Unclassified:**			263,288	83,879,766	11.34
**Total interspersed repeats:**				**492,070,503**	**66.5**
**Small RNA:**			4,612	810,810	0.11
**Satellites:**			4,541	1,005,703	0.14
**Simple repeats:**			118,580	5,868,219	0.79
**Low complexity:**			18,473	965,804	0.13

** Most repeats fragmented by insertions or deletions have been counted as one element*
RepeatMasker version 4.1.2-p1 , default mode run with rmblastn version 2.11.0+

Supplemental Table S6. Summary statistics of the predicted transcripts of the final *G. gynandra* genome assembly v3.0 by QUAST.

**All transcripts**	***Gynandropsis_gynandra*_v3.0**
# transcripts (>= 0 bp)	33,748
# transcripts (>= 1,000 bp)	15,210
# transcripts (>= 5,000 bp)	235
# transcripts (>= 10,000 bp)	20
# transcripts (>= 25,000 bp)	0
Total length (>= 0 bp)	38,499,471
Total length (>= 1,000 bp)	28,270,425
Total length (>= 5,000 bp)	1,572,123
Total length (>= 10,000 bp)	246,135
Total length (>= 25,000 bp)	0
Largest transcript (bp)	16,725
GC (%)	46.05
N50 (bp)	1,524
N90 (bp)	567
L50	7,961
L90	23,488
# N's per 100 kb	4.31

Supplemental Table S7. BUSCO completeness of the final *G. gynandra* genome assembly v3.0.

Whole genome assembly - Metaeuk gene prediction

**C:98.2%[S:92.1%,D:6.1%],F:0.9%,M:0.9%,n:1614**	
1,585	Complete BUSCOs (C)
1,487	Complete and single-copy BUSCOs (S)
98	Complete and duplicated BUSCOs (D)
14	Fragmented BUSCOs (F)
15	Missing BUSCOs (M)
1,614	Total BUSCO groups searched

Annotations

**C:97.1%[S:88.4%,D:8.7%],F:1.5%,M:1.4%,n:1614**	
1,566	Complete BUSCOs (C)
1,426	Complete and single-copy BUSCOs (S)
140	Complete and duplicated BUSCOs (D)
25	Fragmented BUSCOs (F)
23	Missing BUSCOs (M)
1,614	Total BUSCO groups searched

All transcripts

**C:95.9%[S:83.0%,D:12.9%],F:2.0%,M:2.1%,n:1614**	
1,549	Complete BUSCOs (C)
1,340	Complete and single-copy BUSCOs (S)
209	Complete and duplicated BUSCOs (D)
33	Fragmented BUSCOs (F)
32	Missing BUSCOs (M)
1,614	Total BUSCO groups searched

BUSCO version is: 5.3.2
The lineage dataset is: embryophyta_odb10 (Creation date: 2020-09-10, number of genomes: 50, number of BUSCOs: 1614)

Supplemental Table S8. Summary of functional annotation of the *G. gynandra* genome.

		Number	Percent (%)
	**Total genes**	30,933	
**Annotated with**	**Swiss-Prot**	24,068	77.8
	**TrEMBL**	27,460	88.8
	**InterPro**	24,794	80.2
	**GO**	18,553	60.0
	**KEGG**	12,573	40.6
	**Total annotated**	28,209	91.2
	**Unannotated**	2,724	8.8

Supplemental Table S9. Orthogroups of genes from five selected genomes by Orthofinder.

Overall statistics

Number of species	5
Number of genes	154,083
**Number of genes in orthogroups**	**144,039**
Number of unassigned genes	10,044
Percentage of genes in orthogroups	94
Percentage of unassigned genes	7
**Number of orthogroups**	**21,977**
Number of species-specific orthogroups	2,984
Number of genes in species-specific orthogroups	13,709
Percentage of genes in species-specific orthogroups	9
Mean orthogroup size	7
Median orthogroup size	6
G50 (assigned genes)	7
G50 (all genes)	7
O50 (assigned genes)	6,670
O50 (all genes)	7,388
**Number of orthogroups with all species present**	**14,266**
Number of single-copy orthogroups	3,746

Per species

	A_thaliana	***B_rapa***	***C_violacea***	***G_gynandra***	***T_hassleriana***
Number of genes	27,654	46,250	21,850	30,933	27,396
Number of genes in orthogroups	25,912	41,565	21,191	28,806	26,565
Number of unassigned genes	1,742	4,685	659	2,127	831
Percentage of genes in orthogroups	93.7	89.9	97.0	93.1	97.0
Percentage of unassigned genes	6	10	3	7	3
Number of orthogroups containing species	**17,881**	**18,822**	**17,210**	**16,997**	**16,840**

Percentage of orthogroups containing species	81	86	78	77	77
Number of species-specific orthogroups	331	1,509	170	836	138
Number of genes in species-specific orthogroups	1,150	6,966	757	4,069	767
Percentage of genes in species-specific orthogroups	4	15	4	13	3

OrthologuesStats_Totals

	A_thaliana	***B_rapa***	***C_violacea***	***G_gynandra***	***T_hassleriana***
A_thaliana	0	23,510	21,224	19,859	20,752
B_rapa	33,173	0	29,564	28,073	29,237
C_violacea	18,586	18,029	0	**17,828**	**18,825**
G_gynandra	22,661	22,565	**22,565**	0	23,084
T_hassleriana	23,769	23,199	**23,745**	23,047	0

Orthogroups_SpeciesOverlaps

	A_thaliana	***B_rapa***	***C_violacea***	***G_gynandra***	***T_hassleriana***
A_thaliana	17,881	16,974	15,873	15,012	15,594
B_rapa	16,974	18,822	15,553	14,891	15,360
C_violacea	**15,873**	**15,553**	**17,210**	**15,711**	**16,271**
G_gynandra	**15,012**	**14,891**	**15,711**	**16,997**	**15,572**
T_hassleriana	15,594	15,360	16,271	15,572	16,840

Supplemental Table S10. Summary of gene and site concordance factors of subgenome phylogenetic tree.

ID	gCF	gCF_N	gDF1	gDF1_N	gDF2	gDF2_N	gDFP
9	61.54	32	11.54	6	3.85	2	23.08
10	40.38	21	1.92	1	13.46	7	44.23
11	36.54	19	13.46	7	19.23	10	30.77

ID	gDFP_N	gN	sCF	sCF_N	sDF1	sDF1_N	sDF2	sDF2_N	sN	Quartet score	Branch length
9	12	52	45.69	42.99	23.15	21.59	31.16	28.91	93.487	[q1=0.69;q2=0.12;q3=0.19]	0.712
10	23	52	42.22	39.91	22.34	21.02	35.44	33.52	94.446	[q1=0.55;q2=0.26;q3=0.19]	0.376
11	16	52	26.47	25.64	40.51	39.40	33.01	32.03	97.074	[q1=0.48;q2=0.3;q3=0.22]	0.233

*Gene and site concordance factors. ID: Branch ID, see **Fig. 3**; gCF: Gene concordance factor (=gCF_N/gN %); gCF_N: Number of trees concordant with the branch; gDF1: Gene discordance factor for NNI-1 branch (=gDF1_N/gN %); gDF1_N: Number of trees concordant with NNI-1 branch; gDF2: Gene discordance factor for NNI-2 branch (=gDF2_N/gN %); gDF2_N: Number of trees concordant with NNI-2 branch; gDFP: Gene discordance factor due to polyphyly (=gDFP_N/gN %); gDFP_N: Number of trees decisive but discordant due to polyphyly; gN: Number of trees decisive for the branch; sCF: Site concordance factor averaged over 1000 quartets (=sCF_N/sN %); sCF_N: sCF in absolute number of sites; sDF1: Site discordance factor for alternative quartet 1 (=sDF1_N/sN %); sDF1_N: sDF1 in absolute number of sites; sDF2: Site discordance factor for alternative quartet 2 (=sDF2_N/sN %); sDF2_N: sDF2 in absolute number of sites; sN: Number of informative sites averaged over 1000 quartets; Quartet score: ASTRAL quartet score; Length: Branch length.*

Supplemental Table S11. Summary statistics of different modes of gene duplication in the five selected genomes by *DupGen_finder* pipeline

**Types**	***C_violacea***	***A_thaliana***	***G_gynandra***	***T_hassleriana***	***B_rapa***	***Nelumbo nucifera* (outgroup)**
**Total genes**	**21,850**	**27,654**	**30,933**	**27,396**	**46,250**	34,486
**WGD pairs**	2,008	3,308	4,976	8,301	20,474	
**Tandem pairs**	1,539	1,390	1,038	1,133	2,347	
**Proximal pairs**	553	1,404	693	394	1,244	
**Transposed pairs**	3,737	5,105	7,471	5,154	7,076	
**Dispersed pairs**	12,174	15,803	19,119	16,900	29,278	
**Total gene pairs**	**20,011**	**27,010**	**33,297**	**31,882**	**60,419**	
**WGD ratio to *C. violacea***	**1**	**1.6**	**2.5**	**4.1**	**10.2**	

Unique priority: WGD > tandem > proximal > transposed > dispersed.

**Types**	***C_violacea***	***A_thaliana***	***G_gynandra***	***T_hassleriana***	***B_rapa***
**WGD pairs**	2,008	3,308	4,976	8,301	20,474
**Tandem pairs**	1,362	1,116	754	686	1,218
**Proximal pairs**	427	981	440	201	513
**Transposed pairs**	3,737	5,105	7,471	5,154	7,076
**Dispersed pairs**	4,479	4,445	5,674	4,675	9,877
**Total unique gene pairs**	**12,013**	**14,955**	**19,315**	**19,017**	**39,158**

For number of duplicated genes in each genome, see ***Supplemental Figure S10****. The number of dispersed gene pairs reduced after the removal of low confident pairs by DupGen_finder.*

Supplemental Table S12. Summary statistics of *Ka/Ks* ratio of WDG gene pairs from five species.

	C_violacea	***A_thaliana***	***G_gynandra***	***T_hassleriana***	***B_rapa***
Mean	0.134	0.153	0.176	0.184	0.201
Standard Error	0.003	0.002	0.002	0.001	0.001
Median	0.106	0.131	0.145	0.156	0.166
Standard Deviation	0.134	0.101	0.132	0.130	0.152
Sample Variance	0.018	0.010	0.017	0.017	0.023
Range	2.257	0.733	1.643	1.587	2.861
Minimum	0.002	0.002	0.001	0.001	0.001
Maximum	2.259	0.735	1.644	1.588	2.862
Sum	258.45	402.96	873.68	1,509.39	4,069.68
Count	1,931	2632	4,955	8,212	20,248

Supplemental Table S13. Summary statistics *of Ka/Ks* ratio of gene pairs of different modes of gene duplication in *G. gynandra* and *T. hassleriana*.

	Gg_WGD	*Th_WGD*	*Gg_Tandem*	*Th_Tandem*	*Gg_Proximal*	*Th_Proximal*	*Gg_Transposed*	*Th_Transposed*	*Gg_Dispersed*	*Th_Dispersed*
Mean	0.176	0.184	0.378	0.344	0.449	0.402	0.259	0.164	0.277	0.178
Standard Error	0.002	0.001	0.012	0.009	0.015	0.017	0.003	0.002	0.002	0.001
Median	0.145	0.156	0.257	0.264	0.337	0.313	0.179	0.145	0.173	0.146
Standard Deviation	0.132	0.130	0.357	0.282	0.357	0.331	0.240	0.110	0.289	0.142
Sample Variance	0.017	0.017	0.128	0.079	0.127	0.109	0.058	0.012	0.083	0.020
Range	1.643	1.587	2.839	2.729	2.497	2.832	2.903	1.679	2.918	2.231
Minimum	0.001	0.001	0.017	0.007	0.016	0.009	0.001	0.001	0.001	0.004
Maximum	1.644	1.588	2.856	2.736	2.513	2.841	2.904	1.681	2.919	2.235
Sum	874	1,509	361	364	272	147	1,896	819	5,197	2,931
Count	4,955	8,212	954	1,059	605	365	7,317	4,989	18,739	16,512

***Ka/Ks*>1**										
Count	9	4	54	36	47	14	108	6	561	57
%>1	0.18	0.05	5.66	3.40	7.77	3.84	1.48	0.12	2.99	0.35
%≤1	99.82	99.95	94.34	96.60	92.23	96.16	98.52	99.88	97.01	99.65
Total	100	100	100	100	100	100	100	100	100	100

***Ka/Ks*>0.5**										
Count	118	210	241	203	205	97	922	70	2,916	534
%>0.5	2.38	2.56	25.26	19.17	33.88	26.58	12.60	1.40	15.56	3.23
%≤0.5	97.62	97.44	74.74	80.83	66.12	73.42	87.40	98.60	84.44	96.77

Total	100	100	100	100	100	100	100	100	100	100

***Ka/Ks*>0.25**										
Count	1,071	2,004	492	552	379	217	2,427	646	5,994	2,742
%>0.25	21.61	24.40	51.57	52.12	62.64	59.45	33.17	12.95	31.99	16.61
%≤0.25	78.39	75.60	48.43	47.88	37.36	40.55	66.83	87.05	68.01	83.39
Total	100	100	100	100	100	100	100	100	100	100

Supplemental Table S14. Summary statistics of synteny analysis by SynFind.

Total syntenic regions (proxies + syntelogs)

	C_violacea	*A_thaliana*	*G_gynandra*	*T_hassleriana*
**Depth:0**	0	1,100	0	0
**Depth:1**	14,612	2,715	1,705	1,393
**Depth:2**	9,086	14,993	14,068	4,866
**Depth:3**	2,091	5,769	6,731	12,207
**Depth:4**	430	1,452	2,662	4,803
**Depth:>4**	70	260	1,123	3,020
**Total**	26,289	26,289	26,289	26,289

Syntenic genes (syntelogs) only

	C_violacea	*A_thaliana*	*G_gynandra*	*T_hassleriana*
**Depth:0**	0	2,678	0	0
**Depth:1**	17,605	13,148	12,728	11,932
**Depth:2**	3,474	5,067	7,189	6,857
**Depth:3**	385	537	1,228	2,145
**Depth:4**	40	68	310	427
**Depth:>4**	1	7	50	144
**Total**	21,505	21,505	21,505	21,505

Depth = region or gene count

REFERENCES

Chikhi, R., and Medvedev, P. (2013). Informed and automated k-mer size selection for genome assembly. Bioinformatics **30,** 31-37.

Lyons, E., Pedersen, B., Kane, J., and Freeling, M. (2008). The Value of Nonmodel Genomes and an Example Using SynMap Within CoGe to Dissect the Hexaploidy that Predates the Rosids. Trop. Plant Biol. **1,** 181-190.

Simão, F.A., Waterhouse, R.M., Ioannidis, P., Kriventseva, E.V., and Zdobnov, E.M. (2015). BUSCO: assessing genome assembly and annotation completeness with single-copy orthologs. Bioinformatics **31,** 3210-3212.

Tang, H., Bowers, J.E., Wang, X., Ming, R., Alam, M., and Paterson, A.H. (2008). Synteny and Collinearity in Plant Genomes. Science **320,** 486-488.

Ye, J., Zhang, Y., Cui, H., Liu, J., Wu, Y., Cheng, Y., Xu, H., Huang, X., Li, S., Zhou, A., Zhang, X., Bolund, L., Chen, Q., Wang, J., Yang, H., Fang, L., and Shi, C. (2018). WEGO 2.0: a web tool for analyzing and plotting GO annotations, 2018 update. Nucleic Acids Res. **46,** W71-W75.
