# Supplementary material for: The Gynandropsis gynandra genome provides insights into whole-genome duplications and the evolution of C4 photosynthesis in Cleomaceae
Source: Plant Cell. 2023 Jan 24;35(5):1334–59. doi: 10.1093/plcell/koad018 (PMC10118270; doi:10.1093/plcell/koad018)
Supplement: koad018_Supplementary_Data [file tpc22.01103.PeerReviewReport.pdf › tpc22.01103.PeerReviewReport.pdf]

The genome of *Gynandropsis gynandra* provides insights into whole-genome duplications and the evolution of C_4 photosynthesis in Cleomaceae

Nam V. Hoang, E. O. Deedi Sogbohossou, Wei Xiong, Conor J. C. Simpson, Pallavi Singh, Nora Walden, Erik van den Bergh, Frank F. M. Becker, Zheng Li, Xin-Guang Zhu, Andrea Brautigam, Andreas P. M. Weber, Jan C. van Haarst, Elio G. W. M. Schijlen, Prasad S. Hendre, Allen Van Deynze, Enoch G. Achigan-Dako, Julian M. Hibberd, M. Eric Schranz

Corresponding author: M. Eric Schranz (eric.schranz@wur.nl).

Review timeline:

TPC2022-RA-00664	Submission received:	July 11, 2022
	1st Decision:	September 7, 2022 *manuscript declined*
**TPC2022-RA-01103**	Submission received:	November 9, 2022
	1st Decision:	December 16, 2022 *accept with minor revisions*
**TPC2022-RA-01103R1**	1st Revision received:	December 17, 2022
	2nd Decision:	December 29, 2022 *acceptance pending, sent to sci editor*
	Final acceptance:	Jan. 18, 2022

REPORT: (The report shows the major requests for revision and author responses. Minor comments for revision and miscellaneous correspondence are not included. The original format may not be reflected in this compilation, but the reviewer comments and author responses are not edited, except to correct minor typographical or spelling errors that could be a source of ambiguity.)

TPC2021-RA-00537 1st Editorial decision – *declined* September 7, 2022

Thank you for choosing to send your manuscript entitled "The genome of *Gynandropsis gynandra* provides insights into whole-genome duplications and the evolution of C4 photosynthesis in Cleomaceae" for consideration at The Plant Cell. Your submission has been evaluated by members of the editorial board as well as expert reviewers in your field, and we regret to inform you that we are not able to recommend publication of this manuscript in its current form. We have not made this decision lightly. We have had input from multiple scientists, and we have solicited post-review comments as well. Our present policy is to offer streamlined decisions and to not advise on the direction of the work by requesting extensive modifications or substantial additional experiments.

We would be willing to re-consider a new manuscript that fully addressed the concerns raised during this review process. If you decide to resubmit to The Plant Cell, it will be evaluated as a new submission subject to full assessment by the editorial board, and if sent for external review, a new set of reviewers might be chosen. Reviewers will be asked to assess your work as a new manuscript (i.e. are the claims fully supported by the data and do the results presented move the field forward?), and not only whether previous reviewer comments have been addressed.

That said, the three sets of expert reviewer comments provide a comprehensive set of suggestions for digging in more deeply into the genome and gene orthogroups to make stronger inferences about the genome duplication/fractionation process and the evolution of C4 photosynthesis. The manuscript was submitted for publication as a Large-Scale Biology Article, and addressing the reviewer comments would demonstrate the utility of the *Gynandropsis gynandra* genome as a significant value to the broader plant science community.

It will be important to convince the editors and reviewers that the study adds significant new understanding(s) of mechanisms or processes, or otherwise represents a significant advance in the field, and that the major claims made are fully justified by the data presented. This includes careful consideration and explanation of the various controls used in experiments, the extent and manner of replication, and the statistical analyses used. Sampling methods and the nature of "biological replicates" should be described precisely (i.e. different plants, parts of plants, pooled tissue, independent pools of tissue, sampled at different times, etc.), along with a clear description of and rationale for any

statistical analyses conducted. The reader should know exactly what was sampled; what forms the basis of the calculation of any means and other statistical variables and parameters reported. This is also necessary to ensure that proper statistical analysis was conducted.

--- Reviewer comments:

TPC2022-RA-01103 Submission received	November 9, 2022

Reviewer comments on previously declined manuscript and **author responses:**

Reviewer #1:

Hoang et al., presented a new genome sequence of *Gynandropsis gynandra*, a C4 species in Cleomaceae with various economic values. By combining Illumina sequencing, 10X Genomics sequencing and Hi-C technologies, the authors could finally construct a relatively high-quality genome assembly, and anchored majority of the assemblies onto 17 super-scaffolds. Then, they focused on the WGD history in Cleomaceae and the evolutionary analyses of C4 gene families. I would agree the new genomic resources of G. gynandra could open up diverse research avenues as well as the breeding purpose. In general, I think the manuscript is well written, but I also have several questions and comments as listed below.

Comment 1. The first is about the completeness of the genome assembly. As the authors presented, the estimated genome size is about 930Mb while the total length of the final assembled sequences is about 740Mb. The BUSCO completeness assessment of *G. gynandra* also exhibits relatively lower than other compared species as illustrated in **Fig. S11**. I would suggest the authors to remap these generated raw reads back to the assembly to check the mapping rates, so we can understand why there are 190Mb (930-740) differences between the estimated the genome size and the total length of the assembled sequences.

The authors would like to thank the reviewer for the positive feedback and the helpful suggestion. To address this, we utilized a total of ~411 million high-quality Illumina paired-end reads (Phred score ≥Q30, representing a 58-fold coverage of the *G. gynandra* genome) that were used during our genome assembly (Supplemental Table S3). Reads were mapped back onto the final *G. gynandra* genome assembly v3.0 (from this paper) using two aligners, BWA-MEM (v0.7.17) (Li, 2013) with default settings and Bowtie2 (v2.4.5) (Langmead and Salzberg, 2012) with default settings and "local alignment" mode. Overall, BWA-MEM reported 95.7%, while Bowtie2 reported 97.8% reads mapped onto our final genome assembly. We further estimated the completeness of the genome assembly (as suggested by Reviewer #3) using BUSCO v5.3.2 (Simão et al., 2015) and Metaeuk gene predictor searching against 1,614 BUSCOs in the embryophyta_odb10 database. This revealed that our assembly contains 98.2% complete BUSCOs, a relatively high percentage and comparable to that of *A. thaliana*, *A. arabicum*, *B. rapa*, *C. violacea* and *T. hassleriana* (98.5-99.3%, see Supplemental Figure S12). Collectively, this indicates that our assembly well represents the gene space within the *G. gynandra* genome, and the difference between our assembly and the predicted genome size (740Mb vs. 930Mb, respectively) is therefore largely attributed to the high repetitive content of the *G. gynandra* genome (Beric et al., 2021). A proportion of repetitive content might have been collapsed together during the assembly/scaffolding steps, but still present in the assembly hence raw reads could be mapped to it. We have now added these to the related text in the manuscript, including lines 151-175 and Supplemental Table S2. It is important to note here that the BUSCO scores reported in our previous version of the manuscript were based on the extracted CDS sequences from the annotation, while the new BUSCO score were based on *de novo* prediction of genes in the assembly.

Additionally, as suggested by Reviewer #3, we included comparative statistics for our genome assembly v1.0 (1.04 Gb) and v2.0 (739.8 Mb) together with the final assembly v3.02 (740 Mb) (Supplemental Table S2). The data showed that while the v3.0's genome size was reduced, its BUSCO completeness was slightly improved compared to that of v1.0 (98.1 to 98.2%). This further supports our argument that the difference between predicted genome size and assembly size was due to repetitive content.

Comment 2. I am not sure whether the authors had generated sufficient transcriptome data for the genome annotation process. Therefore, it is not clear how many of these 30,933 gene models with transcriptome support.

Thank you for this point. We have clarified Methods (lines 682-683) to make it clear that transcriptome data of 18

different tissues of *G. gynandra* previously reported in Külahoglu et al. (2014) were utilized for annotation. These transcriptome data sets contain samples from different developmental stages of the major tissues/organs of a *G. gynandra* plant including leaf, stem, root, seed, seedling, sepal, stamen, petal and carpel (see Figure 7 for a subset of 16 samples from the original data). To check how many predicted gene models have transcriptome support, we remapped these data to our predicted gene models (details are provided in a newly added section "RNA-seq analysis of tissue- and cell type-specific gene expression" in the updated Methods, lines 832-846). Overall, we found that 30,013 genes (97%) of the total 30,933 gene models were supported by the transcriptome data (TPM, transcripts per million transcripts, >0). We now incorporated this information into the main text (lines 176-186) and added one track to Figure 1A showing the expression level of gene models across the *G. gynandra* pseudo-molecules using leaf gradient data (Leaf_0 to Leaf_5) from Külahoglu et al. (2014).

Comment 3. The WGD history in Cleomaceae seems complicated. Based on these presented analyses, it is indeed hard to determine whether G. gynandra and T. hassleriana have shared one WGD or not. It seems these presented several gene family trees exhibiting low resolution and inconsistent topologies (Fig 3B and Fig S8). I suggest the authors to run a large_scale phylogenomic analyses about these syntenic anchor genes, especially for these anchor genes in good syntenic blocks with the ratio of 2 G. gynandra : 3 T. hassleriana. Then, the authors could check the topologies of high-resolution trees for these anchor genes. It might (or might also not) help to elucidate the WGD history of Cleomaceae.

We followed the reviewer's valuable suggestion and conducted a phylogenetic analysis of genes with a 1:2:3 pattern. The analysis was restricted to syntenic genes on the same syntenic blocks in order to select reliable homologs reflecting the evolutionary history of polyploidization and speciation and to exclude paralogs derived from other gene duplication events (Walden and Schranz, 2022). For this, we first reconstructed subgenomes of *G. gynandra* (Figure 3A) and *T. hassleriana* (Figure 3B) based on their synteny and gene density compared to the *C. violacea* genome. This resulted in *G. gynandra* having two subgenomes (least fractionated and most fractionated, *Gg*LF and *Gg*MF, respectively) while *T. hassleriana* having three subgenomes (*Th*LF, medium and most fractionated, *Th*MF$_1$ and *Th*MF$_2$). Due to high fractionation rates in the *Th*MF$_2$ genome (see Figure 3B), the final dataset for phylogenetic analysis comprised 52 genes from four well supported syntenic blocks on different scaffolds relative to the *C. violacea* genome (see Supplemental Data Set 1). These correspond to 104 *G. gynandra* and 156 *T. hassleriana* syntenic genes. Because single-gene trees often showed low resolution and inconsistent topologies, we conducted species-tree reconstruction with the ASTRAL program (Zhang et al., 2018) and analyzed gene and site concordance factors. The species-tree approach resulted in a phylogenetic tree consistent with a WGD shared by *G. gynandra* and *T. hassleriana*, and the addition of a third genome equivalent to the *T. hassleriana* lineage after the speciation event giving rise to both lineages (Figure 3D). We added related texts to lines 291-313, Supplemental Data Set 1 and Supplemental Table S10.

Additionally, we now provide a reconciliation gene tree analysis using Notung (Stolzer et al., 2012) to estimate gene duplications and phylogenic placement of the WGD/WGT in the Cleomaceae (Figure 3E). This result supports the notion that the *Gg-α* WGD event is shared by several nested clades within the Cleomaceae family including *Gynandropsis*, *Tarenaya*, *Melidiscus*, African clades, and probably also Andean, *Cleoserrata* and *Dactylaena* clades. The *Th-α* event corresponds to the one that resulted in the third subgenome *Th*LF being added to *T. hassleriana* after its divergence from *G. gynandra*. We updated the related text in lines 314-331.

Comment 4. It is rather interesting to compare the C4 plant of G. gynandra to the C3 plant of T. hassleriana. The authors found the higher retention rate of WGD duplicates in G. gynandra than that of T. hassleriana, and also found some other duplication mechanisms also providing gene copies for the C4 photosynthesis pathway. Although I am not an expert of C4, it seems to me that we are still hard to conclude that these duplicates helped the evolution or origination of C4 pathway in G. gynandra. Some additional comparisons of these C4 genes between G. gynandra and T. hassleriana are needed. Any key genes are missing in G. gynandra? Are these duplicates in G. gynandra are functional/expressed?

We agree with the reviewer. We have now incorporated gene expression analysis of C_4 genes using the tissue/organ-specific transcriptome atlas reported in Külahoglu et al. (2014). This dataset contains paired data of key tissues/organs from the two species *G. gynandra* and *T. hassleriana.* We also analyzed these genes using cell type-specific RNA-seq data from Aubry et al. (2014) that were derived from *G. gynandra* mesophyll (M) and bundle sheath (BS) cells isolated by laser capture microdissection (LCM). The results (Figure 7C) highlight that, among the

duplicated gene copies retained in each gene family after the recent WGD/WGT event, one of the *G. gynandra* copies (in bold green) is highly expressed in the photosynthetic tissues (i.e., leaf and seedling) compared to other copies in both *G. gynandra* and *T. hassleriana*. The expression of these highly expressed gene copies was generally increased during leaf development (i.e., Leaf_0 to Leaf_5) and reached maximum at the "Leaf_5" stage in *G. gynandra*. The same gene copies were also found to be highly expressed in *G. gynandra* cell type-specific data (Figure 7D), and generally showed a preferential expression pattern according to their expected function and protein localization. For example, the expression of *BCA4* and *PEPC2* are high in mesophyll cells, *NAD-ME1/2* and *mMDH1* are high bundle sheath cells, while *AspAT* and *ALAAT1* are high in both cell types. The highly expressed duplicated gene copies in these C_4 gene families therefore can be considered as the functional C_4 genes. Among the 43 key C_4 gene families analyzed in this paper, we did not observed any missing genes in the *G. gynandra*. Additionally, since we now added subgenome fractionation bias (see response to your Comment 3), we also analyzed subgenome gene expression bias in the *G. gynandra* and *T. hassleriana* genomes. Overall, we observed the subgenome dominance pattern in both species (Figure 7A and B). These analyses now have been added to new section "Comparative tissue/organ- and cell type-specific gene expression analysis uncovered subgenome dominance and the upregulation of functional C_4 duplicated gene copies" in the main text lines 505-553, Figure 7 and Supplemental Data Set 5.

Reviewer #2:

First and foremost, I want to apologize to the reviewers for the delay in submitting my review. A series of unexpected events have redirected my attention over the past few weeks. I'm very sorry that my personal matters impacted you - not receiving reviewer feedback within a reasonable timeframe. The delay in you receiving feedback is entirely on me; the editors and staff at TPC have done their absolute best to expedite this process.

In this manuscript, the authors present a new genome sequence for *Gynandropsis gynandra* and various analyses aimed at uncovering how C4 photosynthesis evolved in Cleomaceae. The data suggest that gene duplications, both polyploidy and small-scale duplications, and biased gene retention patterns towards certain photosynthesis related pathways may have contributed to the evolution of C4 photosynthesis. This is interesting given that previous studies of other systems, including the evolution of C3 to CAM in pineapple, suggested that this trait evolved largely from the evolution of novel cis-regulatory elements and not gene duplications. Thus, this species will serve as an excellent additional model to further dissect the evolution of this important agronomic trait. Furthermore, they provide clear evidence that *T. hessleriana* underwent an additional hybridization and polyploid event not shared with *G. gynandra*. This will be of great interest to the Brassicaceae-Cleomaceae research community. Overall, this is a well-written manuscript for a broad audience. This genome, and the exciting findings presented here, will serve as a useful resource for the plant science community.

We would like to thank the reviewer for the positive feedback and suggestions to improve our manuscript. Also, it is not necessary to apologize for the delay in receiving the review, we are appreciate the helpful feedback and suggestions.

Comment 1. Line 182: "gene families". Orthogroups are not equivalent to gene families. A single orthogroup may not contain all members of a particular gene family (as described in TAIR10 by gene family experts), and may likely contain genes from multiple gene families. I have no concerns about these analyses themselves, but rather calling them "gene families". I would recommend referring to them as 'orthogroups'.

We agree with the reviewer, and thus we now refer to them as "orthogroups" throughout the manuscript.

Comment 2. Line 269-270: The topology suggests that the tetraploid intermediate is not shared by T. hassleriana and G. gynandra - assuming those genes are derived from a polyploid event - OR that the tetraploid event is an autopolypoid event. Otherwise the homoeologs from each diploid progenitor within each polyploid would be sister to one another. As noted by the authors later, it is far more parsimonious, given data from previous studies that this event is shared. Thus, I would lean towards it being an autopolyploid event followed by the hybridization of a more distantly related diploid resulting in the WGT. ThBCA4-1 is sister to the tetraploid clade.

The phylogenetic resolution of the *BCA4* gene is low in Cleomaceae, with support values of 0.33-0.53. To elucidate the evolutionary history of polyploidization in the family, we now conducted a species-tree type analysis for

syntenic genes with a 1:2:3 pattern, which fully supports a polyploidization event shared by *G. gynandra* and *T. hassleriana* and subsequent addition of +1x to the *T. hassleriana* lineage (see our earlier extensive reply to Reviewer#1, Comment 3). Because we also observed subgenome biased fractionation patterns in *G. gynandra* and *T. hassleriana* (Figure 3A and B) which are typically reported for species derived from hybridization/allopolyploidization, thus we conclude the WGD to have been an allopolyploid event. We moved the *BCA4* gene tree to Supplemental Figure S8, to illustrate the single-gene phylogenetic tree approach in comparison to species-tree approach.

Comment 3. Line 361: The community standard is typically to apply a cutoff at Ks =3, maybe Ks=4, beyond that its largely just noise (saturation). I'm very skeptical of the interpretation and/or further analysis of any data gathered beyond the Ks=4 cutoff. Important to note - applying a new cutoff would not alter the primary conclusions. This is likely why you are seeing a bimodal distribution.

We agree with the reviewer on this point. We have now revised the related text and Figure 4, taking a *Ks* = 3 as a cutoff. This indeed does not change the primary conclusions, and highlights the different distribution of *Ks* values of transposed/dispersed gene pairs between *G. gynandra* and *T. hassleriana*. Text related to this has been revised.

Comment 4. Thank you to authors for making the genome available to the reviewers. Will germplasm of this reference line be made publicly available? Doing so - I believe will not only permit others to help dissect this important trait, but will permit them to truly leverage this new genome, which will also result in far more citations for this manuscript. Equally important community resource; genome and germplasm.

Thank you very much for this suggestion. Seeds from the *G. gynandra* "GYN" accession will be available upon request from the corresponding author. We added this information to section "Data availability/ Accession numbers".

Minor comments:

1. Lines 47-48, Line 336: "super-scaffolds" or "pseudo-chromosome" replace with more commonly used term "pseudomolecules"

We now used "pseudomolecules" throughout the manuscript.

2. Line 51: "We revealed" replace with "Our analyses revealed ..."?

This is now revised.

3. Line 78: "... an orphan or neglected species ... " replace with "a orphan or neglected crop" or "crop species". "Orphan species" sounds odd. The vast majority of angiosperm species lack "research efforts to develop genetic and genomic resources".

The phrase "an orphan or neglected species" has been replaced with "an orphan or neglected crop".

4. Line 144-145: "from an inbred line". How many generations of selfing?

As described in line 642 (Methods): the inbred line was inbred by hand-pollination for four generations. We now also mentioned this at the beginning of the Results (line 157-158).

5. Line 209: Add Figure 2 here.

Figure 2 is cited now.

6. Line 355-356: TAN, PRO, TRA and DIS. No need to abbreviate -- this is going to be confusing to the average reader. WGD is fine. Others spell out.

TAN, PRO, TRA and DIS were spelled out now.

7. Line 367-368: Yes, high birth/death dynamics such as been shown in various previous studies over the past two decades. Maere et al., 2005 (PNAS) is among the best studies on this topic.

We thank the reviewer for the suggested reference. We now discussed the suggested paper in line 396-398.

Reviewer #3:

In the manuscript by Hoang et al. they present the first reference genome for Gynandropsis gynandra, a C4 Cleomaceae species that is widely grown as a leafy vegetable. Along with presenting the assembly itself, the authors also use comparative genomics to infer the history of genome duplication in this group, and investigate how gene duplication may have played a role in C4 evolution in this species. Overall, the manuscript is well written and describes some interesting patterns around the order of genome duplication, although I am not sure there is enough besides this to warrant publication in The Plant Cell. Below I will detail some specific comments.

Comment 1. Whilst I enjoyed reading the manuscript it overall felt like publishing the genome is the main aspect of this paper, and the additional analyses are an accessory. This was highlighted in the second paragraph which listed all the things that could be done with a genome for this species, rather than setting the scene for the science the researchers were using this genome for. I would suggest modifying the intro (and elsewhere in the manuscript) to make it read less like a genome release note. For example, there is only a cursory introduction to previous research into gene duplication and C4 evolution despite this being one of the main focuses of the paper.

We would like to thank the reviewer for offering us insightful comments and suggestions. We have now revised the introduction (lines 120-133) and included previous research into gene duplication and C_4 evolution, as suggested. We added new analyses and revised other parts the manuscript according to your suggestions and from the other two reviewers. We hope that the changes we made sufficiently satisfy your suggestions.

Comment 2. It is evident that the genomes of the other Cleomaceae species are not as good (*T. hassleriana* & *C. violacea*), as evidenced by the fragmented synteny plots (Fig. 2 B, D, F) and at present it is unsure (and not discussed) how this might affect the results.

Tarenaya hassleriana
Number of scaffolds: 12,249
Scaffold N50: 1.6 Mb
Number of contigs: 37,0732
Contig N50: 22.4 kb

C. violacea
Number of scaffolds: 1,099
Scaffold N50: 2.2 Mb

For example it would be nice to see the BUSCO scores (e.g. at 12.9% duplication in *Gynandropsis gynandra* [**Table S5**] is this higher or lower than the other species) for all the assemblies used so that these can be compared. This can be done for the assembly and annotations to see how they compare for all species. I would be less worried about the genome wide patterns (except for when looking at tandom/proximal repeated), but potentially this could affect the proportion of genes retained after the genome duplication in the C_4 genes. You could look at doing an analysis on the individual reads themselves to double check if the genes are truly absent, or whether this is a result of miss-annotation / assembly.

We thank the review for this suggestion. We reran BUSCO both on genome assemblies and annotations to compare the score and duplicated BUSCOs between species used in this study (Supplementary Figure S12, please also see our earlier response to Reviewer #1's Comment 1). The previous BUSCO results were based on extracted transcript sequences annotated for each genome, and did not truly reflect the duplication but the maximal completeness of genome annotation. It is true that the genomes of *T. hassleriana* and *C. violacea* are more fragmented (i.e., based on N50 and scaffold number statistics). However, their BUSCO completeness are actually higher than that of *G. gynandra* (99.3% in *C. violacea*, 98.5% in *T. hassleriana* compared to 98.2% in *G. gynandra*), suggesting that the gene content is represented well in these genomes. Among six genome assemblies from the Cleomaceae and Brassicaceae, the duplicated BUSCOs are highest in *B. rapa* (14.5%), followed by *T. hassleriana* and *G. gynandra*, but not very different between the latter two genomes (6.2% and 6.1%, respectively). In this analysis, BUSCO employed Metaeuk gene predictor and did not rely on the available annotation. Since the *T. hassleriana* genome contains 3x genomic equivalents, while *G. gynandra* has only 2x, this indicates that *T. hassleriana* has a higher gene loss rate. This is consistent with the new analysis of gene fractionation bias between intraspecies and

interspecies subgenomes (see response to Reviewer #1's Comment 3, and Figure 3A, B).

Despite being more fragmented, the numbers of genes derived from recent WGD events (based on synteny) from the three Cleomaceae species are what we would expect, highest in *T. hassleriana*, followed by *G. gynandra* and then *C. violacea* (Supplemental Figure S10). Since tandem/and proximal are genes located near each other on the same chromosome/scaffold, the detection of these gene duplication modes was unlikely affected, unless they are present but were not annotated. However, having fragmented genomes might affect the detection of transposed and dispersed duplicated genes (e.g., WGD genes being identified as transposed or dispersed). To overcome this, in our synteny-based analyses and gene duplication mode detection, the minimum syntenic block size was set to 30 and 4 colinear genes for MCscan/JCVI; 4 genes (window size of 40 genes) for SynFind and 5 genes for MCscanX running in DupGen_Finder pipeline (see Methods and also response to your Minor Comment 20). These are all default parameters for each of the packages. Because of these and the new BUSCO score results, we think that the our results were unlikely affected.

Comment 3. I am still a little confused about the genome sizes of the group, the below sizes are from the manuscript [lines 139 - 142].

So based on Figure 3D:
1x C. violacea = 280 Mb
2x G gynandra = 930 Mb
3x T. hassleriana = 290 Mb <- so despite experiencing a genome duplication and hybridization the whole genome is only 10 Mb (3.5%) larger? Maybe this is just confusion around how the values are reported in the text, are these 1C values taking into account known ploidy?

If not: At present this difference is attributed to TE expansion (wonder if this played a role in gene duplication/retention).. But if you were to assume that C. violacea has the diploid genome size 1C, then the duplication in the ancestor at Gg-α would be ~560Gb, and Th-α would result in a genome of 840Gb. More remarkable would be the rapid genome reduction in T. hassleriana.

We apologize for this confusion. It is important to make clear here that all three species are diploid (2n) due to the diploidization/genome rearrangement process after the recent whole-genome duplication event. The genome sizes reported above are 1C values of a haploid nucleus (also known as "n"). Since these genomes underwent several polyploidy events, in the previous version of the manuscript, we used "x" to refer to one ancestral genome equivalent within each polyploid genome. To avoid any further confusion, we now use "x" to indicate levels of duplication (e.g., for a WGD/WGT event), and "genome equivalent, GE" relative to the common ancestor to indicate polyploidy level (e.g., in a diploid genome) (Figure 3).

Our analyses suggest that *T. hassleriana* might have undergone extensive genome reduction, especially in the *Th*MF$_2$ subgenome (Figure 3A, B), whereas *G. gynandra* has higher repetitive content likely due to the recent burst of long terminal repeat-retrotransposon (LTR-RT) insertion (Zhao et al., 2022).

Comment 4. "Here, we present a high-quality genome sequence of G. gynandra" [line 47]
I agree that the reference assembly you present is good, and some of the statistics (e.g. N50 and BUSCO presence) are impressive, but these are pretty standard now given the widespread adoption of PacBio HiFi data. Also, other statistics are less impressive, for example the %ambigious bases and the proportion of the actual genome assembled:

~10% N (Table S2)
Predicted assembly size: 0.93 Gb
Flow cytometry size: 1.06 - 1.23 Gb (from cited papers)
Assembly size: 0.740 Gb
Proportion of genome sequenced based on Predicted assembly size (& 10% N):
(0.740 * 0.90) / 0.93 = 71.6 % of genome assembled

Proportion of genome sequenced based on Max flow cytometry size (& 10% N): 54.1% assembled
(0.740 * 0.90) / 1.23 = 54.1 % of genome assembled

The only thing I suggest changing is deleting "high-quality" from line 47 & 193. Also, it would be good to report some of the less favourable statistics in Table 1, e.g. %N, full BUSCO results, number of scaffolds at the various steps before scaffolding <- particularly useful for readers who want to assess the impact of using Hi-C and 10X.

We agree with the reviewer and have removed the word "high-quality". We now added statistics for three genome assembly versions to allow assessing the impact of using 10X Genomics and Hi-C. This includes the impact on scaffold number, assembly size, %N, BUSCO completeness score and mapping back rates (see Supplemental Tables S2 and S3). We now added more detailed statistics in the main text (lines 150-175).

Comment 5. "As a result, phylogenetic or syntenic approaches might not be able to differentiate the gene copies that derived from these closely sequential events" [line 281 - 282] <- is this not worrying for the other analyses if you are unable to assign true parology? In particular I am thinking about the Ks for the transposed and dispersed genes (Figure 4E) which shows a bimodal relationship with a lot of old and a lot of new ones. These two categories are also the genes where this problem of ensuring you have the true ortholog is going to be hardest as there is no synteny relationships <- are you sure the excess old ones are not just a result of comparing paraologs?

The quoted sentence above is related to the tree topological incongruence when using the single-gene phylogenetic approach for the placement of WGD/WGT events in the Cleomaceae. This is because these events occurred very close to the speciation of the two species, *G. gynandra* and *T. hassleriana*. We addressed this comment together with Comment 3 from Reviewer #1 and Comment 2 from Reviewer #2. A high resolution large-scale phylogenetic tree based on syntenic genes from four syntenic blocks that have 1 *Cv* :2 *Gg* :3 *Th* syntenic pattern is now provided (Figure 3D). We discussed the above issue which is likely due to single-gene approach that we employed in the previous version (lines 281-290). We argue that, for species-tree reconstruction, by using genes on syntenic blocks, we could make use of the shared evolutionary history of collinear genes and increase phylogenetic resolution compared to single-gene approach.

Regarding *Ks* values of the transposed/dispersed gene pairs: By definition, transposed gene pairs are non-WGD, non-tandem, non-proximal, and consisted of one ancestral and one non-ancestral copies. Dispersed gene pairs are non-WGD, non-tandem, non-proximal, non-transposed and with a BLASTP hit. It is therefore expected to see a bimodal relationship in their *Ks* distribution. A similar case was reported previously for the pear genome (Qiao et al., 2018). Because of this, in analyses related to detecting the recent WGD/WGT events (Figure 1C) or species-tree phylogenetics (Figure 3D), we included only WGD genes identified through a synteny approach. Additionally, following a suggestion by Reviewer #2 comment 3, we remade Figure 4E, taking a cutoff of *Ks* = 3.

Comment 6. Are these duplicates all used in the current C4 pathway?

We thank the reviewer for pointing this out. In the previous manuscript version, the genes used in C_4 pathway were only derived from *SynFind* analysis (Tang et al., 2015) which were syntenic genes/blocks across the genomes (i.e., *C. violacea* vs. *G. gynandra* and *T. hassleriana*). Gene duplication modes were analyzed by *DupGen_finder/MCscanX* (Qiao et al., 2019) for each genome and added onto the SynFind result to infer the contribution of gene duplication modes to each gene family in each genome. This means that, in Figure 6F, except WGD genes (syntenic, green color), genes labeled as other duplication modes (single-gene duplication) actually contain more copies than actually shown on the previous version of the figure. To account for all duplication modes, in the revised version, we included WGD genes detected by SynFind and single-gene duplicates from *DupGen_finder.* These genes were also used for tissue- and cell type specific expression analysis in Figure 7C and D.

Minor points:

1. [line 45 - 46] To someone now familiar with the system this line could be a little unclear, I would make it more abundantly obvious that *G. gynandra* is the C_4 species that is compared to C_3 Arabidopsis.

This now has been revised (line 44).

2. [line 73] I always think of Chlorophytum when people say 'spider plant' .. I suppose that's the trouble with common names. It is up to the authors how they want to address this (or even if they do), but one potential solution would be to add: "Gynandropsis gynandra (shares the common name 'spider plant' with a number of unrelated species...)"

This now has been revised (lines 71-72).

3. [line 77 - 79] should this be an orphaned or neglected "crop" ? Many widespread species lack genomics resources.

Revised (line 77). Also to address a suggestion from Reviewer #2.

4. [line 92] can you give an actual age estimate for the divergence time between Arabidopsis and G. gynandra from published work?

Cleomaceae (including *G. gynandra*) diverged from the Brassicaceae (including *A. thaliana*) about 40 million years ago (Edger et al., 2015; Mabry et al., 2020).

5. [line 91 - 95] this sentence is hard to follow, rework. I would explicitly say why being close to Arabidopsis helps, and split this sentence into two.

This now has been revised (lines 88-95, and 125-133). The sentence has been split into two, and we added "well-studied" in front of Arabidopsis to indicate how and why being close to it helps. This is also discussed at the beginning of Discussion section (lines 570-576).

6. [line 112 - 113] Make it clear this increased efficiency is only in certain environments.

This now has been revised (lines 113-114). We added "in certain environments, for example, in warm, sunny and dry regions (Sage, 2004; Bayat et al., 2018)" to the sentence.

7. [line 133 - 134] states "The results suggest potential explanations as to why C4 photosynthesis evolved in *G. gynandra* but not in *T. hassleriana* which involved differential gene duplication and gene retention between the two species." This makes me wonder about the biogeography of the two species, I mean is C4 an advantage where T. *hassleriana* grows? I don't think this shows why it evolved in *G. gynandra*, more of what the result was because they evolved it (i.e. retained more C4 gene duplicates etc.)

Thank you very much for asking this interesting question. While we do not have enough evidence to conclude the origins of the two species and how they evolved, it could be true that biogeographical conditions played important roles in the evolution of C_4 species found in *G. gynandra* but not in *T. hassleriana*. It is known that the distribution of C_4 species is generally correlated with temperature or aridity (combination of temperature and evaporative potential) (Ehleringer et al., 1997). *Gynandropsis* is widely distributed in Asia, and *Tarenaya* is found more in the New World (for example, Brazil) (Bayat et al., 2018; Parma et al., 2022). Since we are uncertain about this, we therefore decided not to discuss this in the manuscript.

8. [line 140] report the range of flow-cytometry estimates.

We added the range of flow-cytometry estimates, 1.1-1.2 Gb based on previous reports (Omondi et al., 2017; Parma et al., 2022) in line 152 as your suggestion.

9. [line 142] how were these estimates generated? flow-cytometry estimates?

The genome sizes of *T. hassleriana* and *C. violacea* are from data reported in Cheng et al. (2013) and downloaded from Phytozome (https://phytozome-next.jgi.doe.gov/info/Cviolacea_v2_1), respectively.

10. [line 285] the part " and possibly experience adaptive selection.." is some what leading as no evidence of this is provided

We now replaced the related single-gene tree with a large-scale phylogenetic species-tree (see responses to Reviewer #1's Comment 3 and Reviewer #2's Comment 2). The *BCA4* gene tree was moved to Supplemental Figure S8, to illustrate the differences between single-gene phylogenetic tree approach and species-tree approach. Additionally, we now provided new analysis to highlight which duplicated gene copy in each gene family is the functional C_4 gene (see Figure 7). In this, the *BCA4* gene copy corresponding to the longest branch was found to be the upregulated gene copy among the *BCA4* duplicated gene copies.

11. [line 351] The use of the Fisher's LSD: was an ANOVA also conducted? Was this significant?

Yes, with ANOVA and LSD post-hoc test. $p < 0.05$, which is considered as "significant". We now revised this to read as "ANOVA Fisher's LSD post-hoc test".

12. [line 351-352] "These data suggest that WGD-derived genes are generally more conserved across these species." This seems a little bit of a jump, guide the reader through a little easier.

This is now revised and also cited reference source (line 375-378).

13. [Paragraph 353-380] this paragraph is incredibly dense with acronyms which makes it somewhat hard to digest. Also "youngest" and "oldest" should be "smallest" and "largest"

This is now revised and all acronyms were spelled out as per Reviewer #2's suggestion.

14. [line 373 - 380] Yes, using the ka/ks > 1 is stringent, but anything less conflates a relaxation in purifying selection with a shift to positive selection. Would it be possible to look at site codeml models to look at selection? I am aware this is quite a bit of work.

We thank the reviewer for this suggestion. We followed a pipeline previously used in Qiao et al. (2019), and the results are consistent with that reported in Huang et al. (2021) using transcriptome data. While the suggested analysis might add some interesting results to the paper, considering the amount of work, we decided not to include this analysis.

15. [line 379-240] "Collectively, this indicates that the two genomes might have been subjected to different selection pressure" I mean this is kind of expected no? They are different species.

This sentence is now revised (line 409-410). It now reads as follows: "Collectively, this highlights different selection pressure that the two genomes might have experienced."

16. [line 504-505] What about a dosage effect in the origin of C4?

Dosage effect by gene copy number (through WGD) on gene expression of C_4 genes *PEPC* and *PCK* was reported previously in the grass genus *Alloteropsis* (Bianconi et al., 2018). We analyzed gene expression of selected genes from the C_4 pathway including *PEPC* and *PCK* in *G. gynandra* in Figure 7 and Supplemental Data Set 5. Generally, the results showed that among duplicates of a C_4 gene family, one of the duplicates is expressed much higher than the others. The dosage effect was not clearly observed in this case.

17. [line 515 - 516] BUSCO score of what? Complete?

Yes, it is BUSCO score. This has been corrected.

18. [Methods] Software versions missing throughout

Software versions were checked and added throughout the manuscript. Please note that version information is not available for some software or packages. In those cases, we added the time we accessed them.

19. [line 605] was the optimal k-mer of 99 used for assembly? .. just out of interest, I don't suggest repeating all analyses etc if this is not the case as you assembly statistics already show you have a good assembly.

De novo assembly was previously done by BGI using SOAPdenovo2 and k-mer 63. We added this information to line 651-652.

20. [line 652] how were the synteny thresholds for the number of genes decided?

For Macrosynteny (e.g., Figure 3C), 30 colinear genes are default setting of Mcscan's user's manuals (https://github.com/tanghaibao/jcvi/wiki/MCscan-%28Python-version%29). For microsynteny by SynFind and Mcscan, this was set to 4 genes (default settings). We also tested with a minimum number of 6 genes in SynFind, however, the settings with 4 genes worked better.

21. [Table 1] GC% seems low. Does this exclude the ambiguous bases in this estimate?

The GC content % was calculated by excluding the ambiguous bases. The base compositions (A:C:G:T) of the final *G. gynandra* assembly v3.0 are 207,470,131 : 125,575,071 : 125,551,299 : 207,707,396. When compared with that of other related species in Cleomaceae and Brassicaceae, the GC content % of *G. gynandra* is within the expected range, see a table below.

Genome	Base composition				GC content %
	A	C	G	T	
A. arabicum	61,918,930	32,905,865	32,902,235	61,928,974	34.70
A. thaliana	38,223,602	21,551,439	21,528,650	38,177,852	36.06
B. rapa	110,867,586	64,686,546	64,624,139	110,884,929	36.83
C. violacea	85,500,427	54,028,532	53,997,914	85,512,460	38.71
G. gynandra	**207,470,131**	**125,575,071**	**125,551,299**	**207,707,396**	**37.69**
T. hassleriana	66,414,979	43,168,103	43,164,543	66,402,597	39.39

22. [Table S5] add the name of the lineage data set

The name of the lineage data set, embryophyta_odb10, was added. Note that, this is now Supplemental Table S7.

23. [Figure 3] What topology is shown? branch lengths scale needed. Hard to tell who informative these markers are without a scale, do you resolve the tree if you use introns for *BCA4*? Is there the potential for recombination between the copies?

Thank you very much for pointing this out. We now provide a large-scale phylogenetic species tree instead of the previous single-gene tree (see responses to Reviewers #1 Comment 3 and Reviewer #2 Comment 2. Because single genes often showed low resolution and inconsistent topologies, we used genes that are from four syntenic blocks on different chromosomes. For comparison, we moved single gene trees into Supplemental Figure S8. Branch length scales are now provided for each tree.

24. [Figure 5] Do you correct for multiple testing?

In the previous version, only Fisher Exact test (*p* ≤ *0.05*) was used, without multiple testing correction. However, we now included KEGG pathway enrichment using a new cutoff *FDR*-corrected *p-value* ≤ 0.05 for Figure 5. For Supplemental Data Set 3, we included all KEGG pathways of a cutoff of *p* ≤ 0.05 and *FDR* ≤ 0.3. Text and supporting data related to this have been updated.

TPC2022-RA-01103 1st Editorial decision – *accept with minor revisions* **December 16, 2022**

We have received reviews of your manuscript entitled "The genome of Gynandropsis gynandra provides insights into whole-genome duplications and the evolution of C4 photosynthesis in Cleomaceae." On the basis of the advice received, the board of reviewing editors would like to accept your manuscript for publication in The Plant Cell. I do agree with the comments of reviewer #2, but I leave it up to you to decide whether you want to alter Figure 1C and remove your local server nicknames from Line 852.

-- Reviewer comments:

TPC2022-RA-01103R1 1st Revision received **December 17, 2022**

Reviewer comments on previous submission and **author responses**:

Reviewer #2:

The authors addressed each of my previous concerns, and nicely addressed the concerns raised by the other reviewers. The majority of previously raised concerns were shared by multiple reviewers. The new genomic resources and preliminary research reported here should serve as an important foundation into future research efforts to understand the evolution of C4 photosynthesis (to guide future genetic engineering efforts). A key trait for generating more resilient crops will likely be C4 photosynthesis, a component to combat the ongoing and future impacts of global climate change. Overall, manuscript is much improved compared to the previous version, and should be of general interest to a broad readership.

The authors would like to thank the reviewer for your constructive comments and suggestions during the revision of this manuscript.

Two minor remaining comments -

Comment 1. Figure 1 Panel C - Apply a *Ks* value cutoff of 4.0. We both agreed that beyond *Ks* of 4 is noise, and now the figure consists of unnecessary empty space.

Following the reviewer's suggestion, we have now applied a *Ks* cutoff of 4.0 to our fitting Gaussian Mixture Model (GMM) analysis and revised Figure 1C. Since this did not change the *Ks* peaks corresponding to WGD events of different ages or data interpretation, we only revised the text related to *Ks* cutoff ≤ 4.0 in the Methods (line 820).

Comment 2. Line 852. Not sure that server nicknames "MARY" and "MERIAN" need to be provided. I understand it's fun, but the PCR machines used for generating the Illumina libraries may feel left out.

We agree with the reviewer's suggestion and removed the server nicknames in the revised version (line 853). The sentence now reads as follows: "All analyses in the Linux environment were conducted on local servers running Ubuntu 16.04.6 LTS hosted by the Biosystematics Group at Wageningen University, the Netherlands."

Reviewer #3:

This is the second time I have reviewed this manuscript and the reviewers have done a good job in responding to the reviewers comments and feel the manuscript is much improved, with the expanded introduction and additional analyses welcome. The figures are also looking great.

We appreciate your constructive comments and suggestions during the revision of this manuscript.

**TPC2022-RA-01103R1 2nd Editorial decision – *acceptance pending***	**December 29, 2022**

We are pleased to inform you that your paper entitled "Title" has been accepted for publication in The Plant Cell, pending a final minor editorial review by journal staff. At this stage, your manuscript will be evaluated by a Science Editor with respect to scientific content presentation, compliance with journal policies, and presentation for a broad readership.

**Final acceptance from Science Editor**	**Jan. 18, 2022**
